# Potent Protease Inhibitors of Highly Pathogenic Lagoviruses: Rabbit Hemorrhagic Disease Virus and European Brown Hare Syndrome Virus

**DOI:** 10.1128/spectrum.00142-22

**Published:** 2022-06-29

**Authors:** Krishani Dinali Perera, David Johnson, Scott Lovell, William C. Groutas, Kyeong-Ok Chang, Yunjeong Kim

**Affiliations:** a Department of Diagnostic Medicine & Pathobiology, College of Veterinary Medicine, Kansas State University, Manhattan, Kansas, USA; b Computational Chemical Biology Core, The University of Kansas, Lawrence, Kansas, USA; c Protein Structure Laboratory, The University of Kansas, Lawrence, Kansas, USA; d Department of Chemistry and Biochemistry, Wichita State University, Wichita, Kansas, USA; University of Prince Edward Island

**Keywords:** European brown hare syndrome, protease inhibitor, calicivirus, lagovirus, rabbit hemorrhagic disease

## Abstract

Rabbit hemorrhagic disease (RHD) and European brown hare syndrome (EBHS) are highly contagious diseases caused by lagoviruses in the *Caliciviridae* family. These infectious diseases are associated with high mortality and a serious threat to domesticated and wild rabbits and hares, including endangered species such as riparian brush rabbits (*Sylvilagus bachmani riparius*). In the United States (U.S.), only isolated cases of RHD had been reported until Spring 2020. However, RHD caused by GI.2/rabbit hemorrhagic disease virus (RHDV)2/b was unexpectedly reported in April 2020 in New Mexico and has subsequently spread to several U.S. states, infecting wild rabbits and hares and making it highly likely that RHD will become endemic in the U.S. Vaccines are available for RHD; however, there is no specific treatment for this disease. Lagoviruses encode a 3C-like protease (3CLpro), which is essential for virus replication and a promising target for antiviral drug development. We have previously generated focused small-molecule libraries of 3CLpro inhibitors and demonstrated the *in vitro* potency and *in vivo* efficacy of some protease inhibitors against viruses encoding 3CLpro, including caliciviruses and coronaviruses. Here, we report the development of the enzyme and cell-based assays for the 3CLpro of GI.1c/RHDV, recombinant GI.3P-GI.2 (RHDV2/b), and GII.1/European brown hare syndrome virus (EBHSV) as well as the identification of potent lagovirus 3CLpro inhibitors, including GC376, a protease inhibitor being developed for feline infectious peritonitis. In addition, structure-activity relationship study and homology modeling of the 3CLpro and inhibitors revealed that lagovirus 3CLpro share similar structural requirements for inhibition with other calicivirus 3CLpro.

**IMPORTANCE** Rabbit hemorrhagic disease (RHD) and European brown hare syndrome (EBHS) are viral diseases that affect lagomorphs with significant economic and ecological impacts. RHD vaccines are available, but specific antiviral treatment for these viral infections would be a valuable addition to the current control measures. Lagoviruses encode 3C-like protease (3CLpro), which is essential for virus replication and an attractive target for antiviral drug discovery. We have screened and identified potent small-molecule inhibitors that block lagovirus 3CLpro in the enzyme- and cell-based assays. Our results suggest that these compounds have the potential for further development as antiviral drugs for lagoviruses.

## INTRODUCTION

**FIG 1:**
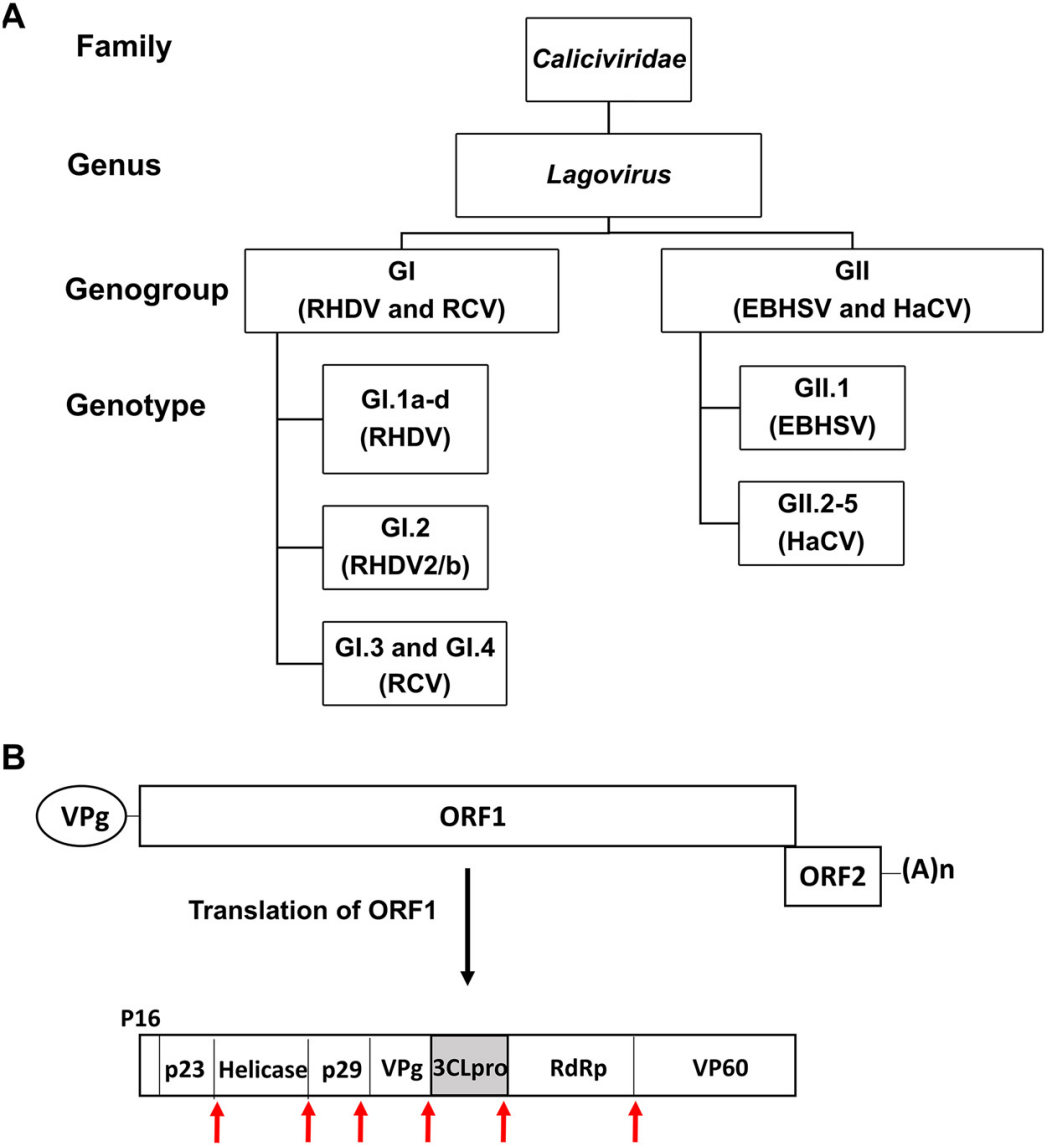
Classification and genomic organization of lagoviruses. (A) The *Caliciviridae* family contains 11 genera including the *Lagovirus* genus. Two main genogroups GI and GII in the *Lagovirus* genus further divide into several genotypes. (B) Genome organization of RHDV and EBHSV. The genome contains two overlapping open reading frames (ORF), ORF1 and ORF2. ORF1 translates into a polyprotein, which is proteolytically cleaved to generate nonstructural proteins and the capsid protein (VP60). ORF2 codes for the minor structural protein VP10. The red arrows indicate the cleavage sites on the virus polyproteins that are processed by 3CLpro. The genomic and subgenomic RNA (not shown) have a virus-encoded protein VPg attached to their 5′ end and are polyadenylated at their 3’end. RHDV, rabbit hemorrhagic disease virus; RCV, rabbit calicivirus; EBHSV, European brown hare syndrome virus; HaCV, hare calicivirus; RdRp, RNA-dependent RNA polymerase.

Rabbit hemorrhagic disease (RHD) is a highly contagious viral disease of domesticated (farmed and pet) and wild rabbits with a high mortality, which can reach up to 70 to 100% (1, 2). RHD is caused by GI.1/rabbit hemorrhagic disease virus (RHDV) and GI.2/RHDV2/b, which belong to the genus *Lagovirus* in the *Caliciviridae* family (Fig. 1A) (3). GI.1/RHDV was first reported in 1984 in China among rabbits imported from Germany (4), and its variant GI.2/RHDV2/b emerged in 2010 in France (5). Since then, both GI.1/RHDV (contains GI.1a to GI.1d) and GI.2/RHDV2/b infections have become endemic in most parts of the world, including Australia, New Zealand, parts of Asia, and most of Europe, and in the last several years, GI.2/RHDV2/b has replaced GI.1/RHDV (1, 6–8). In the United States (U.S.), only sporadic, isolated incidences of RHD had been reported in domesticated rabbits until recently (9). However, GI.2/RHDV2/b infections in wild black-tailed jackrabbits (*Lepus californicus*) and wild cottontails (*Sylvilagus* spp.) were confirmed in the U.S. state of New Mexico in April 2020 (9) and have been spreading to multiple states among domesticated and wild rabbits, significantly diminishing the hope of eradication of RHD in the United States (10). Both GI.1/RHDV and GI.2/RHDV2/b can cause highly contagious viral hepatitis. The mortality rate is high for GI.1/RHDV (70 to 90%) and highly variable for GI.2/RHDV2/b (5 to 70%) infections (1, 11). The affected animals may die before showing clinical signs (peracute form) or exhibit lethargy, inappetence, neurological or respiratory signs, and fever followed by death within a few days after the onset of symptoms (acute form). Subacutely or chronically affected animals show jaundice, body weight loss, and lethargy and may die after a few weeks or may survive (1, 11). Although RHD caused by either genotype has a similar pathogenesis and clinical course, GI.2/RHDV2/b can cause disease in young (<2 months old) and adult rabbits and several hare species (12–15), while RHD caused by GI.1/RHDV is limited to adult European rabbits (*Oryctolagus cuniculus*). Young rabbits less than 8 weeks of age show age-dependent resistance to disease following GI.1/RHDV infection (1). Detection of GI.2/RHDV2/b in wild hare die-offs has been reported in several states in the U.S. since March 2020 (16). GI.2/RHDV2/b may also pose a serious threat to threatened or endangered wild rabbit species, such as riparian brush rabbits (*Sylvilagus bachmani riparius*), riverine rabbits (*Bunolagus monticularis*), pygmy rabbits (*Brachylagus idahoensis*), and hispid hares (*Caprolagus hispidus*) (also referred to as bristly rabbits) (17).

European brown hare syndrome virus (EBHSV) (GII.1 genotype), another lagovirus, is a highly pathogenic and contagious virus that mainly affects hares, such as European brown hares (*Lepus europaeus*) (18, 19), mountain hares (*Lepus timidus*) (18), and Italian hares (*Lepus corsicanus*) (20), although the eastern cottontail (*Sylvilagus floridanus*) was reported to be susceptible to spillover infection of GII.1/EBHSV (13). European brown hare syndrome (EBHS) was first detected in Sweden in the early 1980s and is endemic in many regions of Europe (21). GII.1/EBHSV causes fatal viral hepatitis with clinical signs similar to those of RHD, and the mortality rate of EBHS in naive hares in captivity ranges from 10% to 100% but is low in hares in endemic areas due to preexisting immunity (22). Rabbit calicivirus (RCV, GI.3, and GI.4 genotypes) and hare calicivirus (HaCV, GII.2 to GII.5 genotypes) are nonpathogenic members of the genus *Lagovirus* (Fig. 1A) and have been shown to undergo recombination with GI.1/RHDV, GI.2/RHDV2/b, and GII.1/EBHSV, contributing to strain diversity and evolution of lagoviruses (23–25). The frequent recombination site is the junction between the VP60 and RNA-dependent RNA polymerase (RdRp); thus, recombinant viruses carry the mix-and-match sets of the capsid and nonstructural genes originated from different genotypes or genogroups (25). Recombinant lagoviruses are designated as [RdRp genotype]P-[capsid genotype] (3). Phylogenetic analysis revealed that all reported GI.2/RHDV2/b viruses are recombinant viruses that contain orphan capsids and nonstructural genes donated from benign and pathogenic G1 lagoviruses (24, 25). A recent report identified a GII.1P-GI.2 virus in a hare, which indicates that recombination between two genogroups also occurs (23).

These lagoviruses are highly contagious, requiring a small number of virus particles for infection, and are very stable in the environment (1, 26). It was reported that RHDV can survive for up to 3 months in rabbit carcass (27) and can be transmitted through contact with bodily fluids and by fomites and mechanical vectors (1, 28). Therefore, it is very difficult to control viral transmission once it is spread to wild rabbit populations. Inactivated and myxoma virus-based live recombinant vaccines are approved for RHD (GI.1/RHDV and GI.2/RHDV2/b) in the European Union, although no vaccine is available for EBHS. In the U.S., a recombinant subunit vaccine for GI.2/RHDV2/b was recently issued emergency use authorization by The U.S. Department of Agriculture's Center for Veterinary Biologics in October 2021 (10). However, no specific treatment is available for RHD and EBHS, and only strict biosecurity practices and vaccinations are used for prevention and control of lagovirus infections.

Caliciviruses including lagoviruses have a single-stranded, positive-sense RNA genome, which is organized into two major open reading frames (ORFs) (Fig. 1B). The ORF1 encodes a polyprotein, which is processed by virus-encoded 3C-like protease (3CLpro) to release mature nonstructural proteins and the capsid protein VP60 (1). This virus polyprotein processing by 3CLpro is an essential step in virus replication and is thus considered a promising target for drug discovery for viruses that encode 3CLpro, such as caliciviruses and coronaviruses. In this study, we established a fluorescence resonance energy transfer (FRET) assay and a cell-based reporter assay to evaluate 3CLpro inhibitors and identified potent compounds against the 3CLpro of GI.1c/RHDV, GI.3P-GI.2 (RHDV2/b), and GII.1/EBHSV using the *in vitro* assay systems. Structure-activity relationship study and three-dimensional (3D) homology modeling of 3CLpro were also conducted to understand the structural basis for the potency of the compounds, which showed that structural requirements of compounds for the inhibition of 3CLpro are similar among caliciviruses, including lagoviruses.

## RESULTS

### Inhibitory activity of compounds against the 3CLpro of GI.1c/RHDV, GI.3P-GI.2 (RHDV2/b), and GII.1/EBHSV in the FRET assay.

**FIG 2:**
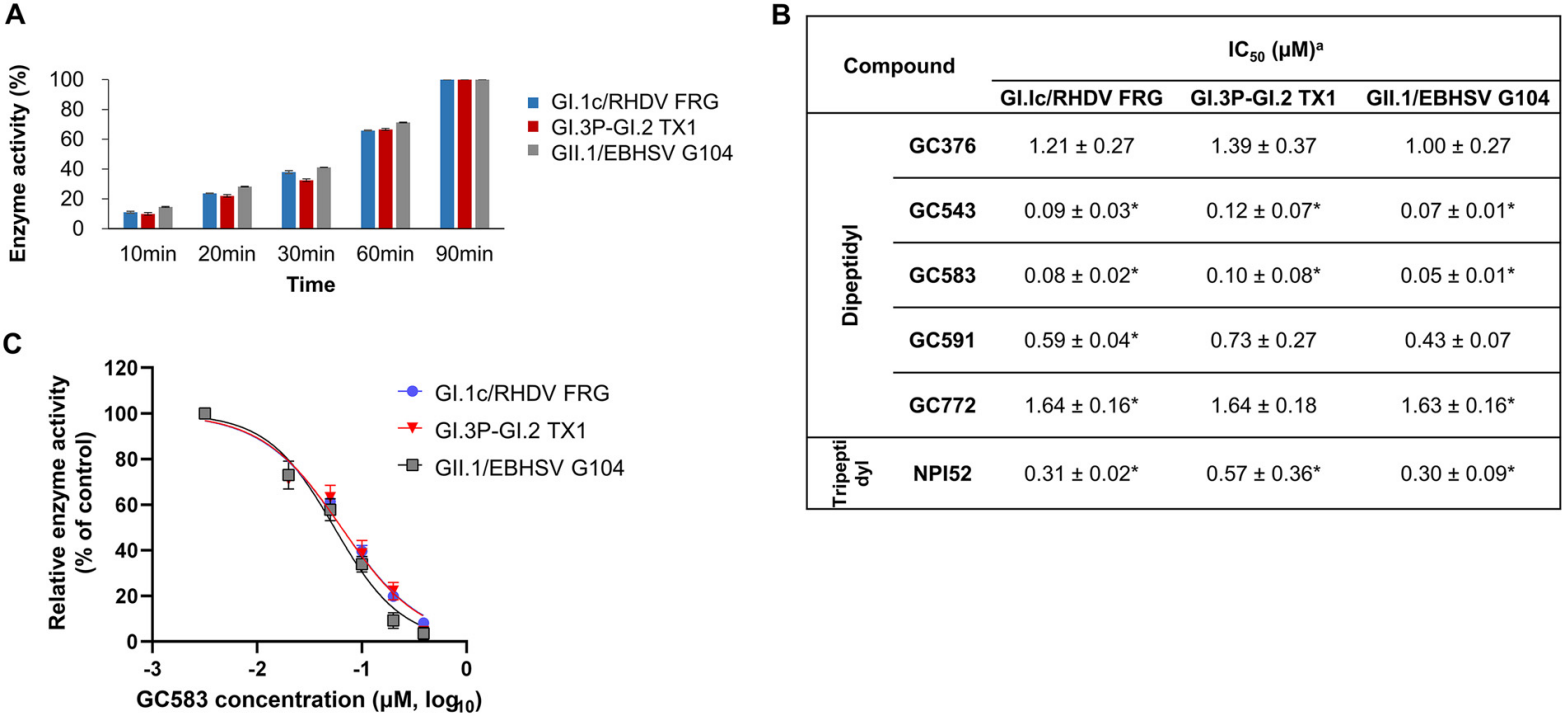
Activity of the compounds against the 3CLpro of GI.1c/RHDV1, GI.3P-GI.2 (RHDV2/b), and GII.1/EBHSV in the FRET assay. (A) Activities of the recombinant 3CLpro. Briefly, the 3CLpro was added to the assay buffer containing the fluorescence substrate, and the mixture was incubated at room temperature. The fluorescence signals were measured for up to 90 min, and the percentage activity of each 3CLpro was calculated. (B) The activity of compounds against 3CLpro in the FRET assay. Briefly, serial dilutions of each compound were added to assay buffer containing 3CLpro. After 30 min of incubation, the mixture was added to the assay buffer containing the FRET substrate. Raw fluorescence readings were obtained 30 min later, and relative fluorescence was calculated by subtracting substrate-only control from raw values to calculate the 50% inhibitory concentration (IC_50_) for each compound. ***^a^***Values are expressed as means ± standard errors of the means from at least three independent experiments. Asterisks indicate statistical significance compared with GC376 (*P* < 0.05). (C) Dose-dependent inhibition curves of GC583 against the 3CLpro of GI.1c/RHDV1, GI.3P-GI.2 and GII.1/EBHSV. Bars and symbols represent means and the standard errors of the means from at least three independent experiments.

Activities of the recombinant 3CLpro of GI.1c/RHDV FRG strain, GI.3P-GI.2 TX1 strain, and GII.1/EBHSV G104 strain were confirmed before the inhibition assays were conducted. Each 3CLpro showed a gradual increase in activity over time (Fig. 2A). Next, we evaluated the activities of compounds against each 3CLpro to determine the 50% inhibitory concentration (IC_50_) values (Fig. 2B). These compounds have the same skeletal structure with a glutamine surrogate at the P1 position and various functional groups at R_1_–R_3_ and different reactive warheads (R_4_) that interact with the cysteine residue (C104) at the catalytic site (Table 1). The IC_50_ values of GC376 are determined to be 1.21, 1.39, and 1 μM against GI.1c/RHDV, GI.3P-GI.2, and GII.1/EBHSV 3CLpro, respectively. GC376 is a dipeptidyl compound and contains Leu and benzyl ring at the R_3_ and R_1_ positions, respectively, and a bisulfite adduct warhead (R_4_) that converts to an aldehyde form (Table 1) (29). Substitution of the bisulfite adduct warhead and Leu at the R_3_ (GC376) with an aldehyde warhead and Cha (GC543) led to greatly increased inhibitory activity against all 3CLpro (*P* < 0.05). We have previously shown that the activity of compounds with bisulfite adduct is similar to those with aldehyde warheads against coronavirus, calicivirus, and picornaviruses in the FRET and cell-based assays (30, 31). Therefore, substitution of Leu with Cha at the R_3_ would be responsible for the observed increase in potency. Replacing the benzyl ring (GC543) with an *m*-benzyl ring (GC583) at the R_1_ slightly further increased potency against all tested 3CLpro, but no statistical significance was observed (*P* < 0.05). However, replacing the aldehyde warhead (GC583) with (C=O)CONH cyclopropyl (GC591) markedly decreased the inhibitory activities against all 3CLpro (*P* < 0.05), but this reduction in activity was not as profound as having Leu at the R_3_ (GC376). In GC772, the carbamate moiety of GC583 was replaced with a sulfonamide linkage, which decreased potency against all tested 3CLpro (*P* < 0.05). NPI52, a tripeptidyl compound, has homologous structural elements with GC376, except that NPI52 has an aldehyde warhead and an additional residue of 1-naththylalanine that corresponds to the P3 position. The inhibitory activity of NPI52 was more potent than GC376 and similar to GC543 and GC583 against the tested 3CLpro (*P* < 0.05). Overall, GC543 and GC583 have significantly improved activity compared to GC376 against the tested 3CLpro (*P* < 0.05). Dose-dependent inhibition curves of GC583 against the 3CLpro are shown in Fig. 2C.

**TABLE 1:**
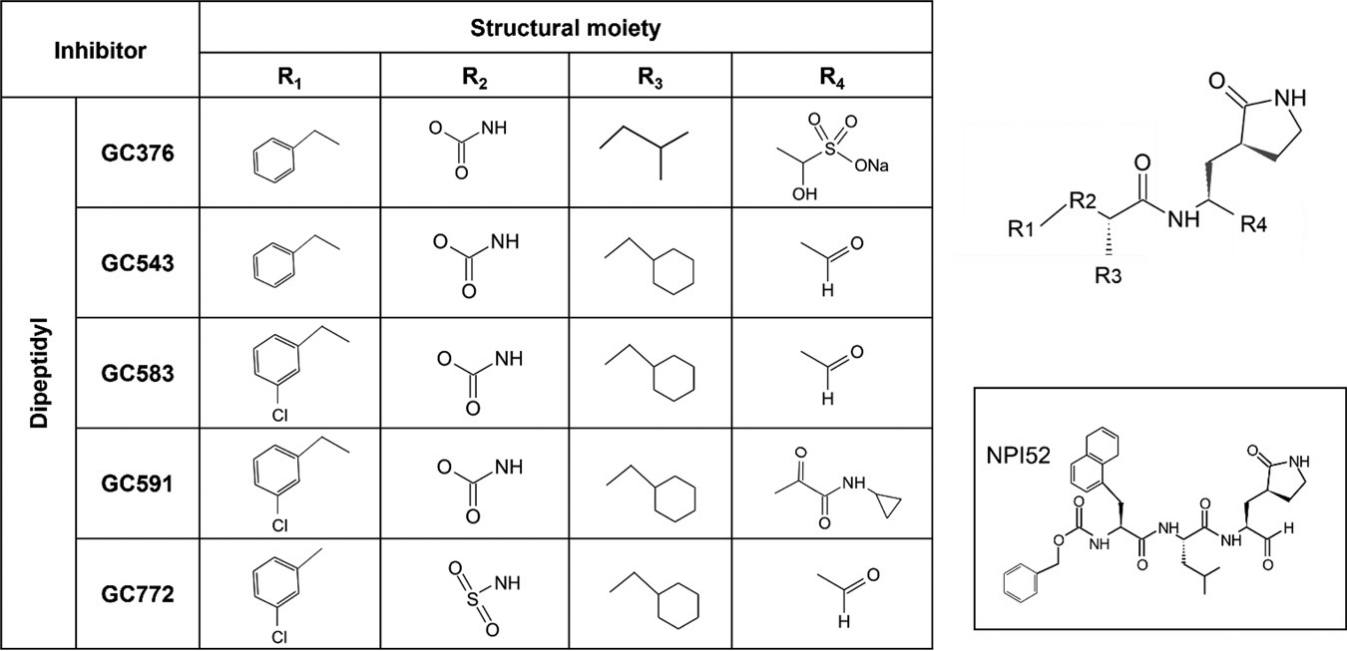
Structures of the inhibitors used in the assays^*a*^

a The structure of NPI52, a tripeptidyl compound, is shown in the box.

### Inhibitory activity of the compounds in a cell-based luciferase reporter assay.

**FIG 3:**
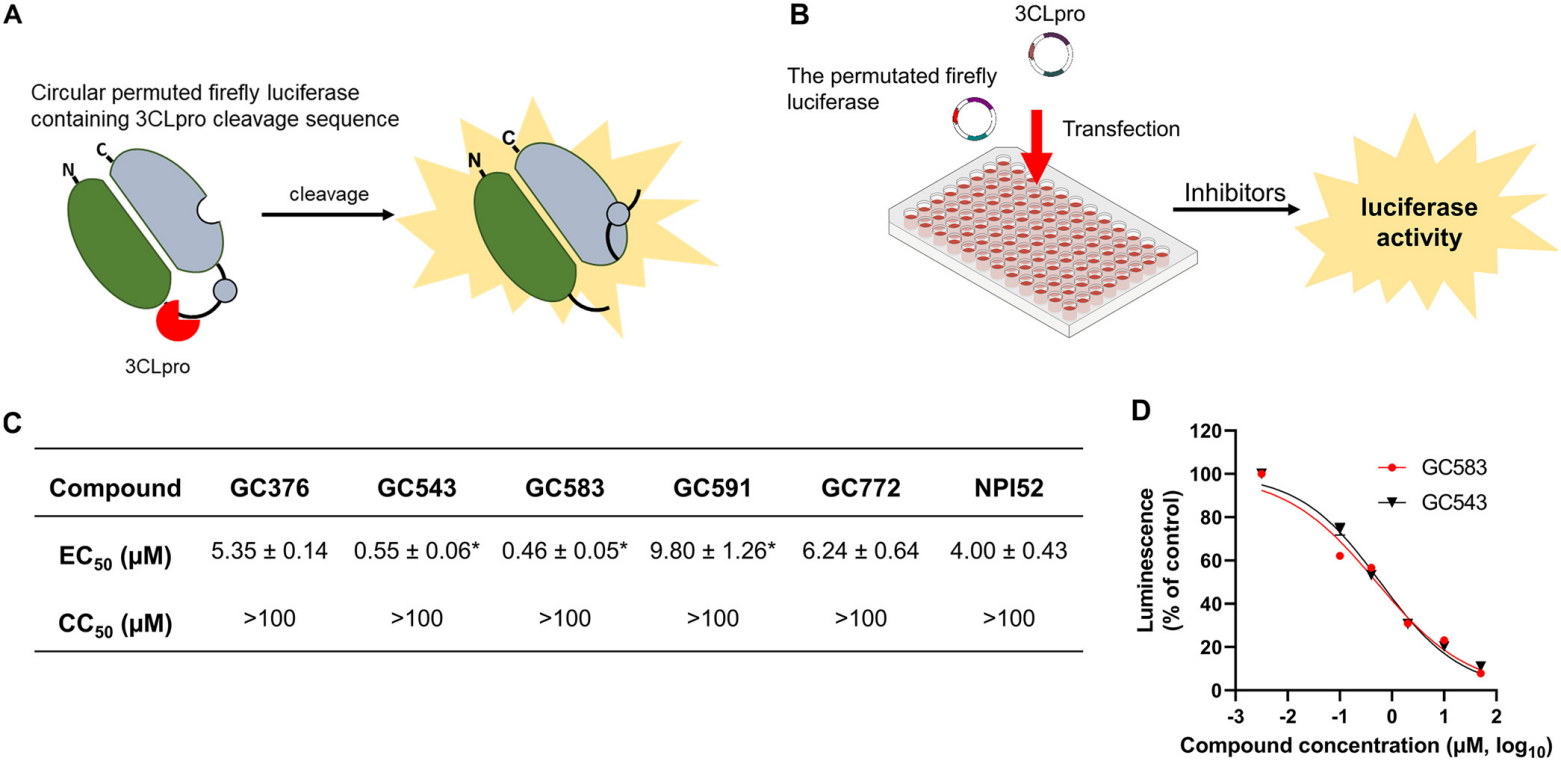
Cell-based reporter assay. (A) A circularly permuted form of firefly luciferase containing an RHDV RHDV 3CLpro cleavage sequence was generated by replacing the caspase 3/7 recognition sequence in the pGloSensor-30F plasmid construct. Cleavage of the 3CLpro recognition sequence restores the activity of firefly luciferase, resulting in an increase in luminescence signal in the presence of luciferase substrate. (B) For inhibition assay, 70 to 80% confluent 293T cells in a 48-well plate were transfected with the plasmids encoding the permuted firefly luciferase and the 3CLpro of GI.3P-GI.2 TX1. After 16 h of incubation of the cells, serially diluted compounds were added to the cells. Then, the cells were further incubated for 5 h before lysis of the cells. Firefly and *Renilla* activity of luciferase activities were measured for the determination of the 50% effective concentration (EC_50_) of each compound. (C) The 3CLpro inhibitory activity in cell-based luciferase reporter assay and cytotoxicity of the compounds. The EC_50_ indicates the 50% effective concentration of a compound against the 3CLpro of GI.3P-GI.2 TX1. The CC_50_ indicates the 50% cytotoxic concentration of a compound. Values are expressed as means ± standard errors of the means from at least three independent experiments. Asterisks indicate statistical significance compared with GC376 (*P* < 0.05). (D) Dose-dependent inhibition curves of GC543 and GC583 based on percent luciferase activity of the 3CLpro in 293T cells. Symbols represent means and the standard errors of the means from at least three independent experiments.

Since lagoviruses do not grow in cell culture, we examined the potency of the compounds against the 3CLpro of GI.3P-GI.2 in the cell-based reporter assay (Fig. 3). In this assay, a circular permutated firefly luciferase containing a cleavage sequence is activated by 3CLpro to generate luminescence (Fig. 3A and B), and inhibition of 3CLpro results in reduced luminescence. The inactive GI.3P-GI.2 3CLpro was included as a control and had minimal luminescence in the assay (<1% of luminescence of active 3CLpro). All of the compounds that were included in the study showed minimal cytotoxicity at 100 μM in 293T cells (Fig. 3C). The 50% effective concentration (EC_50_) values of the compounds ranged from 0.46 to 9.80 μM in 293T cells (Fig. 3C). In this assay system, GC543 and GC583 exhibited most potent activity among the test compounds (*P* < 0.05), which is consistent with the results from the FRET assays (Fig. 2B). GC376 and GC772 showed substantially reduced activity compared to that of GC543 or GC583 (*P* < 0.05) by 9.7- to 11.6-fold, which is comparable to the fold reductions in potency observed in the FRET assay (13.4- to15.1-fold). In the cell-based assay, GC591 showed decreased activity compared to that of GC376 (*P* < 0.05), while its activity was comparable to GC376 in the FRET assay. The dose-dependent curves of GC543 and GC583 against GI.3P-GI.2 3CLpro in the cell-based assay are shown in Fig. 3D.

### Amino acid sequence homology, multiple amino acid sequence alignment, and phylogenetic tree of 3CLpro of lagoviruses.

**FIG 4:**
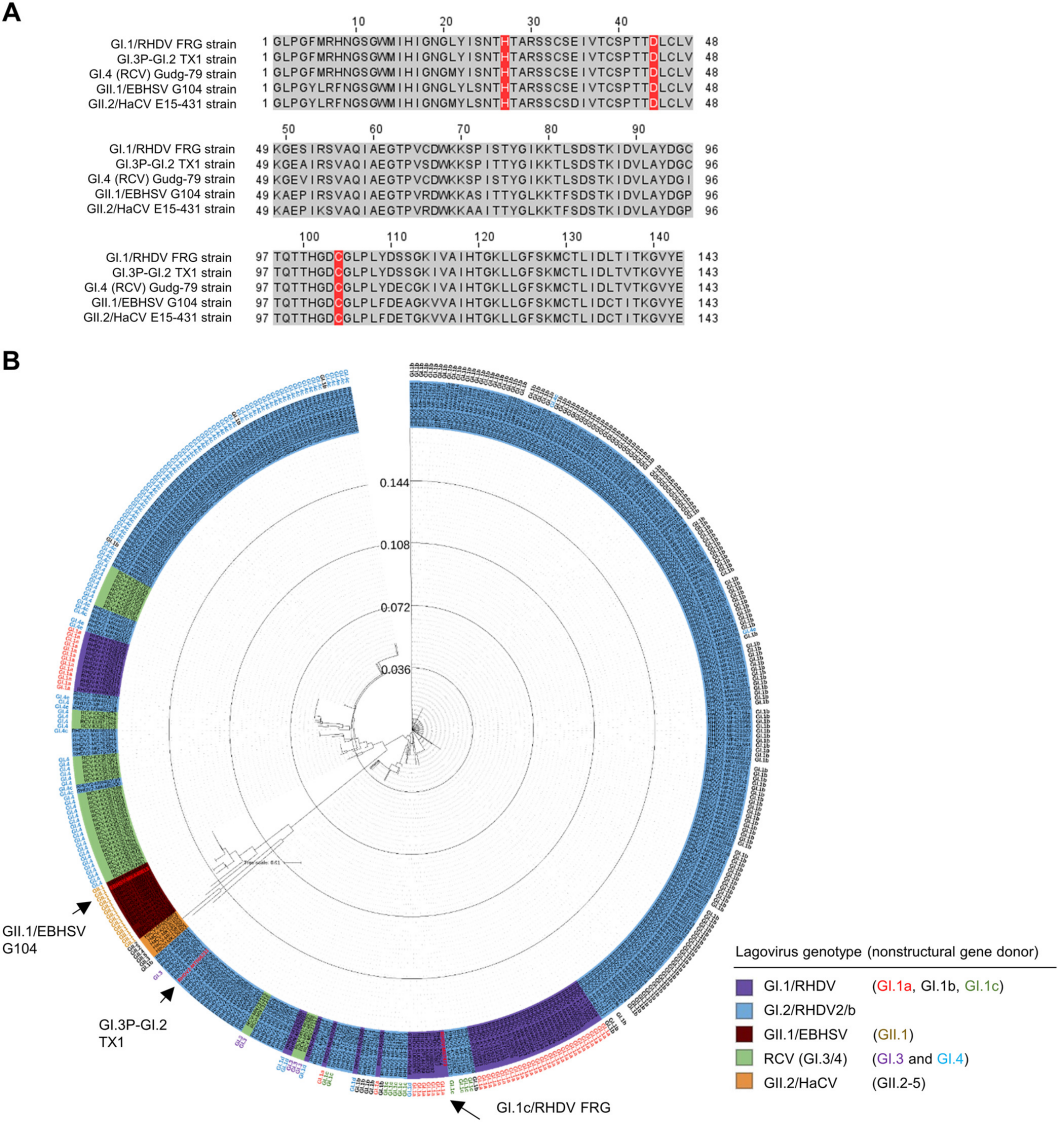
Multiple sequence alignment and phylogenetic analysis of lagovirus 3CLpro. (A) Multiple amino acid sequence alignment of the 3CLpro of the representative strains of GI.1c/RHDV, GI.2/RHDV2/b, RCV (GI.4), GII.1/EBHSV, and GII.2/HaCV. The catalytic residues of 3CLpro (H27, D44, and C104) are highlighted in red. (B) A phylogenetic tree of amino acid sequences of lagovirus 3CLpro, which were retrieved from GenBank, was generated using the neighbor-joining method on the MAFFT web server (https://mafft.cbrc.jp/alignment/server/index.html) and annotated using iTOL v6.4. The 3CLpro used in the screening assay are shown in red and indicated by the arrows. The genotype of the nonstructural protein of each lagovirus, if the information is available in GenBank, is indicated on the outside of the tree. The multiple amino acid alignment of the lagovirus 3CLpro sequences used for generating the tree and the high-resolution figure of the tree are available in the supplemental material.

The multiple amino acid sequence alignment of the representative 3CLpro showed that the catalytic residues H27, D44, and C104 (32) are conserved in the lagovirus 3CLpro (Fig. 4A). The amino acid sequence homology of lagovirus 3CLpro (a total of 454 sequences were selected for alignment) was investigated. The amino acid sequence homology within each virus was high, ranging from 92.31 to 100% for GI.1/RHDV and GI.2/RHDV2/b, 97.90 to 100% for GII.1/EBHSV, 93.71 to 100% for RCV (GI.3 and GI.4), and 86.01 to 100% for GII.2-5/HaCV. The close genetic relationship of 3CLpro shared within GI genogroups was evident with the sequence homology of 92.31 to 100% between GI.1/RHDV and GI.2/RHDV2/b, 93.01 to 100% between RCV and GI.1/RHDV, and between RCV and GI.2/RHDV/b. The 3CLpro sequences of GII.1/EBHSV and GII.2-5/HaCV were closely related to each other with an amino acid sequence homology of 86.01 to 96.50%. However, as expected, the 3CLpro sequences between GI and GII genogroups were less conserved with 82.52 to 88.11% homology. Phylogenetic analysis showed the 3CLpro of GII lagoviruses, GII.1/EBHSV and GII.2-5/HaCV, clustered into the same main branch, while the 3CLpro of GI lagoviruses, GI.1/RHDV, GI.2/RHDV2/b, and RCV, belonged to other branches (Fig. 4B). Interestingly, the clades of 3CLpro of GI lagoviruses are interspersed phylogenetically, which may reflect recombination events that have occurred among these pathogenic and nonpathogenic lagoviruses.

### Three-dimensional homology structural models for 3CLpro.

**FIG 5:**
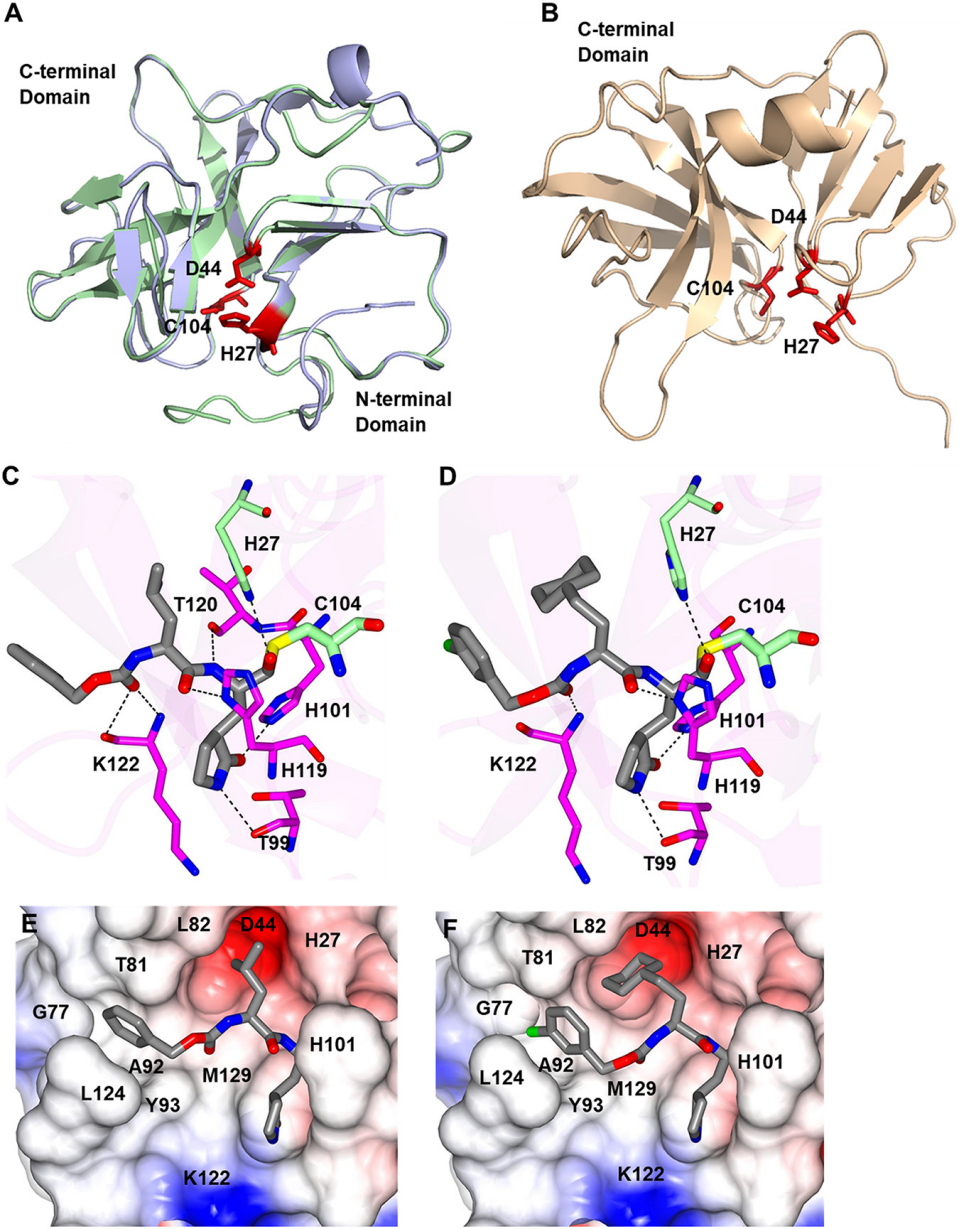
Three-dimensional (3D) homology structural models of GI.1c/RHDV, GI.3P-GI.2 (RHDV2/b), and GII.1/EBHSV 3CLpro (A and B) and molecular interactions between GI.3P-GI.2 3CLpro and docked protease inhibitors (C to F). (A) GI.1c/RHDV FRG 3CLpro (pale green) and GI.3P-GI.2 TX1 3CLpro (pale blue) models are superposed. The catalytic residues (red) overlap each other closely. (B) The 3D model of GII.1/EBHSV G104 3CLpro shows lower structural homology to GI.1c/RHDV and GI.3P-GI.2 3CLpro models. The catalytic residues of GII.1/EBHSV 3CLpro are also shown in red. (C and D) Molecular interactions between GI.3P-GI.2 3CLpro and docked inhibitors (GC376 and GC583). Hydrogen bond interactions between the residues in the active site of the 3CLpro and GC376 (C) or GC583 (D) (gray sticks) are indicated as dashed lines. The catalytic residues of the 3CLpro (C104 and H27) are shown in green. (E and F) The electrostatic surface representations of the 3CLpro bound with GC376 (E) or GC583 (F) are shown with residues colored blue (positive electrostatic potentials), red (negative), or gray (neutral).

The homology-based 3CLpro structural models of GI.1c/RHDV FRG, GI.3P-GI.2 TX1, and GII.2/EBHSV G104 are shown in Fig. 5A and B with the catalytic residues indicated in red. GI.1c/RHDV and GI.3P-GI.2 3CLpro models are very closely homologous (Fig. 5A) with a root mean square deviation (RMSD) of 0.24 Å between the Cα atoms of residues 27 to 104 containing the catalytic residues. However, the 3D model of GII.1/EBHSV 3CLpro (Fig. 5B) showed a lower structural homology with GI.1c/RHDV and GI.3P-GI.2 3CLpro with an RMSD of 9.9 Å between the Cα atoms from residues 27 to 104. High-confidence models of the 3CLpro of GI.3P-GI.2 TX1 were docked with GC376 and GC583 to compare the binding modes. GC376 forms hydrogen bond interactions with H27, T99, H101, H119, T120, and K122 (Fig. 5C). Similarly, GC583 forms hydrogen bond interactions with H27, T99, H101, H119, and K122 (Fig. 5D) but does not interact with T120. The main difference is observed in the P2 position of the compounds, where the cyclohexyl group of GC583 occupies the S2 subsite that is formed by H27, D44, and L82. The benzyl and *m*-chlorobenzyl rings of GC376 and GC583, respectively, are positioned in a hydrophobic cleft in the S4 subsite formed by T81, A92, and L124 (Fig. 5E and F). The S1′, S1, S2, and S4 subsites comprise the substrate-binding cleft of 3CLpro and are the major determinant for inhibitor binding (30). The general nomenclature for the cleavage site positions on substrate and protease subsites is based on the proposal by Schechter and Berger (33). The amino acid residues of a substrate are designated as P3-P2-P1-P1′-P2 from amino to carboxyl terminus, where cleavage occurs between P1-P1′. The S subsites of a protease that bind to the corresponding P residue on the substrate are designated as S3, S2, S1, S1′, S2′, etc.

## DISCUSSION

RHD is a highly contagious viral disease affecting lagomorphs with substantial mortality. However, specific antiviral drugs are not available for RHD and EBHS (34, 35). In subacute/acute forms of RHD, very short duration of disease or sudden death in affected animals severely limits the use of therapeutic agents. However, antiviral drugs may be useful in preventing death or reducing disease severity and duration when used prophylactically in unvaccinated animals and after virus exposure and emergency vaccination before immunity is established, or they may be useful therapeutically for subacute and chronic forms of RHD. Considering the detrimental impact of these virus infections on the global rabbit and hare industry, including important breeding stocks and pet rabbits as well as endangered rabbit and hare species in captivity, development of effective prophylactic and therapeutic treatment would be an important contribution to the limited arsenal of control measures available against these virus diseases.

We have previously generated focused libraries of 3CLpro and 3C protease (3Cpro) inhibitors and reported the *in vitro* and/or *in vivo* potency of some protease inhibitors against caliciviruses (feline calicivirus and human norovirus) (30, 36), coronaviruses (29, 37), and picornaviruses (30). In this study, we evaluated the activity of selected protease inhibitors, including GC376 and its derivatives, against GI.1c/RHDV, GI.3P-GI.2, and GII.1/EBHSV 3CLpro and conducted structure-activity relationship studies using the FRET and cell-based reporter assays. All of the compounds that we tested showed potent activity against the 3CLpro in the FRET and the cell-based reporter assays. GC376 is a dipeptidyl compound that was shown to be broadly effective against various coronaviruses, including feline infectious peritonitis virus (FIPV), a highly virulent feline coronavirus, Middle East respiratory syndrome coronavirus (MERS-CoV), severe acute respiratory syndrome coronavirus 2 (SARS-CoV-2), as well as some caliciviruses and picornaviruses (29, 36–38) and is currently under commercial development as an antiviral drug for FIP. GC376 is a bisulfite adduct prodrug of the corresponding parent aldehyde GC373 (29) and has glutamine surrogate, Leu, and benzyl group in the P1, P2 (R_3_ group), and P3 (R_1_ group) positions, respectively (Table 1). We have previously reported that the antiviral activity of GC376 is slightly more potent against FIPV than aldehyde compounds GC543 and GC583, which have a Cha (cyclohexylalanine) moiety at R_3_ and a benzyl group or *m*-chlorobenzyl group at R_1_, respectively (39). In contrast, GC543 and GC583 have significantly improved activity against all tested lagovirus 3CLpro over GC376, which indicates that the Cha moiety at R_3_ is more suitable for these 3CLpro. The preference of Cha moiety over Leu at R_3_ was also observed for 3CLpro of human norovirus, a calicivirus in the *Norovirus* genus (40) and feline calicivirus that belongs to the *Vesivirus* genus (36). However, substitution of the benzyl group (GC543) with *m*-chlorobenzyl (GC583) at R_1_ did not markedly increase the activity of a compound against the tested lagovirus 3CLpro, unlike human norovirus 3CLpro (40). The results from the cell-based reporter assays that we have established for GI.3P-GI.2 3CLpro correlated well with those determined in the FRET assay except for GC591. The observed disparity in the activity of GC591 in these assay systems may be due to permeability or stability of the compound in the cellular environment. The EC_50_ values from the reporter assay were generally higher than the IC_50_ values from the FRET assay, which is likely due to transient expression of high levels of 3CLpro in cells. One of the protease inhibitors tested, GC376, has an IC_50_ value of 0.5 μM against FIPV 3CLpro in the FRET assay (39) and an EC_50_ value of 0.02 to 0.04 μM against the replication of FIPV in cell culture (29, 36). Considering the *in vivo* efficacy of GC376 in cats with FIP, where reduced viral replication and improved survival were observed (29, 41), all tested compounds, especially GC543 and GC583, seem to have a good potential for further development as a drug for lagovirus infections. GC583 has been previously shown to have *in vivo* efficacy by significantly decreasing murine norovirus replication in the intestinal tracts of mice without toxicity (compound 16 in reference 40).

The multiple sequence alignment analysis and the phylogenetic tree of the amino acid sequences of lagovirus 3CLpro showed that lagovirus 3CLpro, especially GI genotypes, are highly conserved. As expected, the 3CLpro of GI.2/RHDV2/b viruses are interspersed among the 3CLpro of the GI.1 to GI.4 genotypes, indicating GI lagoviruses as the major donors for the 3CLpro of GI.2/RHDV2/b. Notably, despite the sequence homology differences in the 3CLpro of GI.1c/RHDV and GII.1/EBHSV, the 3D homology modeling showed that the catalytic site topology of these 3CLpro appears to be highly conserved, which may explain the comparable potency of compounds against the 3CLpro of GI.1c/RHDV, GI.3P-GI.2, and GII.2/EBHSV. The crystal structures of human norovirus 3CLpro bound to GC376 or GC583 have previously revealed that Cha at the P2 position is filling the hydrophobic S2 subsite of 3CLpro more tightly than Leu (42), which is also predicted in the homology model of the 3CLpro of GI.3P-GI.2 lagovirus. This may explain the observed preference of Cha over Leu at the P2 position of an inhibitor by lagovirus 3CLpro. Overall, our results highlight the similar structural requirements for 3CLpro inhibition of caliciviruses.

In summary, we determined the potency of select protease inhibitors against GI.1c/RHDV, GI.3P-GI.2, and GII.1/EBHSV and conducted structure-activity relationship and 3D homology modeling studies. Using the FRET and the cell-based reporter assays, we identified compounds that display potent inhibitory activities against all three lagovirus 3CLpro. To our knowledge, this is the first report on antiviral compounds that target 3CLpro for development of effective inhibitors broadly active against pathogenic lagoviruses.

## MATERIALS AND METHODS

### Compounds.

The 3CLpro inhibitors, including NPI52 (43), GC376 (30), GC543 (44), GC583 (40), GC571 (40), and GC772 (31), have been previously generated and reported by us. Table 1 contains the list of the compounds used in the study.

### Expression and purification of recombinant lagovirus 3CLpro for FRET assay.

The 3CLpro sequences of GI.1c/RHDV FRG strain, GI.3P-GI.2 TX1 strain, and GII.1/EBHSV G104 strain were obtained from GenBank (accession numbers M67473, MT506233, and MK440613, respectively). The amino acid homology of 3CLpro between GI.1c/RHDV FRG and GI.3P-GI.2 TX1 is 97.2%, and the homology between GI.1/RHDV FRG (or GI.3P-GI.2 TX1) and GII.1/EBHSV G104 is 87.41%. The full-length 3CLpro coding sequences encoding 143 amino acids with N-terminal 6 His tags, which is codon-optimized for protein expression in Escherichia coli, were synthesized by Integrated DNA Technologies (Coralville, IA) and cloned into the pET-28a(+) vector (Addgene, Cambridge, MA). Subsequently, expression of the recombinant 3CLpro in Escherichia coli BL21 cells (Invitrogen, Carlsbad, CA), which were grown in Luria-Bertani broth, was induced with 1 mM isopropyl β-d-thiogalactopyranoside. The recombinant 3CLpro of 16.5 kDa were then purified using HIS GraviTrap Ni-nitrilotriacetic acid (Ni-NTA) affinity columns (GE Healthcare, Chicago, IL) following the standard protocol (30).

### The FRET assay.

First, activities of the recombinant 3CLpro were evaluated. Briefly, serial dilutions of 3CLpro were made in 25 μL of assay buffer (50 mM NaCl, 6 mM dithiothreitol, 50 mM HEPES, 0.4 mM EDTA, and 60% glycerol at pH 8.0) and mixed with the same volume of assay buffer containing the FRET substrate 5-FAM-ASFEGS-K(QXL520)-NH2 (AnaSpec, Fremont, CA). The cleavage site (ASFEGS) of the FRET substrate was derived from the cleavage site between the helicase and VPg of RHDV. The mixture was then added into a black 96-well imaging microplate (Fisher Scientific, Waltham, MA). Following incubation of the plate at room temperature (RT) for up to 90 min, fluorescence readings were measured at excitation and emission values of 485 and 516 nm, respectively, on a fluorescence microplate reader (FL×800; BioTek, Winnooski, VT). The relative fluorescence (activity) of each 3CLpro over time was determined by subtracting the readings of substrate-only control from raw fluorescence readings. After confirming the activity of 3CLpro, the inhibitory activity of the compounds was determined against 3CLpro as previously described (30, 39). Serial dilutions of each compound (10 mM stock solution in dimethyl sulfoxide [DMSO]) were prepared in DMSO or medium, and each dilution was added to 25 μL of assay buffer containing the 3CLpro. The concentration of DMSO in each dilution did not exceed 4% of the final concentration. After incubation at RT for 30 min, the mixture was added into 25 μL of assay buffer containing the FRET substrate in a black 96-well microplate, and the plate was incubated at RT for 30 min. The raw fluorescence readings were measured on the fluorescence microplate reader, and the relative fluorescence was calculated by subtracting the values for substrate-only control from the raw values. The IC_50_ was calculated using the nonlinear regression analysis (four parameter variable slope) in GraphPad Prism software version 6.07 (GraphPad Software, La Jolla, CA).

### Cell-based reporter assay. (i) Generation of plasmids encoding the 3CLpro of GI.3P-GI.2 and circular permutated firefly genes.

The full-length 3CLpro sequence of GI.3P-GI.2 TX1 was codon-optimized for protein expression in mammalian cells, synthesized by Integrated DNA Technologies (Coralville, IA), and inserted into pcDNA3 H2B-mIFP T2A Mpro (3CLpro) (Addgene, Watertown, MA) following digestion with BamHI and ApaI. The resulting plasmid was designated as pcDNA3-RHDV2-3CLpro. A plasmid encoding inactive GI.3P-GI.2 3CLpro was generated by substituting the nucleophilic Cys with Ala in the active site of 3CLpro in pcDNA3-RHDV2-3CLpro using site-directed mutagenesis (Agilent’s QuikChange mutagenesis kit; Agilent, CA) and designated as pcDNA3-RHDV2-m3CLpro. The pGloSensor-30F-DEVDG plasmid (Promega, Madison, WI) contains firefly luciferase gene with a caspase 3/7 cleavage site (DEVDG), which is a circular, permutated form of firefly luciferase (45). To replace the caspase 3/7 cleavage site with a sequence from lagovirus cleavage site (VASFEGAN), site-directed mutagenesis was conducted using primers containing the cleavage sequence. The primer sequences are forward, 5′-GATCCGTGGCCTCATTCGAGGGTGCA-3′, and reverse, 5′-AGCTTGCACCCTCGAATGAGGCCACG-3′. The resulting plasmid was designated as pGlo-RHDV. The plasmid also contains *Renilla* luciferase as an expression control.

### (ii) Assay.

The 293T cells at 70 to 80% confluence in a 48-well plate were cotransfected with pGlo-RHDV and pcDNA3-RHDV2-3CLpro (10 ng of each plasmid per well in a 48-well plate) using Lipofectamine 2000 (Thermo Fisher Scientific, Chicago, IL) at 0.5 μL per well. About 16 h later, medium was replaced with new medium containing serial dilutions (up to 50 μM) of each compound or culture medium, and the cells were incubated at 37°C for 5 h. Then, the cells were lysed and firefly and *Renilla* luminescence were measured following the manufacturer’s direction (Dual-Luciferase kit; Promega, Madison, WI) on a luminometer (GloMax 20/20 luminometer; Promega, Madison, WI). Firefly luciferase was normalized against *Renilla* luciferase, and the EC_50_ of each compound was calculated by GraphPad Prism software using a variable slope (GraphPad, La Jolla, CA). Figure 3A and B illustrates the cell-based reporter assay.

### Cytotoxicity assay.

To determine the cytotoxicity of the compounds, 70 to 80% confluent 293T cells in a 96-well plate were incubated with serial dilutions of each compound (up to 100 μM) for 5 h. Cell cytotoxicity was determined by a CytoTox 96 nonradioactive assay kit (Promega, Madison, WI) by measuring a cytosolic enzyme lactate dehydrogenase, which is released upon cell lysis, following the manufacturer’s instructions, and the 50% cytotoxic concentration (CC_50_) value for each compound was calculated using GraphPad Prism software. The nonspecific cytotoxic effects of these compounds were also reported previously (30, 31, 40, 43, 44).

### Multiple amino acid sequence alignment and generation of phylogenetic tree of lagovirus 3CLpro.

The 3CLpro amino acid sequences of GI.1c/RHDV FRG strain, GI.3P-GI.2 TX1 strain, GII.1/EBHSV G104 strain, RCV (GI.4) Gudg-79 strain, and GII.2/HaCV E15-431 strain were obtained from GenBank (accession numbers M67473, MT506233, MK440613, KX357656, and MH204883, respectively) and aligned using Clustal Omega (https://www.ebi.ac.uk/Tools/msa/clustalo/) (46) to show the conservation of the catalytic residues in lagovirus 3CLpro. To generate a phylogenetic tree, the 3CLpro amino acid sequences of GI.1/RHDV (58 sequences), GI.2/RHDV2/b (333 sequences), GII.1/EBHSV (14 sequences), RCV (G1.3 and GI.4, 44 sequences), and GII.2-5/HaCV (5 sequences) were downloaded using NCBI Mass Sequence Downloader (47) from GenBank. The sequences were aligned, and a phylogenetic tree was generated using the neighbor-joining method on the MAFFT web server (multiple alignment using fast Fourier transform) version 7 (https://mafft.cbrc.jp/alignment/server/) (48). Annotation of phylogenetic tree was carried out using iTOL version 6.4 (49).

### Three-dimensional structural homology modeling of 3CLpro of GI.1c/RHDV, GI.3P-GI.2, and GII.1/EBHSV.

The three-dimensional homology structures of 3CLpro of GI.1c/RHDV FRG strain, GI.3P-GI.2 TX1 strain, and GII.2/EBHSV G104 strain were generated using the Phyre2 web portal (http://www.sbg.bio.ic.ac.uk/phyre2) (50). GI.1c/RHDV and GI.3P-GI.2 3CLpro were modeled using NS6 protease of murine norovirus 1 (Protein Data Bank [PDB] accession number 4ASH) as a template. The templates for modeling GII.2/EBHSV 3CLpro were a hydrolase of E. coli (PDB accession number 2ZLE) and a lyase of Campylobacter jejuni (PDB accession number 6Z05). In general, all of the homology models were generated at >90% confidence for 91 to 100% of residues, and template-modeling scores and RMSD values ranged from 0.31 to 0.62 and 1.86 to 2.56 Å, respectively (https://zhanggroup.org/TM-align/) (51). The constructed 3CLpro models were superposed using the PyMol molecular graphics system version 1.8 (Schrodinger LLC, Cambridge, MA) (52) or SuperPose version 1.0 (http://superpose.wishartlab.com/) (53) to compare the active site conformations. Inhibitor bound models of GI.3P-GI.2 3CLpro with GC376 and GC583 were generated using Alphafold2 (54) and by aligning the binding sites of structures with the highest sequence similarity that included human norovirus 3CLpro bound with GC376 (PDB accession number 3UR9) (30) and GC583 (PDB accession number 4XBB) (40). The ligand bound structure prediction was optimized by superimposing the ligand from the aforementioned crystal structures, adding the covalent bond with the active site cysteine, and running Schrodinger’s protein preparation wizard to optimize hydrogen bonding and minimize the structure into Schrodinger’s OSPL4 energy function (55).

### Statistical analysis.

Data were obtained from at least three independent experiments. Statistical analysis was performed using GraphPad Prism Software version 6 (San Diego, CA). One-way analysis of variance (ANOVA) followed by Tukey *post hoc* test on the IC_50_ and EC_50_ values of compounds were used to identify significant differences between the antiviral activity of the test compounds (*P *< 0.05).

## References

[1] Abrantes J, van der Loo W, Le Pendu J, Esteves PJ. 2012. Rabbit haemorrhagic disease (RHD) and rabbit haemorrhagic disease virus (RHDV): a review. Vet Res 43:12. doi:10.1186/1297-9716-43-12. 22325049 PMC3331820

[2] Gleeson M, Petritz OA. 2020. Emerging infectious diseases of rabbits. Vet Clin North Am Exot Anim Pract 23:249–261. doi:10.1016/j.cvex.2020.01.008. 32327034

[3] Le Pendu J, Abrantes J, Bertagnoli S, Guitton JS, Le Gall-Recule G, Lopes AM, Marchandeau S, Alda F, Almeida T, Celio AP, Barcena J, Burmakina G, Blanco E, Calvete C, Cavadini P, Cooke B, Dalton K, Delibes Mateos M, Deptula W, Eden JS, Wang F, Ferreira CC, Ferreira P, Foronda P, Goncalves D, Gavier-Widen D, Hall R, Hukowska-Szematowicz B, Kerr P, Kovaliski J, Lavazza A, Mahar J, Malogolovkin A, Marques RM, Marques S, Martin-Alonso A, Monterroso P, Moreno S, Mutze G, Neimanis A, Niedzwiedzka-Rystwej P, Peacock D, Parra F, Rocchi M, Rouco C, Ruvoen-Clouet N, Silva E, Silverio D, Strive T, Thompson G, et al. 2017. Proposal for a unified classification system and nomenclature of lagoviruses. J Gen Virol 98:1658–1666. doi:10.1099/jgv.0.000840. 28714849

[4] Liu SJ, Xue HP, Pu BQ, Qian NH. 1984. A new viral disease in rabbits. Anim Husbandry Vet Med 16:253–255.

[5] Le Gall-Recule G, Zwingelstein F, Boucher S, Le Normand B, Plassiart G, Portejoie Y, Decors A, Bertagnoli S, Guerin JL, Marchandeau S. 2011. Detection of a new variant of rabbit haemorrhagic disease virus in France. Vet Rec 168:137–138. doi:10.1136/vr.d697. 21493491

[6] Mahar JE, Hall RN, Peacock D, Kovaliski J, Piper M, Mourant R, Huang N, Campbell S, Gu X, Read A, Urakova N, Cox T, Holmes EC, Strive T. 2018. Rabbit hemorrhagic disease virus 2 (RHDV2; GI.2) is replacing endemic strains of RHDV in the Australian landscape within 18 months of its arrival. J Virol 92:e01374-17. doi:10.1128/JVI.01374-17. PMC5752944 29093089

[7] Rouco C, Abrantes J, Serronha A, Lopes AM, Maio E, Magalhaes MJ, Blanco E, Barcena J, Esteves PJ, Santos N, Alves PC, Monterroso P. 2018. Epidemiology of RHDV2 (Lagovirus europaeus/GI.2) in free-living wild European rabbits in Portugal. Transbound Emerg Dis 65:e373–e382. doi:10.1111/tbed.12767. 29150915

[8] Neimanis AS, Ahola H, Zohari S, Larsson Pettersson U, Brojer C, Capucci L, Gavier-Widen D. 2018. Arrival of rabbit haemorrhagic disease virus 2 to northern Europe: emergence and outbreaks in wild and domestic rabbits (Oryctolagus cuniculus) in Sweden. Transbound Emerg Dis 65:213–220. doi:10.1111/tbed.12650. 28407381

[9] United States Department of Agriculture. 2020. Factsheet - rabbit hemorrhagic disease. United States Department of Agriculture, Washington, DC. https://www.aphis.usda.gov/publications/animal_health/fs-rhdv2.pdf. Accessed 14 April 2022.

[10] Florida Department of Agriculture and Consumer Services. 2022. Rabbit hemorrhagic disease update. Florida Department of Agriculture and Consumer Services, Tallahassee, FL. https://www.fdacs.gov/content/download/98220/file/rabbit-hemorrhagic-disease-update-3172022.pdf. Accessed 14 April 2022.

[11] OIE. 2018. Chapter 3.6.2. Rabbit haemorrhagic disease. OIE, Paris, France. https://www.oie.int/fileadmin/Home/eng/Health_standards/tahm/3.06.02_RHD.pdf. Accessed 25 February 2022.

[12] Le Gall-Recule G, Lemaitre E, Bertagnoli S, Hubert C, Top S, Decors A, Marchandeau S, Guitton JS. 2017. Large-scale lagovirus disease outbreaks in European brown hares (Lepus europaeus) in France caused by RHDV2 strains spatially shared with rabbits (Oryctolagus cuniculus). Vet Res 48:70. doi:10.1186/s13567-017-0473-y. 29080562 PMC5660455

[13] Velarde R, Cavadini P, Neimanis A, Cabezon O, Chiari M, Gaffuri A, Lavin S, Grilli G, Gavier-Widen D, Lavazza A, Capucci L. 2017. Spillover events of infection of brown hares (Lepus europaeus) with rabbit haemorrhagic disease type 2 virus (RHDV2) caused sporadic cases of an European brown hare syndrome-like disease in Italy and Spain. Transbound Emerg Dis 64:1750–1761. doi:10.1111/tbed.12562. 27615998 PMC5697611

[14] Buehler M, Jesse ST, Kueck H, Lange B, Koenig P, Jo WK, Osterhaus A, Beineke A. 2020. Lagovirus europeus GI.2 (rabbit hemorrhagic disease virus 2) infection in captive mountain hares (Lepus timidus) in Germany. BMC Vet Res 16:166. doi:10.1186/s12917-020-02386-4. 32460756 PMC7254734

[15] Dalton KP, Nicieza I, Balseiro A, Muguerza MA, Rosell JM, Casais R, Alvarez AL, Parra F. 2012. Variant rabbit hemorrhagic disease virus in young rabbits, Spain. Emerg Infect Dis 18:2009–2012. doi:10.3201/eid1812.120341. 23171812 PMC3557890

[16] United States Department of Agriculture. 2020. Emerging risk notice. United States Department of Agriculture, Washington, DC. https://www.aphis.usda.gov/animal_health/downloads/rhdv2.pdf. Accessed 14 April 2022.

[17] Villafuerte R, Delibes-Mateos M. 2019. Oryctolagus cuniculus. The IUCN Red List of Threatened Species 2019: e.T41291A45189779. doi:10.2305/IUCN.UK.2019-3.RLTS.T41291A45189779.en. Accessed 17 December 2021.

[18] Frolich K, Haerer G, Bacciarini L, Janovsky M, Rudolph M, Giacometti M. 2001. European brown hare syndrome in free-ranging European brown and mountain hares from Switzerland. J Wildl Dis 37:803–807. doi:10.7589/0090-3558-37.4.803. 11763744

[19] Billinis C, Psychas V, Tontis DK, Spyrou V, Birtsas PK, Sofia M, Likotrafitis F, Maslarinou OM, Kanteres D. 2005. European brown hare syndrome in wild European brown hares from Greece. J Wildl Dis 41:783–786. doi:10.7589/0090-3558-41.4.783. 16456168

[20] Guberti V, De Marco A, Riga F, Lavazza A, Trocchi V, Capucci L. 2000. Virology and species conservation: the case of EBHSV and the Italian hare. Lepus Corsicanus 198:199.

[21] Gavier-Widen D, Morner T. 1993. Descriptive epizootiological study of European brown hare syndrome in Sweden. J Wildl Dis 29:15–20. doi:10.7589/0090-3558-29.1.15. 8383252

[22] OIE. 2020. European brown hare syndrome virus. https://www.oie.int/app/uploads/2021/05/european-brown-hare-syndrome-virus-infection-with.pdf. OIE, Paris, France. Accessed 25 February 2022.

[23] Szillat KP, Hoper D, Beer M, Konig P. 2020. Full-genome sequencing of German rabbit haemorrhagic disease virus uncovers recombination between RHDV (GI.2) and EBHSV (GII.1). Virus Evol 6:veaa080. doi:10.1093/ve/veaa080. 33324492 PMC7724246

[24] Abrantes J, Droillard C, Lopes AM, Lemaitre E, Lucas P, Blanchard Y, Marchandeau S, Esteves PJ, Le Gall-Recule G. 2020. Recombination at the emergence of the pathogenic rabbit haemorrhagic disease virus Lagovirus europaeus/GI.2. Sci Rep 10:14502. doi:10.1038/s41598-020-71303-4. 32879332 PMC7468141

[25] Mahar JE, Jenckel M, Huang N, Smertina E, Holmes EC, Strive T, Hall RN. 2021. Frequent intergenotypic recombination between the non-structural and structural genes is a major driver of epidemiological fitness in caliciviruses. Virus Evol 7:veab080. doi:10.1093/ve/veab080. 34754513 PMC8570162

[26] Matthaei M, Kerr PJ, Read AJ, Hick P, Haboury S, Wright JD, Strive T. 2014. Comparative quantitative monitoring of rabbit haemorrhagic disease viruses in rabbit kittens. Virol J 11:109. doi:10.1186/1743-422X-11-109. 24913134 PMC4060863

[27] Henning J, Meers J, Davies PR, Morris RS. 2005. Survival of rabbit haemorrhagic disease virus (RHDV) in the environment. Epidemiol Infect 133:719–730. doi:10.1017/s0950268805003766. 16050519 PMC2870301

[28] McColl KA, Merchant JC, Hardy J, Cooke BD, Robinson A, Westbury HA. 2002. Evidence for insect transmission of rabbit haemorrhagic disease virus. Epidemiol Infect 129:655–663. doi:10.1017/s0950268802007756. 12558351 PMC2869930

[29] Kim Y, Liu H, Galasiti Kankanamalage AC, Weerasekara S, Hua DH, Groutas WC, Chang KO, Pedersen NC. 2016. Reversal of the progression of fatal coronavirus infection in cats by a broad-spectrum coronavirus protease inhibitor. PLoS Pathog 12:e1005531. doi:10.1371/journal.ppat.1005531. 27027316 PMC4814111

[30] Kim Y, Lovell S, Tiew K-C, Mandadapu SR, Alliston KR, Battaile KP, Groutas WC, Chang K-O. 2012. Broad-spectrum antivirals against 3C or 3C-like proteases of picornaviruses, noroviruses, and coronaviruses. J Virol 86:11754–11762. doi:10.1128/JVI.01348-12. 22915796 PMC3486288

[31] Galasiti Kankanamalage AC, Kim Y, Rathnayake AD, Damalanka VC, Weerawarna PM, Doyle ST, Alsoudi AF, Dissanayake DMP, Lushington GH, Mehzabeen N, Battaile KP, Lovell S, Chang KO, Groutas WC. 2017. Structure-based exploration and exploitation of the S4 subsite of norovirus 3CL protease in the design of potent and permeable inhibitors. Eur J Med Chem 126:502–516. doi:10.1016/j.ejmech.2016.11.027. 27914364 PMC5501333

[32] Oka T, Murakami K, Wakita T, Katayama K. 2011. Comparative site-directed mutagenesis in the catalytic amino acid triad in calicivirus proteases. Microbiol Immunol 55:108–114. doi:10.1111/j.1348-0421.2010.00295.x. 21204947

[33] Schechter I, Berger A. 1967. On the size of the active site in proteases. I. Papain. Biochem Biophys Res Commun 27:157–162. doi:10.1016/s0006-291x(67)80055-x. 6035483

[34] Du H, Zhang S, He M, Ming K, Wang J, Yuan W, Qiao M, Wu Y, Wang D, Hu Y, Liu J. 2019. Evaluation of the therapeutic effect of a flavonoid prescription against rabbit hemorrhagic disease in vivo. Biomed Res Int 2019:5201790. doi:10.1155/2019/5201790. 31080820 PMC6475574

[35] Urakova N, Netzler N, Kelly AG, Frese M, White PA, Strive T. 2016. Purification and biochemical characterisation of rabbit calicivirus RNA-dependent RNA polymerases and identification of non-nucleoside inhibitors. Viruses 8:100. doi:10.3390/v8040100. 27089358 PMC4848594

[36] Kim Y, Shivanna V, Narayanan S, Prior AM, Weerasekara S, Hua DH, Kankanamalage AC, Groutas WC, Chang KO. 2015. Broad-spectrum inhibitors against 3C-like proteases of feline coronaviruses and feline caliciviruses. J Virol 89:4942–4950. doi:10.1128/JVI.03688-14. 25694593 PMC4403489

[37] Rathnayake AD, Zheng J, Kim Y, Perera KD, Mackin S, Meyerholz DK, Kashipathy MM, Battaile KP, Lovell S, Perlman S, Groutas WC, Chang KO. 2020. 3C-like protease inhibitors block coronavirus replication in vitro and improve survival in MERS-CoV-infected mice. Sci Transl Med 12:eabc5332. doi:10.1126/scitranslmed.abc5332. 32747425 PMC7574915

[38] Dampalla CS, Zheng J, Perera KD, Wong LR, Meyerholz DK, Nguyen HN, Kashipathy MM, Battaile KP, Lovell S, Kim Y, Perlman S, Groutas WC, Chang KO. 2021. Postinfection treatment with a protease inhibitor increases survival of mice with a fatal SARS-CoV-2 infection. Proc Natl Acad Sci USA 118:e2101555118. doi:10.1073/pnas.2101555118. 34210738 PMC8307543

[39] Perera KD, Galasiti Kankanamalage AC, Rathnayake AD, Honeyfield A, Groutas W, Chang KO, Kim Y. 2018. Protease inhibitors broadly effective against feline, ferret and mink coronaviruses. Antiviral Res 160:79–86. doi:10.1016/j.antiviral.2018.10.015. 30342822 PMC6240502

[40] Galasiti Kankanamalage AC, Kim Y, Weerawarna PM, Uy RA, Damalanka VC, Mandadapu SR, Alliston KR, Mehzabeen N, Battaile KP, Lovell S, Chang KO, Groutas WC. 2015. Structure-guided design and optimization of dipeptidyl inhibitors of norovirus 3CL protease. Structure-activity relationships and biochemical, X-ray crystallographic, cell-based, and in vivo studies. J Med Chem 58:3144–3155. doi:10.1021/jm5019934. 25761614 PMC4484267

[41] Pedersen NC, Kim Y, Liu H, Galasiti Kankanamalage AC, Eckstrand C, Groutas WC, Bannasch M, Meadows JM, Chang KO. 2018. Efficacy of a 3C-like protease inhibitor in treating various forms of acquired feline infectious peritonitis. J Feline Med Surg 20:378–392. doi:10.1177/1098612X17729626. 28901812 PMC5871025

[42] Chang K-O, Kim Y, Lovell S, Rathnayake AD, Groutas WC. 2019. Antiviral drug discovery: norovirus proteases and development of inhibitors. Viruses 11:197. doi:10.3390/v11020197. 30823509 PMC6410195

[43] Prior AM, Kim Y, Weerasekara S, Moroze M, Alliston KR, Uy RA, Groutas WC, Chang KO, Hua DH. 2013. Design, synthesis, and bioevaluation of viral 3C and 3C-like protease inhibitors. Bioorg Med Chem Lett 23:6317–6320. doi:10.1016/j.bmcl.2013.09.070. 24125888 PMC3863581

[44] Mandadapu SR, Gunnam MR, Tiew KC, Uy RA, Prior AM, Alliston KR, Hua DH, Kim Y, Chang KO, Groutas WC. 2013. Inhibition of norovirus 3CL protease by bisulfite adducts of transition state inhibitors. Bioorg Med Chem Lett 23:62–65. doi:10.1016/j.bmcl.2012.11.026. 23218713 PMC3586229

[45] Galban S, Jeon YH, Bowman BM, Stevenson J, Sebolt KA, Sharkey LM, Lafferty M, Hoff BA, Butler BL, Wigdal SS, Binkowski BF, Otto P, Zimmerman K, Vidugiris G, Encell LP, Fan F, Wood KV, Galban CJ, Ross BD, Rehemtulla A. 2013. Imaging proteolytic activity in live cells and animal models. PLoS One 8:e66248. doi:10.1371/journal.pone.0066248. 23776643 PMC3679058

[46] Madeira F, Park YM, Lee J, Buso N, Gur T, Madhusoodanan N, Basutkar P, Tivey ARN, Potter SC, Finn RD, Lopez R. 2019. The EMBL-EBI search and sequence analysis tools APIs in 2019. Nucleic Acids Res 47:W636–W641. doi:10.1093/nar/gkz268. 30976793 PMC6602479

[47] Pina-Martins F, Paulo OS. 2016. NCBI Mass Sequence Downloader–large dataset downloading made easy. SoftwareX 5:80–83. doi:10.1016/j.softx.2016.04.007.

[48] Rozewicki J, Li S, Amada KM, Standley DM, Katoh K. 2019. MAFFT-DASH: integrated protein sequence and structural alignment. Nucleic Acids Res 47:W5–W10. doi:10.1093/nar/gkz342. 31062021 PMC6602451

[49] Letunic I, Bork P. 2021. Interactive Tree Of Life (iTOL) v5: an online tool for phylogenetic tree display and annotation. Nucleic Acids Res 49:W293–W296. doi:10.1093/nar/gkab301. 33885785 PMC8265157

[50] Kelley LA, Mezulis S, Yates CM, Wass MN, Sternberg MJ. 2015. The Phyre2 web portal for protein modeling, prediction and analysis. Nat Protoc 10:845–858. doi:10.1038/nprot.2015.053. 25950237 PMC5298202

[51] Zhang Y, Skolnick J. 2005. TM-align: a protein structure alignment algorithm based on the TM-score. Nucleic Acids Res 33:2302–2309. doi:10.1093/nar/gki524. 15849316 PMC1084323

[52] DeLano WL. 2010. The PyMOL molecular graphics system, *on* DeLano Scientific LLC. http://www.pymol.org. Accessed 17 December 2021.

[53] Maiti R, Zhang H, Wishart D. 2004. SuperPose version 1.0 http://superpose.wishartlab.com/. Accessed 17 December 2021.

[54] Jumper J, Evans R, Pritzel A, Green T, Figurnov M, Ronneberger O, Tunyasuvunakool K, Bates R, Zidek A, Potapenko A, Bridgland A, Meyer C, Kohl SAA, Ballard AJ, Cowie A, Romera-Paredes B, Nikolov S, Jain R, Adler J, Back T, Petersen S, Reiman D, Clancy E, Zielinski M, Steinegger M, Pacholska M, Berghammer T, Bodenstein S, Silver D, Vinyals O, Senior AW, Kavukcuoglu K, Kohli P, Hassabis D. 2021. Highly accurate protein structure prediction with AlphaFold. Nature 596:583–589. doi:10.1038/s41586-021-03819-2. 34265844 PMC8371605

[55] Schrödinger LLC. 2019. Schrödinger release 2019–4: protein preparation wizard; Epik; impact; prime; glide; LigPrep; induced fit docking protocol. Schrödinger, New York, NY.

