# Supplementary material for: Potent Protease Inhibitors of Highly Pathogenic Lagoviruses: Rabbit Hemorrhagic Disease Virus and European Brown Hare Syndrome Virus
Source: Microbiol Spectr. 2022 Jun 29;10(4):e00142-22. doi: 10.1128/spectrum.00142-22 (PMC9430360; doi:10.1128/spectrum.00142-22)

Supplemental File S1. Multiple amino acid sequence alignment of lagovirus 3CLpro by MAFFT (Multiple Alignment using Fast Fourier Transform) (v7.504).

```
RHDV2-MN901451  GLPGFMRHNGSGWMIHIGNGLYISNTHTARSSCSEIVTCSPTTDLCLVKGEAIRSVAQIA
RHDV2-MN061492  GLPGFMRHNGSGWMIHIGNGLYISNTHTARSSCSEIVTCSPTTDLCLVKGEAIRSVAQIA
RHDV2-MN738377  GLPGFMRHNGSGWMIHIGNGLYISNTHTARSSCSEIVTCSPTTDLCLVKGEAIRSVAQIA
RHDV2-MN786321  GLPGFMRHNGSGWMIHIGNGLYISNTHTARSSCSEIVTCSPTTDLCLVKGEAIRSVAQIA
RHDV2-MT506233  GLPGFMRHNGSGWMIHIGNGLYISNTHTARSSCSEIVTCSPTTDLCLVKGEAIRSVAQIA
RHDV2-MT506234  GLPGFMRHNGSGWMIHIGNGLYISNTHTARSSCSEIVTCSPTTDLCLVKGEAIRSVAQIA
RHDV2-MT506235  GLPGFMRHNGSGWMIHIGNGLYISNTHTARSSCSEIVTCSPTTDLCLVKGEAIRSVAQIA
RHDV2-MT506236  GLPGFMRHNGSGWMIHIGNGLYISNTHTARSSCSEIVTCSPTTDLCLVKGEAIRSVAQIA
RHDV2-MW926372  GLPGFMRHNGSGWMIHIGNGLYISNTHTARSSCSEIVTCSPTTDLCLVKGEAIRSVAQIA
RHDV2-MW926373  GLPGFMRHNGSGWMIHIGNGLYISNTHTARSSCSEIVTCSPTTDLCLVKGEAIRSVAQIA
RHDV2-MW926374  GLPGFMRHNGSGWMIHIGNGLYISNTHTARSSCSEIVTCSPTTDLCLVKGEAIRSVAQIA
RHDV2-MW926375  GLPGFMRHNGSGWMIHIGNGLYISNTHTARSSCSEIVTCSPTTDLCLVKGEAIRSVAQIA
RHDV2-MW926376  GLPGFMRHNGSGWMIHIGNGLYISNTHTARSSCSEIVTCSPTTDLCLVKGEAIRSVAQIA
RHDV2-MW926377  GLPGFMRHNGSGWMIHIGNGLYISNTHTARSSCSEIVTCSPTTDLCLVKGEAIRSVAQIA
RHDV2-MW926379  GLPGFMRHNGSGWMIHIGNGLYISNTHTARSSCSEIVTCSPTTDLCLVKGEAIRSVAQIA
RHDV2-MW926380  GLPGFMRHNGSGWMIHIGNGLYISNTHTARSSCSEIVTCSPTTDLCLVKGEAIRSVAQIA
RHDV2-MW926382  GLPGFMRHNGSGWMIHIGNGLYISNTHTARSSCSEIVTCSPTTDLCLVKGEAIRSVAQIA
RHDV2-MW926383  GLPGFMRHNGSGWMIHIGNGLYISNTHTARSSCSEIVTCSPTTDLCLVKGEAIRSVAQIA
RHDV2-MW926384  GLPGFMRHNGSGWMIHIGNGLYISNTHTARSSCSEIVTCSPTTDLCLVKGEAIRSVAQIA
RHDV2-MW926371  GLPGFMRHNGSGWMIHIGNGLYISNTHTARSSCSEIVTCSPTTDLCLVKGEAIRSVAQIA
RHDV2-MN853660  GLPGFMRHNGSGWMIHIGNGLYISNTHTARSSCSEIVTCSPTTDLCLVKGEAIRSVAQIA
RHDV2-MN853661  GLPGFMRHNGSGWMIHIGNGLYISNTHTARSSCSEIVTCSPTTDLCLVKGEAIRSVAQIA
RHDV2-MW926381  GLPGFMRHNGSGWMIHIGNGLYISNTHTARSSCSEIVTCSPTTDLCLVKGEAIRSVAQIA
RHDV2-MN853659  GLPGFMRHNGSGWMIHIGNGLYISNTHTARSSCSEIVTCSPTTDLCLVKGEAIRSVAQIA
RHDV2-MW926378  GLPGFMRHNGSGWMIHIGNGLYISNTHTARSSCSEIVTCSPTTDLCLVKGEAIRSVAQIA
RHDV2-MG763942  GLPGFMRHNGSGWMIHIGNGLYISNTHTARSSCSEIVTCSPTTDLCLVKGETIRSVAQIA
RHDV2-MG763943  GLPGFMRHNGSGWMIHIGNGLYISNTHTARSSCSEIVTCSPTTDLCLVKGETIRSVAQIA
RHDV2-MG763945  GLPGFMRHNGSGWMIHIGNGLYISNTHTARSSCSEIVTCSPTTDLCLVKGETIRSVAQIA
RHDV2-MG763949  GLPGFMRHNGSGWMIHIGNGLYISNTHTARSSCSEIVTCSPTTDLCLVKGETIRSVAQIA
RHDV2-MG763950  GLPGFMRHNGSGWMIHIGNGLYISNTHTARSSCSEIVTCSPTTDLCLVKGETIRSVAQIA
RHDV2-MG763951  GLPGFMRHNGSGWMIHIGNGLYISNTHTARSSCSEIVTCSPTTDLCLVKGETIRSVAQIA
RHDV2-MF407653  GLPGFMRHNGSGWMIHIGNGLYISNTHTARSSCSEIVTCSPTTDLCLVKGETIRSVAQIA
RHDV2-MN737113  GLPGFMRHNGSGWMIHIGNGLYISNTHTARSSCSEIVTCSPTTDLCLVKGETIRSVAQIA
RHDV-MT628288   GLPGFMRHNGSGWMIHIGNGLYISNTHTARSSCSEIVTCSPTTDLCLVKGETIRSVAQIA
RHDV-MT628291   GLPGFMRHNGSGWMIHIGNGLYISNTHTARSSCSEIVTCSPTTDLCLVKGETIRSVAQIA
RCV-MN746289    GLPGFMRHNGSGWMIHIGNGLYISNTHTARSSCSEIVTCSPTTDLCLVKGETIRSVAQIA
RCV-MN746288    GLPGFMRHNGSGWMIHIGNGLYISNTHTARSSCSEIVTCSPTTDLCLVKGETIRSVAQIA
RCV-MN737117    GLPGFMRHNGSGWMIHIGNGLYISNTHTARSSCSEIVTCSPTTDLCLVKGETIRSVAQIA
RHDV2-MN737114  GLPGFMRHNGSGWMIHIGNGLYISNTHTARSSCSEIVTCSPTTDLCLVKGEVIRSVAQIA
RHDV2-MW974834  GLPGFMRHNGSGWMIHIGNGLYISNTHTARSSCSEIVTCSPTTDLCLVKGEVIRSVAQIA
```

```
RHDV2-MT586027  GLPGFMRHNGSGWMIHIGNGLYISNTHTARSSCSEIVTCSPTTDLCLVKGEVIRSVAQIA
RHDV-MT628290   GLPGFMRHNGSGWMIHIGNGLYISNTHTARSSCSEIVTCSPTTDLCLVKGEIIRSVAQIA
RCV-MN737115    GLPGFMRHNGSGWMIHIGNGLYISNTHTARSSCSEIVTCSPTTDLCLVKGETIRSVAQIA
RCV-MN737116    GLPGFMRHNGSGWMIHIGNGLYISNTHTARSSCSEIVTCSPTTDLCLVKGETIRSVAQIA
RHDV2-MK521927  GLPGFMRHNGSGWMIHIGNGLYISNTHTARSSCSEIVTCSPTTDLCLVKGEVIRSVAQIA
RHDV2-MG602006  GLPGFMRHNGSGWMIHIGNGLYISNTHTARSSCSEIVTCSPTTDLCLVKGEVIRSVAQIA
RHDV2-MG602007  GLPGFMRHNGSGWMIHIGNGLYISNTHTARSSCSEIVTCSPTTDLCLVKGEVIRSVAQIA
RHDV2-MG763937  GLPGFMRHNGSGWMIHIGNGLYISNTHTARSSCSEIVTCSPTTDLCLVKGEVIRSVAQIA
RHDV2-MG763938  GLPGFMRHNGSGWMIHIGNGLYISNTHTARSSCSEIVTCSPTTDLCLVKGEVIRSVAQIA
RHDV2-MG763939  GLPGFMRHNGSGWMIHIGNGLYISNTHTARSSCSEIVTCSPTTDLCLVKGEVIRSVAQIA
RHDV2-MG763940  GLPGFMRHNGSGWMIHIGNGLYISNTHTARSSCSEIVTCSPTTDLCLVKGEVIRSVAQIA
RHDV2-MG763948  GLPGFMRHNGSGWMIHIGNGLYISNTHTARSSCSEIVTCSPTTDLCLVKGEVIRSVAQIA
RHDV2-MG763953  GLPGFMRHNGSGWMIHIGNGLYISNTHTARSSCSEIVTCSPTTDLCLVKGEVIRSVAQIA
RHDV2-MF407654  GLPGFMRHNGSGWMIHIGNGLYISNTHTARSSCSEIVTCSPTTDLCLVKGEVIRSVAQIA
RHDV2-MF407656  GLPGFMRHNGSGWMIHIGNGLYISNTHTARSSCSEIVTCSPTTDLCLVKGEVIRSVAQIA
RHDV2-MW460036  GLPGFMRHNGSGWMIHIGNGLYISNTHTARSSCSEIVTCSPTTDLCLVKGEVIRSVAQIA
RHDV2-MW460101  GLPGFMRHNGSGWMIHIGNGLYISNTHTARSSCSEIVTCSPTTDLCLVKGEVIRSVAQIA
RHDV2-MW460102  GLPGFMRHNGSGWMIHIGNGLYISNTHTARSSCSEIVTCSPTTDLCLVKGEVIRSVAQIA
RHDV2-MW460103  GLPGFMRHNGSGWMIHIGNGLYISNTHTARSSCSEIVTCSPTTDLCLVKGEVIRSVAQIA
RHDV2-MW460105  GLPGFMRHNGSGWMIHIGNGLYISNTHTARSSCSEIVTCSPTTDLCLVKGEVIRSVAQIA
RHDV2-MW460106  GLPGFMRHNGSGWMIHIGNGLYISNTHTARSSCSEIVTCSPTTDLCLVKGEVIRSVAQIA
RHDV2-MW460112  GLPGFMRHNGSGWMIHIGNGLYISNTHTARSSCSEIVTCSPTTDLCLVKGEVIRSVAQIA
RHDV2-MW460113  GLPGFMRHNGSGWMIHIGNGLYISNTHTARSSCSEIVTCSPTTDLCLVKGEVIRSVAQIA
RHDV2-MW460114  GLPGFMRHNGSGWMIHIGNGLYISNTHTARSSCSEIVTCSPTTDLCLVKGEVIRSVAQIA
RHDV2-MW460115  GLPGFMRHNGSGWMIHIGNGLYISNTHTARSSCSEIVTCSPTTDLCLVKGEVIRSVAQIA
RHDV2-MW460116  GLPGFMRHNGSGWMIHIGNGLYISNTHTARSSCSEIVTCSPTTDLCLVKGEVIRSVAQIA
RHDV2-MW460126  GLPGFMRHNGSGWMIHIGNGLYISNTHTARSSCSEIVTCSPTTDLCLVKGEVIRSVAQIA
RHDV2-MW460138  GLPGFMRHNGSGWMIHIGNGLYISNTHTARSSCSEIVTCSPTTDLCLVKGEVIRSVAQIA
RHDV2-MW460145  GLPGFMRHNGSGWMIHIGNGLYISNTHTARSSCSEIVTCSPTTDLCLVKGEVIRSVAQIA
RHDV2-MW460162  GLPGFMRHNGSGWMIHIGNGLYISNTHTARSSCSEIVTCSPTTDLCLVKGEVIRSVAQIA
RHDV2-MW460172  GLPGFMRHNGSGWMIHIGNGLYISNTHTARSSCSEIVTCSPTTDLCLVKGEVIRSVAQIA
RHDV2-MW460176  GLPGFMRHNGSGWMIHIGNGLYISNTHTARSSCSEIVTCSPTTDLCLVKGEVIRSVAQIA
RHDV2-MW460182  GLPGFMRHNGSGWMIHIGNGLYISNTHTARSSCSEIVTCSPTTDLCLVKGEVIRSVAQIA
RHDV2-MW460188  GLPGFMRHNGSGWMIHIGNGLYISNTHTARSSCSEIVTCSPTTDLCLVKGEVIRSVAQIA
RHDV2-MW460189  GLPGFMRHNGSGWMIHIGNGLYISNTHTARSSCSEIVTCSPTTDLCLVKGEVIRSVAQIA
RHDV2-MW460191  GLPGFMRHNGSGWMIHIGNGLYISNTHTARSSCSEIVTCSPTTDLCLVKGEVIRSVAQIA
RHDV2-MW460203  GLPGFMRHNGSGWMIHIGNGLYISNTHTARSSCSEIVTCSPTTDLCLVKGEVIRSVAQIA
RHDV2-MW460205  GLPGFMRHNGSGWMIHIGNGLYISNTHTARSSCSEIVTCSPTTDLCLVKGEVIRSVAQIA
RHDV2-MW460206  GLPGFMRHNGSGWMIHIGNGLYISNTHTARSSCSEIVTCSPTTDLCLVKGEVIRSVAQIA
RHDV2-MW460207  GLPGFMRHNGSGWMIHIGNGLYISNTHTARSSCSEIVTCSPTTDLCLVKGEVIRSVAQIA
RHDV2-MW460215  GLPGFMRHNGSGWMIHIGNGLYISNTHTARSSCSEIVTCSPTTDLCLVKGEVIRSVAQIA
RHDV2-MW460221  GLPGFMRHNGSGWMIHIGNGLYISNTHTARSSCSEIVTCSPTTDLCLVKGEVIRSVAQIA
RHDV2-MW460222  GLPGFMRHNGSGWMIHIGNGLYISNTHTARSSCSEIVTCSPTTDLCLVKGEVIRSVAQIA
RHDV2-MW460225  GLPGFMRHNGSGWMIHIGNGLYISNTHTARSSCSEIVTCSPTTDLCLVKGEVIRSVAQIA
```

```
RHDV2-MW467791  GLPGFMRHNGSGWMIHIGNGLYISNTHTARSSCSEIVTCSPTTDLCLVKGEVIRSVAQIA
RHDV2-MF421574  GLPGFMRHNGSGWMIHIGNGLYISNTHTARSSCSEIVTCSPTTDLCLVKGEVIRSVAQIA
RHDV2-MF421576  GLPGFMRHNGSGWMIHIGNGLYISNTHTARSSCSEIVTCSPTTDLCLVKGEVIRSVAQIA
RHDV2-MF421577  GLPGFMRHNGSGWMIHIGNGLYISNTHTARSSCSEIVTCSPTTDLCLVKGEVIRSVAQIA
RHDV2-MF421579  GLPGFMRHNGSGWMIHIGNGLYISNTHTARSSCSEIVTCSPTTDLCLVKGEVIRSVAQIA
RHDV2-MF421583  GLPGFMRHNGSGWMIHIGNGLYISNTHTARSSCSEIVTCSPTTDLCLVKGEVIRSVAQIA
RHDV2-MF421584  GLPGFMRHNGSGWMIHIGNGLYISNTHTARSSCSEIVTCSPTTDLCLVKGEVIRSVAQIA
RHDV2-MF421585  GLPGFMRHNGSGWMIHIGNGLYISNTHTARSSCSEIVTCSPTTDLCLVKGEVIRSVAQIA
RHDV2-MF421586  GLPGFMRHNGSGWMIHIGNGLYISNTHTARSSCSEIVTCSPTTDLCLVKGEVIRSVAQIA
RHDV2-MF421587  GLPGFMRHNGSGWMIHIGNGLYISNTHTARSSCSEIVTCSPTTDLCLVKGEVIRSVAQIA
RHDV2-MF421589  GLPGFMRHNGSGWMIHIGNGLYISNTHTARSSCSEIVTCSPTTDLCLVKGEVIRSVAQIA
RHDV2-MF421590  GLPGFMRHNGSGWMIHIGNGLYISNTHTARSSCSEIVTCSPTTDLCLVKGEVIRSVAQIA
RHDV2-MF421591  GLPGFMRHNGSGWMIHIGNGLYISNTHTARSSCSEIVTCSPTTDLCLVKGEVIRSVAQIA
RHDV2-MF421592  GLPGFMRHNGSGWMIHIGNGLYISNTHTARSSCSEIVTCSPTTDLCLVKGEVIRSVAQIA
RHDV2-MF421593  GLPGFMRHNGSGWMIHIGNGLYISNTHTARSSCSEIVTCSPTTDLCLVKGEVIRSVAQIA
RHDV2-MF421594  GLPGFMRHNGSGWMIHIGNGLYISNTHTARSSCSEIVTCSPTTDLCLVKGEVIRSVAQIA
RHDV2-MF421595  GLPGFMRHNGSGWMIHIGNGLYISNTHTARSSCSEIVTCSPTTDLCLVKGEVIRSVAQIA
RHDV2-MF421596  GLPGFMRHNGSGWMIHIGNGLYISNTHTARSSCSEIVTCSPTTDLCLVKGEVIRSVAQIA
RHDV2-MF421597  GLPGFMRHNGSGWMIHIGNGLYISNTHTARSSCSEIVTCSPTTDLCLVKGEVIRSVAQIA
RHDV2-MF421598  GLPGFMRHNGSGWMIHIGNGLYISNTHTARSSCSEIVTCSPTTDLCLVKGEVIRSVAQIA
RHDV2-MF421600  GLPGFMRHNGSGWMIHIGNGLYISNTHTARSSCSEIVTCSPTTDLCLVKGEVIRSVAQIA
RHDV2-MF421601  GLPGFMRHNGSGWMIHIGNGLYISNTHTARSSCSEIVTCSPTTDLCLVKGEVIRSVAQIA
RHDV2-MF421602  GLPGFMRHNGSGWMIHIGNGLYISNTHTARSSCSEIVTCSPTTDLCLVKGEVIRSVAQIA
RHDV2-MF421603  GLPGFMRHNGSGWMIHIGNGLYISNTHTARSSCSEIVTCSPTTDLCLVKGEVIRSVAQIA
RHDV2-MF421604  GLPGFMRHNGSGWMIHIGNGLYISNTHTARSSCSEIVTCSPTTDLCLVKGEVIRSVAQIA
RHDV2-MF421606  GLPGFMRHNGSGWMIHIGNGLYISNTHTARSSCSEIVTCSPTTDLCLVKGEVIRSVAQIA
RHDV2-MF421607  GLPGFMRHNGSGWMIHIGNGLYISNTHTARSSCSEIVTCSPTTDLCLVKGEVIRSVAQIA
RHDV2-MF421608  GLPGFMRHNGSGWMIHIGNGLYISNTHTARSSCSEIVTCSPTTDLCLVKGEVIRSVAQIA
RHDV2-MF421609  GLPGFMRHNGSGWMIHIGNGLYISNTHTARSSCSEIVTCSPTTDLCLVKGEVIRSVAQIA
RHDV2-MF421610  GLPGFMRHNGSGWMIHIGNGLYISNTHTARSSCSEIVTCSPTTDLCLVKGEVIRSVAQIA
RHDV2-MF421611  GLPGFMRHNGSGWMIHIGNGLYISNTHTARSSCSEIVTCSPTTDLCLVKGEVIRSVAQIA
RHDV2-MF421612  GLPGFMRHNGSGWMIHIGNGLYISNTHTARSSCSEIVTCSPTTDLCLVKGEVIRSVAQIA
RHDV2-MF421613  GLPGFMRHNGSGWMIHIGNGLYISNTHTARSSCSEIVTCSPTTDLCLVKGEVIRSVAQIA
RHDV2-MF421615  GLPGFMRHNGSGWMIHIGNGLYISNTHTARSSCSEIVTCSPTTDLCLVKGEVIRSVAQIA
RHDV2-MF421617  GLPGFMRHNGSGWMIHIGNGLYISNTHTARSSCSEIVTCSPTTDLCLVKGEVIRSVAQIA
RHDV2-MF421618  GLPGFMRHNGSGWMIHIGNGLYISNTHTARSSCSEIVTCSPTTDLCLVKGEVIRSVAQIA
RHDV2-MF421620  GLPGFMRHNGSGWMIHIGNGLYISNTHTARSSCSEIVTCSPTTDLCLVKGEVIRSVAQIA
RHDV2-MF421624  GLPGFMRHNGSGWMIHIGNGLYISNTHTARSSCSEIVTCSPTTDLCLVKGEVIRSVAQIA
RHDV2-MF421625  GLPGFMRHNGSGWMIHIGNGLYISNTHTARSSCSEIVTCSPTTDLCLVKGEVIRSVAQIA
RHDV2-MF421627  GLPGFMRHNGSGWMIHIGNGLYISNTHTARSSCSEIVTCSPTTDLCLVKGEVIRSVAQIA
RHDV2-MF421636  GLPGFMRHNGSGWMIHIGNGLYISNTHTARSSCSEIVTCSPTTDLCLVKGEVIRSVAQIA
RHDV2-MF421637  GLPGFMRHNGSGWMIHIGNGLYISNTHTARSSCSEIVTCSPTTDLCLVKGEVIRSVAQIA
RHDV2-MF421638  GLPGFMRHNGSGWMIHIGNGLYISNTHTARSSCSEIVTCSPTTDLCLVKGEVIRSVAQIA
RHDV2-MF421639  GLPGFMRHNGSGWMIHIGNGLYISNTHTARSSCSEIVTCSPTTDLCLVKGEVIRSVAQIA
```

RHDV2-MF421642	GLPGFMRHNGSGWMIHIGNGLYISNTHTARSSCSEIVTCSPTTDLCLVKGEVIRSVAQIA
RHDV2-MF421643	GLPGFMRHNGSGWMIHIGNGLYISNTHTARSSCSEIVTCSPTTDLCLVKGEVIRSVAQIA
RHDV2-MF421645	GLPGFMRHNGSGWMIHIGNGLYISNTHTARSSCSEIVTCSPTTDLCLVKGEVIRSVAQIA
RHDV2-MF421646	GLPGFMRHNGSGWMIHIGNGLYISNTHTARSSCSEIVTCSPTTDLCLVKGEVIRSVAQIA
RHDV2-MF421647	GLPGFMRHNGSGWMIHIGNGLYISNTHTARSSCSEIVTCSPTTDLCLVKGEVIRSVAQIA
RHDV2-MF421648	GLPGFMRHNGSGWMIHIGNGLYISNTHTARSSCSEIVTCSPTTDLCLVKGEVIRSVAQIA
RHDV2-MF421649	GLPGFMRHNGSGWMIHIGNGLYISNTHTARSSCSEIVTCSPTTDLCLVKGEVIRSVAQIA
RHDV2-MF421650	GLPGFMRHNGSGWMIHIGNGLYISNTHTARSSCSEIVTCSPTTDLCLVKGEVIRSVAQIA
RHDV2-MF421651	GLPGFMRHNGSGWMIHIGNGLYISNTHTARSSCSEIVTCSPTTDLCLVKGEVIRSVAQIA
RHDV2-MF421652	GLPGFMRHNGSGWMIHIGNGLYISNTHTARSSCSEIVTCSPTTDLCLVKGEVIRSVAQIA
RHDV2-MF421654	GLPGFMRHNGSGWMIHIGNGLYISNTHTARSSCSEIVTCSPTTDLCLVKGEVIRSVAQIA
RHDV2-MF421655	GLPGFMRHNGSGWMIHIGNGLYISNTHTARSSCSEIVTCSPTTDLCLVKGEVIRSVAQIA
RHDV2-MF421656	GLPGFMRHNGSGWMIHIGNGLYISNTHTARSSCSEIVTCSPTTDLCLVKGEVIRSVAQIA
RHDV2-MF421658	GLPGFMRHNGSGWMIHIGNGLYISNTHTARSSCSEIVTCSPTTDLCLVKGEVIRSVAQIA
RHDV2-MF421659	GLPGFMRHNGSGWMIHIGNGLYISNTHTARSSCSEIVTCSPTTDLCLVKGEVIRSVAQIA
RHDV2-MF421660	GLPGFMRHNGSGWMIHIGNGLYISNTHTARSSCSEIVTCSPTTDLCLVKGEVIRSVAQIA
RHDV2-MF421661	GLPGFMRHNGSGWMIHIGNGLYISNTHTARSSCSEIVTCSPTTDLCLVKGEVIRSVAQIA
RHDV2-MF421663	GLPGFMRHNGSGWMIHIGNGLYISNTHTARSSCSEIVTCSPTTDLCLVKGEVIRSVAQIA
RHDV2-MF421664	GLPGFMRHNGSGWMIHIGNGLYISNTHTARSSCSEIVTCSPTTDLCLVKGEVIRSVAQIA
RHDV2-MF421665	GLPGFMRHNGSGWMIHIGNGLYISNTHTARSSCSEIVTCSPTTDLCLVKGEVIRSVAQIA
RHDV2-MF421666	GLPGFMRHNGSGWMIHIGNGLYISNTHTARSSCSEIVTCSPTTDLCLVKGEVIRSVAQIA
RHDV2-MF421667	GLPGFMRHNGSGWMIHIGNGLYISNTHTARSSCSEIVTCSPTTDLCLVKGEVIRSVAQIA
RHDV2-MF421668	GLPGFMRHNGSGWMIHIGNGLYISNTHTARSSCSEIVTCSPTTDLCLVKGEVIRSVAQIA
RHDV2-MF421669	GLPGFMRHNGSGWMIHIGNGLYISNTHTARSSCSEIVTCSPTTDLCLVKGEVIRSVAQIA
RHDV2-MF421670	GLPGFMRHNGSGWMIHIGNGLYISNTHTARSSCSEIVTCSPTTDLCLVKGEVIRSVAQIA
RHDV2-MF421673	GLPGFMRHNGSGWMIHIGNGLYISNTHTARSSCSEIVTCSPTTDLCLVKGEVIRSVAQIA
RHDV2-MF421674	GLPGFMRHNGSGWMIHIGNGLYISNTHTARSSCSEIVTCSPTTDLCLVKGEVIRSVAQIA
RHDV2-MF421675	GLPGFMRHNGSGWMIHIGNGLYISNTHTARSSCSEIVTCSPTTDLCLVKGEVIRSVAQIA
RHDV2-MF421676	GLPGFMRHNGSGWMIHIGNGLYISNTHTARSSCSEIVTCSPTTDLCLVKGEVIRSVAQIA
RHDV2-MF421678	GLPGFMRHNGSGWMIHIGNGLYISNTHTARSSCSEIVTCSPTTDLCLVKGEVIRSVAQIA
RHDV2-MF421680	GLPGFMRHNGSGWMIHIGNGLYISNTHTARSSCSEIVTCSPTTDLCLVKGEVIRSVAQIA
RHDV2-MF421681	GLPGFMRHNGSGWMIHIGNGLYISNTHTARSSCSEIVTCSPTTDLCLVKGEVIRSVAQIA
RHDV2-MF421682	GLPGFMRHNGSGWMIHIGNGLYISNTHTARSSCSEIVTCSPTTDLCLVKGEVIRSVAQIA
RHDV2-MF421683	GLPGFMRHNGSGWMIHIGNGLYISNTHTARSSCSEIVTCSPTTDLCLVKGEVIRSVAQIA
RHDV2-MF421684	GLPGFMRHNGSGWMIHIGNGLYISNTHTARSSCSEIVTCSPTTDLCLVKGEVIRSVAQIA
RHDV2-MF421685	GLPGFMRHNGSGWMIHIGNGLYISNTHTARSSCSEIVTCSPTTDLCLVKGEVIRSVAQIA
RHDV2-MF421689	GLPGFMRHNGSGWMIHIGNGLYISNTHTARSSCSEIVTCSPTTDLCLVKGEVIRSVAQIA
RHDV2-MF421691	GLPGFMRHNGSGWMIHIGNGLYISNTHTARSSCSEIVTCSPTTDLCLVKGEVIRSVAQIA
RHDV2-MF421694	GLPGFMRHNGSGWMIHIGNGLYISNTHTARSSCSEIVTCSPTTDLCLVKGEVIRSVAQIA
RHDV2-MF421695	GLPGFMRHNGSGWMIHIGNGLYISNTHTARSSCSEIVTCSPTTDLCLVKGEVIRSVAQIA
RHDV2-MF421696	GLPGFMRHNGSGWMIHIGNGLYISNTHTARSSCSEIVTCSPTTDLCLVKGEVIRSVAQIA
RHDV2-MG763936	GLPGFMRHNGSGWMIHIGNGLYISNTHTARSSCSEVVTCSPTTDLCLVKGEVIRSVAQIA
RHDV2-MG763947	GLPGFMRHNGSGWMIHIGNGLYISNTHTARSSCSEVVTCSPTTDLCLVKGEVIRSVAQIA
RHDV2-MW460124	GLPGFMRHNGSGWMIHIGNGLYISNTHTARSSCSEVVTCSPTTDLCLVKGEVIRSVAQIA

```
RHDV2-MW460125  GLPGFMRHNGSGWMIHIGNGLYISNTHTARSSCSEVVTCSPTTDLCLVKGEVIRSVAQIA
RHDV2-MW460129  GLPGFMRHNGSGWMIHIGNGLYISNTHTARSSCSEVVTCSPTTDLCLVKGEVIRSVAQIA
RHDV2-MW460147  GLPGFMRHNGSGWMIHIGNGLYISNTHTARSSCSEVVTCSPTTDLCLVKGEVIRSVAQIA
RHDV2-MW460148  GLPGFMRHNGSGWMIHIGNGLYISNTHTARSSCSEVVTCSPTTDLCLVKGEVIRSVAQIA
RHDV2-MW460231  GLPGFMRHNGSGWMIHIGNGLYISNTHTARSSCSEVVTCSPTTDLCLVKGEVIRSVAQIA
RHDV2-MW460234  GLPGFMRHNGSGWMIHIGNGLYISNTHTARSSCSEVVTCSPTTDLCLVKGEVIRSVAQIA
RHDV2-MW460237  GLPGFMRHNGSGWMIHIGNGLYISNTHTARSSCSEVVTCSPTTDLCLVKGEVIRSVAQIA
RHDV2-MW460240  GLPGFMRHNGSGWMIHIGNGLYISNTHTARSSCSEVVTCSPTTDLCLVKGEVIRSVAQIA
RHDV2-MF421628  GLPGFMRHNGSGWMIHIGNGLYISNTHTARSSCSEVVTCSPTTDLCLVKGEVIRSVAQIA
RHDV2-MF421629  GLPGFMRHNGSGWMIHIGNGLYISNTHTARSSCSEVVTCSPTTDLCLVKGEVIRSVAQIA
RHDV2-MF421630  GLPGFMRHNGSGWMIHIGNGLYISNTHTARSSCSEVVTCSPTTDLCLVKGEVIRSVAQIA
RHDV2-MF421631  GLPGFMRHNGSGWMIHIGNGLYISNTHTARSSCSEVVTCSPTTDLCLVKGEVIRSVAQIA
RHDV2-MF421633  GLPGFMRHNGSGWMIHIGNGLYISNTHTARSSCSEVVTCSPTTDLCLVKGEVIRSVAQIA
RHDV2-MF421634  GLPGFMRHNGSGWMIHIGNGLYISNTHTARSSCSEVVTCSPTTDLCLVKGEVIRSVAQIA
RHDV2-MF421635  GLPGFMRHNGSGWMIHIGNGLYISNTHTARSSCSEVVTCSPTTDLCLVKGEVIRSVAQIA
RHDV2-MF421697  GLPGFMRHNGSGWMIHIGNGLYISNTHTARSSCSEVVTCSPTTDLCLVKGEVIRSVAQIA
RHDV2-MF421698  GLPGFMRHNGSGWMIHIGNGLYISNTHTARSSCSEVVTCSPTTDLCLVKGEVIRSVAQIA
RHDV2-MF421699  GLPGFMRHNGSGWMIHIGNGLYISNTHTARSSCSEVVTCSPTTDLCLVKGEVIRSVAQIA
RHDV2-MF421605  GLPGFMRHNGSGWMIHIGNGLYISNTHTARSSCSEIVTCSPTTDLCLVKGEVVRSVAQIA
RHDV2-MN737112  GLPGFMRHNGSGWMIHIGNGLYISNTHTARSSCSEIVTCSPTTDLCLVKGEVIRSVAQIA
RHDV2-MF421614  GLPGFMRHNGSGWMIHIGNGLYISNTHTARSSCSEIVTCSPTTDLCLVKGEVIRSVAQIA
RHDV2-MF421657  GLPGFMRHNGSGWMIHIGNGLYISNTHTARSSCSEIVTCSPTTDLCLVKGEVIRSVAQIA
RHDV2-MF421671  GLPGFMRHNGSGWMIHIGNGLYISNTHTARSSCSEIVTCSPTTDLCLVKGEVIRSVAQIA
RHDV2-MW460108  GLPGFMRHNGSGWMVHIGNGLYISNTHTARSSCSEIVTCSPTTDLCLVKGEVIRSVAQIA
RHDV2-MW460109  GLPGFMRHNGSGWMVHIGNGLYISNTHTARSSCSEIVTCSPTTDLCLVKGEVIRSVAQIA
RHDV2-MW460201  GLPGFMRHNGSGWMVHIGNGLYISNTHTARSSCSEIVTCSPTTDLCLVKGEVIRSVAQIA
RHDV2-MF421575  GLPGFMRHNGSGWMVHIGNGLYISNTHTARSSCSEIVTCSPTTDLCLVKGEVIRSVAQIA
RHDV2-MF421580  GLPGFMRHNGSGWMVHIGNGLYISNTHTARSSCSEIVTCSPTTDLCLVKGEVIRSVAQIA
RHDV2-MF421581  GLPGFMRHNGSGWMVHIGNGLYISNTHTARSSCSEIVTCSPTTDLCLVKGEVIRSVAQIA
RHDV2-MF421582  GLPGFMRHNGSGWMVHIGNGLYISNTHTARSSCSEIVTCSPTTDLCLVKGEVIRSVAQIA
RHDV2-MF421672  GLPGFMRHNGSGWMVHIGNGLYISNTHTARSSCSEIVTCSPTTDLCLVKGEVIRSVAQIA
RHDV2-MW460164  GLPGFMRHNGSGWMIHIGNGLYISNTHTARSSCSEIVTCSPTTDLCLVKGEVIRSVAQIA
RHDV2-MW460174  GLPGFMRHNGSGWMIHIGNGLYISNTHTARSSCSEIVTCSPTTDLCLVKGEVIRSVAQIA
RHDV2-MF421619  GLPGFMRHNGSGWMIHIGNGLYISNTHTARSSCSEIVTCSPTTDLCLVKGEVIRSVAQIA
RHDV2-MW460168  GLPGFMRHNGSGWMIHIGNGLYISNTHTARSSCSEIVTCSPTTDLCLVKGEVIRSVAQIA
RHDV2-MW460171  GLPGFMRHNGSGWMIHIGNGLYISNTHTARSSCSEIVTCSPTTDLCLVKGEVIRSVAQIA
RHDV2-MW460229  GLPGFMRHNGSGWMIHIGNGLYISNTHTARSSCSEIITCSPTTDLCLVKGEVIRSVAQIA
RHDV2-MF421616  GLPGFMRHNGSGWMIHIGNGLYISNTHTARSSCSEIITCSPTTDLCLVKGEVIRSVAQIA
RHDV2-MF421621  GLPGFMRHNGSGWMIHIGNGLYISNTHTARSSCSEIITCSPTTDLCLVKGEVIRSVAQIA
RHDV2-MF421623  GLPGFMRHNGSGWMIHIGNGLYISNTHTARSSCSEIITCSPTTDLCLVKGEVIRSVAQIA
RHDV2-MF421686  GLPGFMRHNGSGWMIHIGNGLYISNTHTARSSCSEIITCSPTTDLCLVKGEVIRSVAQIA
RHDV2-MF421687  GLPGFMRHNGSGWMIHIGNGLYISNTHTARSSCSEIITCSPTTDLCLVKGEVIRSVAQIA
RHDV2-MF421688  GLPGFMRHNGSGWMIHIGNGLYISNTHTARSSCSEIITCSPTTDLCLVKGEVIRSVAQIA
RHDV2-MF421588  GLPGFMRHNGSGWMIHIGNGLYISNTHTARSSCSEIVTCSPTTDLCLVKGEVIRSVAQIA
```

```
RHDV2-MF421692  GLPGFMRHNGSGWMIHIGNGLYISNTHTARSSCSEIVTCSPTTDLCLVKGEVIRSVAQIA
RHDV2-MF421640  GLPGFMRHNGSGWMIHIGNGLYISNTHTAKSSCSEIVTCSPTTDLCLVKGEVIRSVAQIA
RHDV2-MF421641  GLPGFMRHNGSGWMIHIGNGLYISNTHTAKSSCSEIVTCSPTTDLCLVKGEVIRSVAQIA
RHDV2-MF421653  GLPGFMRHNGSGWMIHIGNGLYISNTHTAKSSCSEIVTCSPTTDLCLVKGEVIRSVAQIA
RHDV2-MF421677  GLPGFMRHNGSGWMIHIGNGLYISNTHTAKSSCSEIVTCSPTTDLCLVKGEVIRSVAQIA
RHDV2-MF421679  GLPGFMRHNGSGWMIHIGNGLYISNTHTAKSSCSEIVTCSPTTDLCLVKGEVIRSVAQIA
RHDV2-MF421693  GLPGFMRHNGSGWMIHIGNGLYISNTHTAKSSCSEIVTCSPTTDLCLVKGEVIRSVAQIA
RHDV2-MF421644  GLPGFMRHNGSGWMIHIGNGLYISNTHTARSSCSEIVTCSPTTDLCLVKGEVIRSVAQIA
RHDV2-MF421632  GLPGFMRHNGSGWMIHIGNGLYISNTHTARSSCSEVVTCSPTTDLCLVKGEVIRSVAQIA
RHDV2-MF421700  GLPGFMRHNGSGWMIHIGNGLYISNTHTARSSCSEVVTCSPTTDLCLVKGEVIRSVAQIA
RHDV2-MW460132  GLPGFMRHNGSGWMIHIGNGLYISNTHTARSSCSEVVTCSPTTDLCLVKGEVIRSVAQIA
RHDV2-MW460133  GLPGFMRHNGSGWMIHIGNGLYISNTHTARSSCSEVVTCSPTTDLCLVKGEVIRSVAQIA
RHDV2-MW460134  GLPGFMRHNGSGWMIHIGNGLYISNTHTARSSCSEVVTCSPTTDLCLVKGEVIRSVAQIA
RHDV2-MW460143  GLPGFMRHNGSGWMIHIGNGLYISNTHTARSSCSEVVTCSPTTDLCLVKGEVIRSVAQIA
RHDV2-MF407651  GLPGFMRHNGSGWMIHIGNGLYISNTHTARSSCSEIVTCSPTTDLCLVKGEAIRSVAQIA
RHDV2-MF407652  GLPGFMRHNGSGWMIHIGNGLYISNTHTARSSCSEIVTCSPTTDLCLVKGEAIRSVAQIA
RHDV2-MF421567  GLPGFMRHNGSGWMIHIGNGLYISNTHTARSSCSEIVTCSPTTDLCLVKGEAIRSVAQIA
RHDV2-MF421568  GLPGFMRHNGSGWMIHIGNGLYISNTHTARSSCSEIVTCSPTTDLCLVKGEAIRSVAQIA
RHDV2-MF421571  GLPGFMRHNGSGWMIHIGNGLYISNTHTARSSCSEIVTCSPTTDLCLVKGEAIRSVAQIA
RHDV2-MF421572  GLPGFMRHNGSGWMIHIGNGLYISNTHTARSSCSEIVTCSPTTDLCLVKGEAIRSVAQIA
RHDV2-MF421573  GLPGFMRHNGSGWMIHIGNGLYISNTHTARSSCSEIVTCSPTTDLCLVKGEAIRSVAQIA
RHDV-MH190418   GLPGFMRHNGSGWMIHIGNGLYISNTHTARSSCSEIVTCSPTTDLCLVKGEAIRSVAQIA
RHDV-EU003579   GLPGFMRHNGSGWMIHIGNGLYISNTHTARSSCSEIVTCSPTTDLCLVKGEAIRSVAQIA
RHDV-EU003580   GLPGFMRHNGSGWMIHIGNGLYISNTHTARSSCSEIVTCSPTTDLCLVKGEAIRSVAQIA
RHDV-KP144789   GLPGFMRHNGSGWMIHIGNGLYISNTHTARSSCSEIVTCSPTTDLCLVKGEAIRSVAQIA
RHDV-KU882093   GLPGFMRHNGSGWMIHIGNGLYISNTHTARSSCSEIVTCSPTTDLCLVKGEAIRSVAQIA
RHDV-KU882092   GLPGFMRHNGSGWMIHIGNGLYISNTHTARSSCSEIVTCSPTTDLCLVKGEAIRSVAQIA
RHDV-X87607     GLPGFMRHNGSGWMIHIGNGLYISNTHTARSSCSEIVTCSPTTDLCLVKGEAIRSVAQIA
RHDV-Z29514     GLPGFMRHNGSGWMIHIGNGLYISNTHTARSSCSEVVTCSPTTDLCLVKGEAIRSVAQIA
RHDV-M67473     GLPGFMRHNGSGWMIHIGNGLYISNTHTARSSCSEIVTCSPTTDLCLVKGESIRSVAQIA
RHDV-MG602005   GLPGFMRHNGSGWMIHIGNGLYISNTHTARSSCSEIVTCSPTTDLCLVKGETIRSVAQIA
RHDV-KY679902   GLPGFMRHNGSGWMIHIGNGLYISNTHTARSSCSEIVTCSPTTDLCLVKGETIRSVAQIA
RHDV-KY319031   GLPGFMRHNGSGWMIHIGNGLYISNTHTARSSCSEIVTCSPTTDLCLVKGETIRSVAQIA
RHDV-KY319032   GLPGFMRHNGSGWMIHIGNGLYISNTHTARSSCSEIVTCSPTTDLCLVKGETIRSVAQIA
RHDV-KY319034   GLPGFMRHNGSGWMIHIGNGLYISNTHTARSSCSEIVTCSPTTDLCLVKGETIRSVAQIA
RHDV-KY319035   GLPGFMRHNGSGWMIHIGNGLYISNTHTARSSCSEIVTCSPTTDLCLVKGETIRSVAQIA
RHDV-KF677011   GLPGFMRHNGSGWMIHIGNGLYISNTHTARSSCSEIVTCSPTTDLCLVKGETIRSVAQIA
RHDV-EU003578   GLPGFMRHNGSGWMIHIGNGLYISNTHTARSSCSEIVTCSPTTDLCLVKGETIRSVAQIA
RHDV-EU003582   GLPGFMRHNGSGWMIHIGNGLYISNTHTARSSCSEIVTCSPTTDLCLVKGETIRSVAQIA
RHDV-MW460041   GLPGFMRHNGSGWMIHIGNGLYISNTHTARSSCSEIVTCSPTTDLCLVKGETIRSVAQIA
RHDV-MW460063   GLPGFMRHNGSGWMIHIGNGLYISNTHTARSSCSEIVTCSPTTDLCLVKGETIRSVAQIA
RHDV-MW460075   GLPGFMRHNGSGWMIHIGNGLYISNTHTARSSCSEIVTCSPTTDLCLVKGETIRSVAQIA
RHDV-MW460094   GLPGFMRHNGSGWMIHIGNGLYISNTHTARSSCSEIVTCSPTTDLCLVKGETIRSVAQIA
RHDV-MW460104   GLPGFMRHNGSGWMIHIGNGLYISNTHTARSSCSEIVTCSPTTDLCLVKGETIRSVAQIA
```

```
RHDV-MW460139   GLPGFMRHNGSGWMIHIGNGLYISNTHTARSSCSEIVTCSPTTDLCLVKGETIRSVAQIA
RHDV-MW460154   GLPGFMRHNGSGWMIHIGNGLYISNTHTARSSCSEIVTCSPTTDLCLVKGETIRSVAQIA
RHDV-MW460184   GLPGFMRHNGSGWMIHIGNGLYISNTHTARSSCSEIVTCSPTTDLCLVKGETIRSVAQIA
RHDV-MW460196   GLPGFMRHNGSGWMIHIGNGLYISNTHTARSSCSEIVTCSPTTDLCLVKGETIRSVAQIA
RHDV-MW460209   GLPGFMRHNGSGWMIHIGNGLYISNTHTARSSCSEIVTCSPTTDLCLVKGETIRSVAQIA
RHDV-MW460216   GLPGFMRHNGSGWMIHIGNGLYISNTHTARSSCSEIVTCSPTTDLCLVKGETIRSVAQIA
RHDV-MW460235   GLPGFMRHNGSGWMIHIGNGLYISNTHTARSSCSEIVTCSPTTDLCLVKGETIRSVAQIA
RHDV-MW460239   GLPGFMRHNGSGWMIHIGNGLYISNTHTARSSCSEIVTCSPTTDLCLVKGETIRSVAQIA
RHDV-MW460241   GLPGFMRHNGSGWMIHIGNGLYISNTHTARSSCSEIVTCSPTTDLCLVKGETIRSVAQIA
RHDV-MW460242   GLPGFMRHNGSGWMIHIGNGLYISNTHTARSSCSEIVTCSPTTDLCLVKGETIRSVAQIA
RHDV-MN853658   GLPGFMRHNGSGWMIHIGNGLYISNTHTARSSCSEIVTCSPTTDLCLVKGETIRSVAQIA
RHDV-MF598301   GLPGFMRHNGSGWMIHIGNGLYISNTHTARSSCSEIVTCSPTTDLCLVKGETIRSVAQIA
RHDV-KY679905   GLPGFMRHNGSGWMIHIGNGLYISNTHTARSSCSEIVTCSPTTDLCLVKGETIRSVAQIA
RHDV-KY679904   GLPGFMRHNGSGWMIHIGNGLYISNTHTARSSCSEIVTCSPTTDLCLVKGETIRSVAQIA
RHDV-KY679903   GLPGFMRHNGSGWMIHIGNGLYISNTHTARSSCSEIVTCSPTTDLCLVKGETIRSVAQIA
RHDV-KY319033   GLPGFMRHNGSGWMIHIGNGLYISNTHTARSSCSEIVTCSPTTDLCLVKGETIRSVAQIA
RHDV-EU003581   GLPGFMRHNGSGWMIHIGNGLYISNTHTARSSCSEIVTCSPTTDLCLVKGETIRSVAQIA
RHDV-MT628289   GLPGFMRHNGSGWMIHIGNGLYISNTHTARSSCSQIVTCSPTTDLCLVKGEAIRSVAQIA
RHDV2-MW460166  GLPGFMRHNGSGWMIHIGNGLYISNTHTARSSCSEIVTCSPTTDLCLVKGEVIRSVAQIA
RHDV2-MF421565  GLPGFMRHNGSGWMIHIGNGLYISNTHTARSSCSEIVTCSPTTDLCLVKGEAIRSVAQIA
RHDV2-MF421566  GLPGFMRHNGSGWMIHIGNGLYISNTHTARSSCSEIVTCSPTTDLCLVKGEAIRSVAQIA
RHDV2-MF421569  GLPGFMRHNGSGWMIHIGNGLYISNTHTARSSCSEIVTCSPTTDLCLVKGEAIRSVAQIA
RHDV2-MF421570  GLPGFMRHNGSGWMIHIGNGLYISNTHTARSSCSEIVTCSPTTDLCLVKGEAIRSVAQIA
RHDV2-KY628307  GLPGFMRHNGSGWMIHIGNGLYISNTHTARSSCSEIVTCSPTTDLCLVKGEAIRSVAQIA
RHDV2-MF421690  GLPGFMRHNGSGWMIHIGNGLYISNTHTARSSCSEIVTCSPTTDLCLVKGEVIRSVAQIA
RHDV2-MF421563  GLPGFMRHNGSGWMIHIGNGLYISNTHTARSSCSEIVTCSPNTDLCLVKGEVIRSVAQIA
RHDV2-MF421564  GLPGFMRHNGSGWMIHIGNGLYISNTHTARSSCSEIVTCSPNTDLCLVKGEVIRSVAQIA
RHDV2-KT280058  GLPGFMRHNGSGWMIHIGNGLYISNTHTARSSCSEIVTCSPNTDLCLVKGEVIRSVAQIA
RHDV2-KT280059  GLPGFMRHNGSGWMIHIGNGLYISNTHTARSSCSEIVTCSPNTDLCLVKGEVIRSVAQIA
RHDV2-KY628318  GLPGFMRHNGSGWMIHIGNGLYISNTHTARSSCSEIVTCSPNTDLCLVKGEAIRSVAQIA
RHDV2-KY628319  GLPGFMRHNGSGWMIHIGNGLYISNTHTARSSCSEIVTCSPNTDLCLVKGEAIRSVAQIA
RHDV2-MF421578  GLPGFMRHNGSGWMTHIGNGLYISNTHTARSSCSEIITCSPTTDLCLVKGEVIRSVAQIA
RHDV2-MF421622  GLPGFMRHNGSGWMTHIGNGLYISNTHTARSSCSEIITCSPTTDLCLVKGEVIRSVAQIA
RHDV2-MF421626  GLPGFMRHNGSGWMTHIGNGLYISNTHTARSSCSEIITCSPTTDLCLVKGEVIRSVAQIA
RHDV2-MW460167  GLPGFMRHNGSGWMIHIGNGLYISNTHTARSSGSEIVTCSPTTDLCLVKGEVIRSVAQIA
RHDV2-MF421599  GLPGFMRHNGSGWMIHIGNGLYISNTHTARSSCSEIVTCSPTTDLCLVKGEVIRSVAQIA
RHDV2-MF421662  GLPGFMRHNGSGWMIHIGNGLYISNTHTARSSCSEIVTCSPTTDLCLVKGEVIRSVAQIA
RHDV-U54983     GLPGFMRHNGSGRMIHIGNGLYISNTHTARSSCSEIVTCSPTTDLCLVKGEAIRSVAQIA
RHDV2-MG763941  GLPGFMRHNGSGWMIHIGNGMYISNTHTAKSSCSEIVTCSPTTDLCLVKGETIRSVAQIA
RHDV2-MG763944  GLPGFMRHNGSGWMIHIGNGMYISNTHTAKSSCSEIVTCSPTTDLCLVKGETIRSVAQIA
RHDV2-MG763946  GLPGFMRHNGSGWMIHIGNGMYISNTHTAKSSCSEIVTCSPTTDLCLVKGETIRSVAQIA
RHDV2-MG763952  GLPGFMRHNGSGWMIHIGNGMYISNTHTAKSSCSEIVTCSPTTDLCLVKGETIRSVAQIA
RHDV2-MG763954  GLPGFMRHNGSGWMIHIGNGMYISNTHTAKSSCSEIVTCSPTTDLCLVKGETIRSVAQIA
RHDV2-MW460019  GLPGFMRHNGSGWMIHIGNGMYISNTHTARSSCSDIVTCSPTTDLCLVKGEVIRSVAQIS
```

```
RHDV2-MW460192  GLPGFMRHNGSGWMIHIGNGMYISNTHTARSSCSDIVTCSPTTDLCLVKGEVIRSVAQIS
RHDV2-MF598302  GLPGFMRHNGSGWMIHIGNGMYISNTHTARSSCSDIVTCSPTTDLCLVKGEVIRSVAQIS
RHDV-KY628306   GLPGFMRHNGSGWMIHIGNGMYISNTHTARSSCSDIVTCSPTTDLCLVKGEVIRSVAQIS
RHDV-KY628308   GLPGFMRHNGSGWMIHIGNGMYISNTHTARSSCSDIVTCSPTTDLCLVKGEVIRSVAQIS
RHDV-KY628309   GLPGFMRHNGSGWMIHIGNGMYISNTHTARSSCSDIVTCSPTTDLCLVKGEVIRSVAQIS
RHDV-KY628310   GLPGFMRHNGSGWMIHIGNGMYISNTHTARSSCSDIVTCSPTTDLCLVKGEVIRSVAQIS
RHDV-KY628311   GLPGFMRHNGSGWMIHIGNGMYISNTHTARSSCSDIVTCSPTTDLCLVKGEVIRSVAQIS
RHDV-KY628312   GLPGFMRHNGSGWMIHIGNGMYISNTHTARSSCSDIVTCSPTTDLCLVKGEVIRSVAQIS
RHDV-KY628313   GLPGFMRHNGSGWMIHIGNGMYISNTHTARSSCSDIVTCSPTTDLCLVKGEVIRSVAQIS
RHDV-KY628314   GLPGFMRHNGSGWMIHIGNGMYISNTHTARSSCSDIVTCSPTTDLCLVKGEVIRSVAQIS
RHDV-KY628315   GLPGFMRHNGSGWMIHIGNGMYISNTHTARSSCSDIVTCSPTTDLCLVKGEVIRSVAQIS
RHDV-KY628316   GLPGFMRHNGSGWMIHIGNGMYISNTHTARSSCSDIVTCSPTTDLCLVKGEVIRSVAQIS
RHDV-KY628317   GLPGFMRHNGSGWMIHIGNGMYISNTHTARSSCSDIVTCSPTTDLCLVKGEVIRSVAQIS
RHDV-KY628320   GLPGFMRHNGSGWMIHIGNGMYISNTHTARSSCSDIVTCSPTTDLCLVKGEVIRSVAQIS
RHDV-MW460097   GLPGFMRHNGSGWMIHIGNGMYISNTHTARSSCSDIVTCSPTTDLCLVKGEVIRSVAQIS
RHDV2-MW460118  GLPGFMRHNGSGWMIHIGNGMYISNTHTARSSCSDIVTCSPTTDLCLVKGEVIRSVAQIS
RHDV2-MW460020  GLPGFMRHNGSGWMIHIGNGMYISNTHTARSSCSDIVTCSPTTDLCLVKGEVIRSVAQIS
RHDV2-MW460228  GLPGFMRHNGSGWMIHIGNGMYISNTHTARSSCSDIVTCSPTTDLCLVKGEVIRSVAQIS
RHDV2-MF421701  GLPGFMRHNGSGWMIHIGNGMYISNTHTARSSCSDIVTCSPTTDLCLVKGEVIRSVAQIS
RHDV2-MW460107  GLPGFMRHNGSGWMIHIGSGMYISNTHTARSSCSEIVTCSPTTDLCLVKGEVIRSVAQIA
RHDV2-MW460120  GLPGFMRHNGSGWMIHIGSGMYISNTHTARSSCSEIVTCSPTTDLCLVKGEVIRSVAQIA
RHDV2-MW460122  GLPGFMRHNGSGWMIHIGSGMYISNTHTARSSCSEIVTCSPTTDLCLVKGEVIRSVAQIA
RHDV2-MW460123  GLPGFMRHNGSGWMIHIGSGMYISNTHTARSSCSEIVTCSPTTDLCLVKGEVIRSVAQIA
RHDV2-MW460127  GLPGFMRHNGSGWMIHIGSGMYISNTHTARSSCSEIVTCSPTTDLCLVKGEVIRSVAQIA
RHDV2-MW460128  GLPGFMRHNGSGWMIHIGSGMYISNTHTARSSCSEIVTCSPTTDLCLVKGEVIRSVAQIA
RHDV2-MW460130  GLPGFMRHNGSGWMIHIGSGMYISNTHTARSSCSEIVTCSPTTDLCLVKGEVIRSVAQIA
RHDV2-MW460131  GLPGFMRHNGSGWMIHIGSGMYISNTHTARSSCSEIVTCSPTTDLCLVKGEVIRSVAQIA
RHDV2-MW460135  GLPGFMRHNGSGWMIHIGSGMYISNTHTARSSCSEIVTCSPTTDLCLVKGEVIRSVAQIA
RHDV2-MW460137  GLPGFMRHNGSGWMIHIGSGMYISNTHTARSSCSEIVTCSPTTDLCLVKGEVIRSVAQIA
RHDV2-MW460140  GLPGFMRHNGSGWMIHIGSGMYISNTHTARSSCSEIVTCSPTTDLCLVKGEVIRSVAQIA
RHDV2-MW460141  GLPGFMRHNGSGWMIHIGSGMYISNTHTARSSCSEIVTCSPTTDLCLVKGEVIRSVAQIA
RHDV2-MW460142  GLPGFMRHNGSGWMIHIGSGMYISNTHTARSSCSEIVTCSPTTDLCLVKGEVIRSVAQIA
RHDV2-MW460144  GLPGFMRHNGSGWMIHIGSGMYISNTHTARSSCSEIVTCSPTTDLCLVKGEVIRSVAQIA
RHDV2-MW460149  GLPGFMRHNGSGWMIHIGSGMYISNTHTARSSCSEIVTCSPTTDLCLVKGEVIRSVAQIA
RHDV2-MW460163  GLPGFMRHNGSGWMIHIGSGMYISNTHTARSSCSEIVTCSPTTDLCLVKGEVIRSVAQIA
RHDV2-MW460169  GLPGFMRHNGSGWMIHIGSGMYISNTHTARSSCSEIVTCSPTTDLCLVKGEVIRSVAQIA
RHDV2-MW460170  GLPGFMRHNGSGWMIHIGSGMYISNTHTARSSCSEIVTCSPTTDLCLVKGEVIRSVAQIA
RHDV2-MW460181  GLPGFMRHNGSGWMIHIGSGMYISNTHTARSSCSEIVTCSPTTDLCLVKGEVIRSVAQIA
RHDV2-MW460230  GLPGFMRHNGSGWMIHIGSGMYISNTHTARSSCSEIVTCSPTTDLCLVKGEVIRSVAQIA
RHDV2-MW460236  GLPGFMRHNGSGWMIHIGSGMYISNTHTARSSCSEIVTCSPTTDLCLVKGEVIRSVAQIA
RHDV2-MW460156  GLPGFMRHNGSGWMIHIGSGMYISNTHTARSSCSEIVTCSPTTDLCLVRGEVIRSVAQIA
RHDV2-MW460110  GLPGFMRHNGSGWMIHIGNGMYISNTHTARSSCSEIVTCSPTTDLCLVKGEVIRSVAQIA
RHDV2-MW460111  GLPGFMRHNGSGWMIHIGNGMYISNTHTARSSCSEIVTCSPTTDLCLVKGEVIRSVAQIA
RHDV2-MW460117  GLPGFMRHNGSGWMIHIGNGMYISNTHTARSSCSEIVTCSPTTDLCLVKGEVIRSVAQIA
```

RHDV2-MW460119	GLPGFMRHNGSGWMIHIGNGMYISNTHTARSSCSEIVTCSPTTDLCLVKGEVIRSVAQIA
RHDV2-MW460121	GLPGFMRHNGSGWMIHIGNGMYISNTHTARSSCSEIVTCSPTTDLCLVKGEVIRSVAQIA
RHDV2-MW460146	GLPGFMRHNGSGWMIHIGNGMYISNTHTARSSCSEIVTCSPTTDLCLVKGEVIRSVAQIA
RHDV2-MW460151	GLPGFMRHNGSGWMIHIGNGMYISNTHTARSSCSEIVTCSPTTDLCLVKGEVIRSVAQIA
RHDV2-MW460152	GLPGFMRHNGSGWMIHIGNGMYISNTHTARSSCSEIVTCSPTTDLCLVKGEVIRSVAQIA
RHDV2-MW460157	GLPGFMRHNGSGWMIHIGNGMYISNTHTARSSCSEIVTCSPTTDLCLVKGEVIRSVAQIA
RHDV2-MW460158	GLPGFMRHNGSGWMIHIGNGMYISNTHTARSSCSEIVTCSPTTDLCLVKGEVIRSVAQIA
RHDV2-MW460159	GLPGFMRHNGSGWMIHIGNGMYISNTHTARSSCSEIVTCSPTTDLCLVKGEVIRSVAQIA
RHDV2-MW460161	GLPGFMRHNGSGWMIHIGNGMYISNTHTARSSCSEIVTCSPTTDLCLVKGEVIRSVAQIA
RHDV2-MW460165	GLPGFMRHNGSGWMIHIGNGMYISNTHTARSSCSEIVTCSPTTDLCLVKGEVIRSVAQIA
RHDV2-MW460173	GLPGFMRHNGSGWMIHIGNGMYISNTHTARSSCSEIVTCSPTTDLCLVKGEVIRSVAQIA
RHDV2-MW460175	GLPGFMRHNGSGWMIHIGNGMYISNTHTARSSCSEIVTCSPTTDLCLVKGEVIRSVAQIA
RHDV2-MW460177	GLPGFMRHNGSGWMIHIGNGMYISNTHTARSSCSEIVTCSPTTDLCLVKGEVIRSVAQIA
RHDV2-MW460179	GLPGFMRHNGSGWMIHIGNGMYISNTHTARSSCSEIVTCSPTTDLCLVKGEVIRSVAQIA
RHDV2-MW460180	GLPGFMRHNGSGWMIHIGNGMYISNTHTARSSCSEIVTCSPTTDLCLVKGEVIRSVAQIA
RHDV2-MW460183	GLPGFMRHNGSGWMIHIGNGMYISNTHTARSSCSEIVTCSPTTDLCLVKGEVIRSVAQIA
RHDV2-MW460186	GLPGFMRHNGSGWMIHIGNGMYISNTHTARSSCSEIVTCSPTTDLCLVKGEVIRSVAQIA
RHDV2-MW460187	GLPGFMRHNGSGWMIHIGNGMYISNTHTARSSCSEIVTCSPTTDLCLVKGEVIRSVAQIA
RHDV2-MW460190	GLPGFMRHNGSGWMIHIGNGMYISNTHTARSSCSEIVTCSPTTDLCLVKGEVIRSVAQIA
RHDV2-MW460193	GLPGFMRHNGSGWMIHIGNGMYISNTHTARSSCSEIVTCSPTTDLCLVKGEVIRSVAQIA
RHDV2-MW460194	GLPGFMRHNGSGWMIHIGNGMYISNTHTARSSCSEIVTCSPTTDLCLVKGEVIRSVAQIA
RHDV2-MW460195	GLPGFMRHNGSGWMIHIGNGMYISNTHTARSSCSEIVTCSPTTDLCLVKGEVIRSVAQIA
RHDV2-MW460197	GLPGFMRHNGSGWMIHIGNGMYISNTHTARSSCSEIVTCSPTTDLCLVKGEVIRSVAQIA
RHDV2-MW460198	GLPGFMRHNGSGWMIHIGNGMYISNTHTARSSCSEIVTCSPTTDLCLVKGEVIRSVAQIA
RHDV2-MW460202	GLPGFMRHNGSGWMIHIGNGMYISNTHTARSSCSEIVTCSPTTDLCLVKGEVIRSVAQIA
RHDV2-MW460204	GLPGFMRHNGSGWMIHIGNGMYISNTHTARSSCSEIVTCSPTTDLCLVKGEVIRSVAQIA
RHDV2-MW460208	GLPGFMRHNGSGWMIHIGNGMYISNTHTARSSCSEIVTCSPTTDLCLVKGEVIRSVAQIA
RHDV2-MW460210	GLPGFMRHNGSGWMIHIGNGMYISNTHTARSSCSEIVTCSPTTDLCLVKGEVIRSVAQIA
RHDV2-MW460212	GLPGFMRHNGSGWMIHIGNGMYISNTHTARSSCSEIVTCSPTTDLCLVKGEVIRSVAQIA
RHDV2-MW460213	GLPGFMRHNGSGWMIHIGNGMYISNTHTARSSCSEIVTCSPTTDLCLVKGEVIRSVAQIA
RHDV2-MW460214	GLPGFMRHNGSGWMIHIGNGMYISNTHTARSSCSEIVTCSPTTDLCLVKGEVIRSVAQIA
RHDV2-MW460217	GLPGFMRHNGSGWMIHIGNGMYISNTHTARSSCSEIVTCSPTTDLCLVKGEVIRSVAQIA
RHDV2-MW460220	GLPGFMRHNGSGWMIHIGNGMYISNTHTARSSCSEIVTCSPTTDLCLVKGEVIRSVAQIA
RHDV2-MW460223	GLPGFMRHNGSGWMIHIGNGMYISNTHTARSSCSEIVTCSPTTDLCLVKGEVIRSVAQIA
RHDV2-MW460224	GLPGFMRHNGSGWMIHIGNGMYISNTHTARSSCSEIVTCSPTTDLCLVKGEVIRSVAQIA
RHDV2-MW460226	GLPGFMRHNGSGWMIHIGNGMYISNTHTARSSCSEIVTCSPTTDLCLVKGEVIRSVAQIA
RHDV2-MW460227	GLPGFMRHNGSGWMIHIGNGMYISNTHTARSSCSEIVTCSPTTDLCLVKGEVIRSVAQIA
RHDV2-MW460232	GLPGFMRHNGSGWMIHIGNGMYISNTHTARSSCSEIVTCSPTTDLCLVKGEVIRSVAQIA
RHDV2-MW460233	GLPGFMRHNGSGWMIHIGNGMYISNTHTARSSCSEIVTCSPTTDLCLVKGEVIRSVAQIA
RCV-KX357707	GLPGFMRHNGSGWMIHIGNGMYISNTHTARSSCSEIVTCSPTTDLCLVKGEVIRSVAQIA
RCV-KX357655	GLPGFMRHNGSGWMIHIGNGMYISNTHTARSSCSEIVTCSPTTDLCLVKGEVIRSVAQIA
RCV-KX357696	GLPGFMRHNGSGWMIHIGNGMYISNTHTARSSCSEIVTCSPTTDLCLVKGEVIRSVAQIA
RCV-KX357658	GLPGFMRHNGSGWMIHIGNGMYISNTHTARSSCSEIVTCSPTTDLCLVKGEVIRSVAQIA
RCV-KX357663	GLPGFMRHNGSGWMIHIGNGMYISNTHTARSSCSEIVTCSPTTDLCLVKGEVIRSVAQIA

```
RCV-KX357703    GLPGFMRHNGSGWMIHIGNGMYISNTHTARSSCSEIVTCSPTTDLCLVKGEVIRSVAQIA
RCV-KX357705    GLPGFMRHNGSGWMIHIGNGMYISNTHTARSSCSEIVTCSPTTDLCLVKGEVIRSVAQIA
RCV-KX357697    GLPGFMRHNGSGWMIHIGNGMYISNTHTARSSCSEIVTCSPTTDLCLVKGEVIRSVAQIA
RCV-KX357700    GLPGFMRHNGSGWMIHIGNGMYISNTHTARSSCSEIVTCSPTTDLCLVKGEVIRSVAQIA
RHDV2-MW460153  GLPGFMRHNGSGWMIHIGNGMYISNTHTARSSCSEIVTCSPTTDLCLVKGEVIRSVAQIA
RHDV2-MW460155  GLPGFMRHNGSGWMIHIGNGMYISNTHTARSSCSEIITCSPTTDLCLVKGEVIRSVAQIA
RHDV2-MW460178  GLPGFMRHNGSGWMIHIGNGMYISNTHTARSSCSEIVTCSPTTDLCLVKGEVIRSVAQIA
RHDV2-MW460185  GLPGFMRHNGSGWMIHIGNGMYISNTHTARSSCSEIVTCSPTTDLCLVKGEVIRSVAQIA
RHDV2-MW460211  GLPGFMRHNGSGWMIHIGNGMYISNTHTARSSCSEIVTCSPTTDLCLVKGEVIRSVAQIA
RCV-KX357689    GLPGFMRHNGSGWMIHIGNGMYISNTHTARSSCSEIVTCSPTTDLCLVKGEVIRSVAQIA
RCV-KX357682    GLPGFMRHNGSGWMIHIGNGMYISNTHTARSSCSEIVTCSPTTDLCLVKGEVIRSVAQIA
RCV-KX357691    GLPGFMRHNGSGWMIHIGNGMYISNTHTARSSCSEIVTCSPTTDLCLVKGEVIRSVAQIA
RCV-KX357683    GLPGFMRHNGSGWMIHIGNGMYISNTHTARSSCSEIVTCSPTTDLCLVKGEVIRSVAQIA
RCV-KX357686    GLPGFMRHNGSGWMIHIGNGMYISNTHTARSSCSEIVTCSPTTDLCLVKGEVIRSVAQIA
RCV-KX357685    GLPGFMRHNGSGWMIHIGNGMYISNTHTARSSCSEIVTCSPTTDLCLVKGEVIRSVAQIA
RCV-KX357692    GLPGFMRHNGSGWMIHIGNGMYISNTHTARSSCSEIVTCSPTTDLCLVKGEVIRSVAQIA
RCV-KX357684    GLPGFMRHNGSGWMIHIGNGMYISNTHTARSSCSEIVTCSPTTDLCLVKGEVIRSVAQIA
RCV-KX357690    GLPGFMRHNGSGWMIHIGNGMYISNTHTARSSCSEIVTCSPTTDLCLVKGEVIRSVAQIA
RCV-KX357687    GLPGFMRHNGSGWMIHIGNGMYISNTHTARSSCSEIVTCSPTTDLCLVKGEVIRSVAQIA
RCV-KX357701    GLPGFMRHNGSGWMIHIGNGLYISNTHTARSSCSEIVTCSPTTDLCLVKGEVIRSVAQIA
RHDV2-MW460219  GLPGFMRHNGSGWMIHIGNGMYISNTHTARSSCSEIVTCSPTTDLCLVKGEVIKSVAQIA
RCV-KX357693    GLPGFMRHNGSGWMIHIGNGVYISNTHTARSSCSEIVTCSPTTDLCLVKGEVIRSVAQIA
RCV-EU871528    GLPGFMRHNGSGWMIHIGNGMYISNTHTARSSCSDIVTCSPTTDLCLVKGEVIRSVAQIA
RCV-KX357673    GLPGFMRHNGSGWMIHIGNGMYISNTHTARSSCSDIVTCSPTTDLCLVKGEVIRSVAQIA
RCV-KX357675    GLPGFMRHNGSGWMIHIGNGMYISNTHTARSSCSDIITCSPTTDLCLVKGEVIRSVAQIA
RCV-KX357694    GLPGFMRHNGSGWMIHIGNGMYISNTHTARSSCSDIITCSPTTDLCLVKGEIIRSVAQIA
RHDV2-MW460199  GLPGFMRHNGSGWMIHIGNGMYISNTHTARSSCSEIVTCSPTTDLCLVKGEAIRSVAQIA
RHDV2-MW460200  GLPGFMRHNGSGWMIHIGNGMYISNTHTARSSCSEIVTCSPTTDLCLVKGEAIRSVAQIA
RCV-KX357665    GLPGFMRHNGSGWMIHIGNGMYISNTHTARSSCSEIVTCSPTTDLCLVKGEAIRSVAQIA
RCV-KX357681    GLPGFMRHNGSGWMIHIGNGMYISNTHTARSSCSEIVTCSPTTDLCLVKGEAIRSVAQIA
RCV-KX357662    GLPGFMRHNGSGWMIHIGNGMYISNTHTARSSCSEIVTCSPTTDLCLVKGETIRSVAQIA
RCV-KX357661    GLPGFMRHNGSGWMIHIGNGMYISNTHTARSSCSEIVTCSPTTDLCLVKGETIRSVAQIA
RCV-KX357680    GLPGFMRHNGSGWMIHIGNGMYISNTHTARSSCSEIVTCSPTTDLCLVKGETIRSVAQIA
RCV-KX357679    GLPGFMRHNGSGWMIHIGNGMYISNTHTARSSCSEIVTCSPTTDLCLVKGETIRSVAQIA
RCV-KX357664    GLPGFMRHNGSGWMIHIGNGMYISNTHTARSSCSEIVTCSPTTDLCLVKGETIRSVAQIA
RCV-KX357674    GLPGFMRHNGSGWMIHIGNGMYISNTHTARSSCSEIVTCSPTTDLCLVKGETIRSVAQIA
RCV-KX357669    GLPGFMRHNGSGWMIHIGNGMYISNTHTARSSCSEIVTCSPTTDLCLVKGETIRSVAQIA
RCV-KX357666    GLPGFMRHNGSGWMIHIGNGMYISNTHTARSSCSEIVTCSPTTDLCLVKGETIRSVAQIA
RCV-KX357659    GLPGFMRHNGSGWMIHIGNGMYISNTHTARSSCSEIVTCSPTTDLCLVKGETIRSVAQIA
RCV-KX357676    GLPGFMRHNGSGWMIHIGNGMYISNTHTARSSCSEVVTCSPTTDLCLVKGETIRSVAQIA
RCV-KX357660    GLPGYMRHNGSGWMIHIGNGMYISNTHTARSSCSEIVTCSPTTDLCLVKGETIRSVAQIA
RCV-KX357656    GLPGFMRHNGSGWMIHIGNGMYISNTHTARSSCSEIVTCSPTTDLCLVKGEVIRSVAQIA
RHDV2-MW460150  GLPGFMRHNGSGWMIHIGSGMYISNTHTARSSCSEIVTYSPTTDLCLVKGEVIRSVAQIA
HaCV-MK138383   GLPGYMRFNGSGWMIHIGNGLYLSNTHTAKSSMSDIITCSPTTDLCLVKSEPIRSVAQIA
```

```
HaCV-MH204883   GLPGYLRFNGSGWMIHIGNGMYLSNTHTARSSCSDIVTCSPTTDLCLVKAEPIKSVAQIA
EBHSV-MK440616  GLPGYLRFNGSGWMIHIGNGLYLSNTHTARSSCSEIVTCSPTTDLCLVKAEPIRSVAQIA
EBHSV-MK440613  GLPGYLRFNGSGWMIHIGNGLYLSNTHTARSSCSEIVTCSPTTDLCLVKAEPIRSVAQIA
EBHSV-LR899171  GLPGYLRFNGSGWMIHIGNGLYLSNTHTARSSCSEIVTCSPTTDLCLVKAEPIRSVAQIA
EBHSV-LR899152  GLPGYLRFNGSGWMIHIGNGLYLSNTHTARSSCSEIVTCSPTTDLCLVKAEPIRSVAQIA
EBHSV-LR899140  GLPGYLRFNGSGWMIHIGNGLYLSNTHTARSSCSEIVTCSPTTDLCLVKAEPIRSVAQIA
EBHSV-LR899188  GLPGYLRFNGSGWMIHIGNGLYLSNTHTARSSCSEIVTCSPTTDLCLVKAEPIRSVAQIA
EBHSV-LR899185  GLPGYLRFNGSGWMIHIGNGLYLSNTHTARSSCSEIVTCSPTTDLCLVKAEPIRSVAQIA
EBHSV-LR899182  GLPGYLRFNGSGWMIHIGNGLYLSNTHTARSSCSEIVTCSPTTDLCLVKAEPIRSVAQIA
EBHSV-MK440614  GLPGYLRFNGSGWMIHIGNGLYLSNTHTARSSCSEIVTCSPTTDLCLVKAEPIRSVAQIA
EBHSV-MK440615  GLPGYLRFNGSGWMIHIGNGLYLSNTHTARSSCSEIVTCSPTTDLCLVKAEPIRSVAQIA
EBHSV-KC832838  GMPGYLRFNGSGWMIHIGNGMYLSNTHTARSSCSEIVTCSPTTDLCLVKAEPIRSVAQIA
EBHSV-KC832839  GLPGYLRFNGSGWMIHIGNGMYLSNTHTARSSCSEIVTCSPTTDLCLVKAEPIRSVAQIA
EBHSV-MK440617  GLPGYLRFNGSGWMIHIGNGMYLSNTHTARSSCSEIVTCSPTTDLCLVKAEPIRSVAQIA
EBHSV-Z69620    GLPGYLRFNGSGWMIHIGNGMYLSNTHTARSSCSEIVTCSPTTDLCLVKAEPIRSVAQIA
HaCV-KR230102   GLPVYLRFNGSGWMIHIGNGLYLSNTHTAHSSCSDIVTCSPTTDLCLVKSDPIRSVAQIA
HaCV-MK138384   GLPGYMKFNGSGWMIHIGNGLYLSNTHTARSSQSDIVTCSPTSDLCLVKSEPLRSVAQIA
HaCV-MK138385   GLPGYMKFNGSGWMIHIGNGMYLSNTHTARSSQSEIVTCSPTSDLCLVKSEPLRSVAQIA
                *:* :::.**** * ***.*:*:******:** *:::* **.:*****:.: ::*****:

RHDV2-MN901451  EGTPVSDWKKSPITTYGIKKTLSDSTKIDVLAYDGCTQTTHGDCGLPLYDPSGKIVAIHT
RHDV2-MN061492  EGTPVSDWKKSPITTYGIKKTLSDSTKIDVLAYDGCTQTTHGDCGLPLYDSSGKIVAIHT
RHDV2-MN738377  EGTPVSDWKKSPITTYGIKKTLSDSTKIDVLAYDGCTQTTHGDCGLPLYDSSGKIVAIHT
RHDV2-MN786321  EGTPVSDWKKSPITTYGIKKTLSDSTKIDVLAYDGCTQTTHGDCGLPLYDSSGKIVAIHT
RHDV2-MT506233  EGTPVSDWKKSPITTYGIKKTLSDSTKIDVLAYDGCTQTTHGDCGLPLYDSSGKIVAIHT
RHDV2-MT506234  EGTPVSDWKKSPITTYGIKKTLSDSTKIDVLAYDGCTQTTHGDCGLPLYDSSGKIVAIHT
RHDV2-MT506235  EGTPVSDWKKSPITTYGIKKTLSDSTKIDVLAYDGCTQTTHGDCGLPLYDSSGKIVAIHT
RHDV2-MT506236  EGTPVSDWKKSPITTYGIKKTLSDSTKIDVLAYDGCTQTTHGDCGLPLYDSSGKIVAIHT
RHDV2-MW926372  EGTPVSDWKKSPITTYGIKKTLSDSTKIDVLAYDGCTQTTHGDCGLPLYDSSGKIVAIHT
RHDV2-MW926373  EGTPVSDWKKSPITTYGIKKTLSDSTKIDVLAYDGCTQTTHGDCGLPLYDSSGKIVAIHT
RHDV2-MW926374  EGTPVSDWKKSPITTYGIKKTLSDSTKIDVLAYDGCTQTTHGDCGLPLYDSSGKIVAIHT
RHDV2-MW926375  EGTPVSDWKKSPITTYGIKKTLSDSTKIDVLAYDGCTQTTHGDCGLPLYDSSGKIVAIHT
RHDV2-MW926376  EGTPVSDWKKSPITTYGIKKTLSDSTKIDVLAYDGCTQTTHGDCGLPLYDSSGKIVAIHT
RHDV2-MW926377  EGTPVSDWKKSPITTYGIKKTLSDSTKIDVLAYDGCTQTTHGDCGLPLYDSSGKIVAIHT
RHDV2-MW926379  EGTPVSDWKKSPITTYGIKKTLSDSTKIDVLAYDGCTQTTHGDCGLPLYDSSGKIVAIHT
RHDV2-MW926380  EGTPVSDWKKSPITTYGIKKTLSDSTKIDVLAYDGCTQTTHGDCGLPLYDSSGKIVAIHT
RHDV2-MW926382  EGTPVSDWKKSPITTYGIKKTLSDSTKIDVLAYDGCTQTTHGDCGLPLYDSSGKIVAIHT
RHDV2-MW926383  EGTPVSDWKKSPITTYGIKKTLSDSTKIDVLAYDGCTQTTHGDCGLPLYDSSGKIVAIHT
RHDV2-MW926384  EGTPVSDWKKSPITTYGIKKTLSDSTKIDVLAYDGCTQTTHGDCGLPLYDSSGKIVAIHT
RHDV2-MW926371  EGTPVSDWKKSPITTYGIKKTLSDSTKIDVLAYDGCTQTTHGDCGLPLYDSSGKIVAIHT
RHDV2-MN853660  EGTPVSDWKKSPITTYGIKKTLSDSTKIDVLAYDGCTQTTHGDCGLPLYDSSGKIVAIHT
RHDV2-MN853661  EGTPVSDWKKSPITTYGIKKTLSDSTKIDVLAYDGCTQTTHGDCGLPLYDSSGKIVAIHT
RHDV2-MW926381  EGTPVSDWKKSPITTYGIKKTLSDSTKVDVLAYDGCTQTTHGDCGLPLYDSSGKIVAIHT
RHDV2-MN853659  EGTPVSDWKKSPITTYGIKKTLSDSTKIDVLAYDGCTQTTHGDCGLPLYDSSGKVVAIHT
```

```
RHDV2-MW926378  EGTPVSDWKKSPITTYGIKKTLSDSTKIDVLAYDGCTQTTHGDCGLPLYDSSGKIVAIHT
RHDV2-MG763942  EGTPVSDWKKSPITTYGIKKTLSDSTKIDVLAYDGCTQTTHGDCGLPLYDSSGKIVAIHT
RHDV2-MG763943  EGTPVSDWKKSPITTYGIKKTLSDSTKIDVLAYDGCTQTTHGDCGLPLYDSSGKIVAIHT
RHDV2-MG763945  EGTPVSDWKKSPITTYGIKKTLSDSTKIDVLAYDGCTQTTHGDCGLPLYDSSGKIVAIHT
RHDV2-MG763949  EGTPVSDWKKSPITTYGIKKTLSDSTKIDVLAYDGCTQTTHGDCGLPLYDSSGKIVAIHT
RHDV2-MG763950  EGTPVSDWKKSPITTYGIKKTLSDSTKIDVLAYDGCTQTTHGDCGLPLYDSSGKIVAIHT
RHDV2-MG763951  EGTPVSDWKKSPITTYGIKKTLSDSTKIDVLAYDGCTQTTHGDCGLPLYDSSGKIVAIHT
RHDV2-MF407653  EGTPVSDWKKSPITTYGIKKTLSDSTKIDVLAYDGCTQTTHGDCGLPLYDSSGKIVAIHT
RHDV2-MN737113  EGTPVSDWKKSPITTYGIKKTLSDSTKIDVLAYDGCTQTTHGDCGLPLYDSSGKIVAIHT
RHDV-MT628288   EGTPVSDWKKSPITTYGIKKTLSDSTKIDVLAYDGCTQTTHGDCGLPLYDSSGKIVAIHT
RHDV-MT628291   EGTPVSDWKKSPITTYGIKKTLSDSTKIDVLAYDGCTQTTHGDCGLPLYDSSGKIVAIHT
RCV-MN746289    EGTPVSDWKKSPITTYGIKKTLSDSTKIDVLAYDGCTQTTHGDCGLPLYDSSGKIVAIHT
RCV-MN746288    EGTPVSDWKKSPITTYGIKKTLSDSTKIDVLAYDGCTQTTHGDCGLPLYDSSGKIVAIHT
RCV-MN737117    EGTPVSDWKKSPITTYGIKKTLSDSTKIDVLAYDGCTQTTHGDCGLPLYDSSGKIVAIHT
RHDV2-MN737114  EGTPVSDWKKSPITTYGIKKTLSDSTKIDVLAYDGCTQTTHGDCGLPLYDSSGKIVAIHT
RHDV2-MW974834  EGTPVSDWKKSPITTYGIKKTLSDSTKIDVLAYDGCTQTTHGDCGLPLYDSSGKIVAIHT
RHDV2-MT586027  EGTPVSDWKKSPITTYGIKKTLSDSTKIDVLAYDGCTQTTHGDCGLPLYDSSGKIVAIHT
RHDV-MT628290   EGTPVSDWKKSPITTYGIKKTLSDSTKIDVLAYDGCTQTTHGDCGLPLYDSSGKIVAIHT
RCV-MN737115    EGTPVSDWKKSPITTYGIKKTLSDSTKIDVLAYDGCTQTTHGDCGLPLYDSCGKIVAIHT
RCV-MN737116    EGTPVSDWKKSPITTYGIKKTLSDSTKIDVLAYDGCTQTTHGGCGLPLYDSSGKIVAIHT
RHDV2-MK521927  EGTPVCDWKKSPISTYGIKKTLSDSTKIDVLAYDGCTQTTHGDCGLPLYDSSGKIVAIHT
RHDV2-MG602006  EGTPVCDWKKSPISTYGIKKTLSDSTKIDVLAYDGCTQTTHGDCGLPLYDSSGKIVAIHT
RHDV2-MG602007  EGTPVCDWKKSPISTYGIKKTLSDSTKIDVLAYDGCTQTTHGDCGLPLYDSSGKIVAIHT
RHDV2-MG763937  EGTPVCDWKKSPISTYGIKKTLSDSTKIDVLAYDGCTQTTHGDCGLPLYDSSGKIVAIHT
RHDV2-MG763938  EGTPVCDWKKSPISTYGIKKTLSDSTKIDVLAYDGCTQTTHGDCGLPLYDSSGKIVAIHT
RHDV2-MG763939  EGTPVCDWKKSPISTYGIKKTLSDSTKIDVLAYDGCTQTTHGDCGLPLYDSSGKIVAIHT
RHDV2-MG763940  EGTPVCDWKKSPISTYGIKKTLSDSTKIDVLAYDGCTQTTHGDCGLPLYDSSGKIVAIHT
RHDV2-MG763948  EGTPVCDWKKSPISTYGIKKTLSDSTKIDVLAYDGCTQTTHGDCGLPLYDSSGKIVAIHT
RHDV2-MG763953  EGTPVCDWKKSPISTYGIKKTLSDSTKIDVLAYDGCTQTTHGDCGLPLYDSSGKIVAIHT
RHDV2-MF407654  EGTPVCDWKKSPISTYGIKKTLSDSTKIDVLAYDGCTQTTHGDCGLPLYDSSGKIVAIHT
RHDV2-MF407656  EGTPVCDWKKSPISTYGIKKTLSDSTKIDVLAYDGCTQTTHGDCGLPLYDSSGKIVAIHT
RHDV2-MW460036  EGTPVCDWKKSPISTYGIKKTLSDSTKIDVLAYDGCTQTTHGDCGLPLYDSSGKIVAIHT
RHDV2-MW460101  EGTPVCDWKKSPISTYGIKKTLSDSTKIDVLAYDGCTQTTHGDCGLPLYDSSGKIVAIHT
RHDV2-MW460102  EGTPVCDWKKSPISTYGIKKTLSDSTKIDVLAYDGCTQTTHGDCGLPLYDSSGKIVAIHT
RHDV2-MW460103  EGTPVCDWKKSPISTYGIKKTLSDSTKIDVLAYDGCTQTTHGDCGLPLYDSSGKIVAIHT
RHDV2-MW460105  EGTPVCDWKKSPISTYGIKKTLSDSTKIDVLAYDGCTQTTHGDCGLPLYDSSGKIVAIHT
RHDV2-MW460106  EGTPVCDWKKSPISTYGIKKTLSDSTKIDVLAYDGCTQTTHGDCGLPLYDSSGKIVAIHT
RHDV2-MW460112  EGTPVCDWKKSPISTYGIKKTLSDSTKIDVLAYDGCTQTTHGDCGLPLYDSSGKIVAIHT
RHDV2-MW460113  EGTPVCDWKKSPISTYGIKKTLSDSTKIDVLAYDGCTQTTHGDCGLPLYDSSGKIVAIHT
RHDV2-MW460114  EGTPVCDWKKSPISTYGIKKTLSDSTKIDVLAYDGCTQTTHGDCGLPLYDSSGKIVAIHT
RHDV2-MW460115  EGTPVCDWKKSPISTYGIKKTLSDSTKIDVLAYDGCTQTTHGDCGLPLYDSSGKIVAIHT
RHDV2-MW460116  EGTPVCDWKKSPISTYGIKKTLSDSTKIDVLAYDGCTQTTHGDCGLPLYDSSGKIVAIHT
RHDV2-MW460126  EGTPVCDWKKSPISTYGIKKTLSDSTKIDVLAYDGCTQTTHGDCGLPLYDSSGKIVAIHT
RHDV2-MW460138  EGTPVCDWKKSPISTYGIKKTLSDSTKIDVLAYDGCTQTTHGDCGLPLYDSSGKIVAIHT
```

```
RHDV2-MW460145  EGTPVCDWKKSPISTYGIKKTLSDSTKIDVLAYDGCTQTTHGDCGLPLYDSSGKIVAIHT
RHDV2-MW460162  EGTPVCDWKKSPISTYGIKKTLSDSTKIDVLAYDGCTQTTHGDCGLPLYDSSGKIVAIHT
RHDV2-MW460172  EGTPVCDWKKSPISTYGIKKTLSDSTKIDVLAYDGCTQTTHGDCGLPLYDSSGKIVAIHT
RHDV2-MW460176  EGTPVCDWKKSPISTYGIKKTLSDSTKIDVLAYDGCTQTTHGDCGLPLYDSSGKIVAIHT
RHDV2-MW460182  EGTPVCDWKKSPISTYGIKKTLSDSTKIDVLAYDGCTQTTHGDCGLPLYDSSGKIVAIHT
RHDV2-MW460188  EGTPVCDWKKSPISTYGIKKTLSDSTKIDVLAYDGCTQTTHGDCGLPLYDSSGKIVAIHT
RHDV2-MW460189  EGTPVCDWKKSPISTYGIKKTLSDSTKIDVLAYDGCTQTTHGDCGLPLYDSSGKIVAIHT
RHDV2-MW460191  EGTPVCDWKKSPISTYGIKKTLSDSTKIDVLAYDGCTQTTHGDCGLPLYDSSGKIVAIHT
RHDV2-MW460203  EGTPVCDWKKSPISTYGIKKTLSDSTKIDVLAYDGCTQTTHGDCGLPLYDSSGKIVAIHT
RHDV2-MW460205  EGTPVCDWKKSPISTYGIKKTLSDSTKIDVLAYDGCTQTTHGDCGLPLYDSSGKIVAIHT
RHDV2-MW460206  EGTPVCDWKKSPISTYGIKKTLSDSTKIDVLAYDGCTQTTHGDCGLPLYDSSGKIVAIHT
RHDV2-MW460207  EGTPVCDWKKSPISTYGIKKTLSDSTKIDVLAYDGCTQTTHGDCGLPLYDSSGKIVAIHT
RHDV2-MW460215  EGTPVCDWKKSPISTYGIKKTLSDSTKIDVLAYDGCTQTTHGDCGLPLYDSSGKIVAIHT
RHDV2-MW460221  EGTPVCDWKKSPISTYGIKKTLSDSTKIDVLAYDGCTQTTHGDCGLPLYDSSGKIVAIHT
RHDV2-MW460222  EGTPVCDWKKSPISTYGIKKTLSDSTKIDVLAYDGCTQTTHGDCGLPLYDSSGKIVAIHT
RHDV2-MW460225  EGTPVCDWKKSPISTYGIKKTLSDSTKIDVLAYDGCTQTTHGDCGLPLYDSSGKIVAIHT
RHDV2-MW467791  EGTPVCDWKKSPISTYGIKKTLSDSTKIDVLAYDGCTQTTHGDCGLPLYDSSGKIVAIHT
RHDV2-MF421574  EGTPVCDWKKSPISTYGIKKTLSDSTKIDVLAYDGCTQTTHGDCGLPLYDSSGKIVAIHT
RHDV2-MF421576  EGTPVCDWKKSPISTYGIKKTLSDSTKIDVLAYDGCTQTTHGDCGLPLYDSSGKIVAIHT
RHDV2-MF421577  EGTPVCDWKKSPISTYGIKKTLSDSTKIDVLAYDGCTQTTHGDCGLPLYDSSGKIVAIHT
RHDV2-MF421579  EGTPVCDWKKSPISTYGIKKTLSDSTKIDVLAYDGCTQTTHGDCGLPLYDSSGKIVAIHT
RHDV2-MF421583  EGTPVCDWKKSPISTYGIKKTLSDSTKIDVLAYDGCTQTTHGDCGLPLYDSSGKIVAIHT
RHDV2-MF421584  EGTPVCDWKKSPISTYGIKKTLSDSTKIDVLAYDGCTQTTHGDCGLPLYDSSGKIVAIHT
RHDV2-MF421585  EGTPVCDWKKSPISTYGIKKTLSDSTKIDVLAYDGCTQTTHGDCGLPLYDSSGKIVAIHT
RHDV2-MF421586  EGTPVCDWKKSPISTYGIKKTLSDSTKIDVLAYDGCTQTTHGDCGLPLYDSSGKIVAIHT
RHDV2-MF421587  EGTPVCDWKKSPISTYGIKKTLSDSTKIDVLAYDGCTQTTHGDCGLPLYDSSGKIVAIHT
RHDV2-MF421589  EGTPVCDWKKSPISTYGIKKTLSDSTKIDVLAYDGCTQTTHGDCGLPLYDSSGKIVAIHT
RHDV2-MF421590  EGTPVCDWKKSPISTYGIKKTLSDSTKIDVLAYDGCTQTTHGDCGLPLYDSSGKIVAIHT
RHDV2-MF421591  EGTPVCDWKKSPISTYGIKKTLSDSTKIDVLAYDGCTQTTHGDCGLPLYDSSGKIVAIHT
RHDV2-MF421592  EGTPVCDWKKSPISTYGIKKTLSDSTKIDVLAYDGCTQTTHGDCGLPLYDSSGKIVAIHT
RHDV2-MF421593  EGTPVCDWKKSPISTYGIKKTLSDSTKIDVLAYDGCTQTTHGDCGLPLYDSSGKIVAIHT
RHDV2-MF421594  EGTPVCDWKKSPISTYGIKKTLSDSTKIDVLAYDGCTQTTHGDCGLPLYDSSGKIVAIHT
RHDV2-MF421595  EGTPVCDWKKSPISTYGIKKTLSDSTKIDVLAYDGCTQTTHGDCGLPLYDSSGKIVAIHT
RHDV2-MF421596  EGTPVCDWKKSPISTYGIKKTLSDSTKIDVLAYDGCTQTTHGDCGLPLYDSSGKIVAIHT
RHDV2-MF421597  EGTPVCDWKKSPISTYGIKKTLSDSTKIDVLAYDGCTQTTHGDCGLPLYDSSGKIVAIHT
RHDV2-MF421598  EGTPVCDWKKSPISTYGIKKTLSDSTKIDVLAYDGCTQTTHGDCGLPLYDSSGKIVAIHT
RHDV2-MF421600  EGTPVCDWKKSPISTYGIKKTLSDSTKIDVLAYDGCTQTTHGDCGLPLYDSSGKIVAIHT
RHDV2-MF421601  EGTPVCDWKKSPISTYGIKKTLSDSTKIDVLAYDGCTQTTHGDCGLPLYDSSGKIVAIHT
RHDV2-MF421602  EGTPVCDWKKSPISTYGIKKTLSDSTKIDVLAYDGCTQTTHGDCGLPLYDSSGKIVAIHT
RHDV2-MF421603  EGTPVCDWKKSPISTYGIKKTLSDSTKIDVLAYDGCTQTTHGDCGLPLYDSSGKIVAIHT
RHDV2-MF421604  EGTPVCDWKKSPISTYGIKKTLSDSTKIDVLAYDGCTQTTHGDCGLPLYDSSGKIVAIHT
RHDV2-MF421606  EGTPVCDWKKSPISTYGIKKTLSDSTKIDVLAYDGCTQTTHGDCGLPLYDSSGKIVAIHT
RHDV2-MF421607  EGTPVCDWKKSPISTYGIKKTLSDSTKIDVLAYDGCTQTTHGDCGLPLYDSSGKIVAIHT
RHDV2-MF421608  EGTPVCDWKKSPISTYGIKKTLSDSTKIDVLAYDGCTQTTHGDCGLPLYDSSGKIVAIHT
```

```
RHDV2-MF421609  EGTPVCDWKKSPISTYGIKKTLSDSTKIDVLAYDGCTQTTHGDCGLPLYDSSGKIVAIHT
RHDV2-MF421610  EGTPVCDWKKSPISTYGIKKTLSDSTKIDVLAYDGCTQTTHGDCGLPLYDSSGKIVAIHT
RHDV2-MF421611  EGTPVCDWKKSPISTYGIKKTLSDSTKIDVLAYDGCTQTTHGDCGLPLYDSSGKIVAIHT
RHDV2-MF421612  EGTPVCDWKKSPISTYGIKKTLSDSTKIDVLAYDGCTQTTHGDCGLPLYDSSGKIVAIHT
RHDV2-MF421613  EGTPVCDWKKSPISTYGIKKTLSDSTKIDVLAYDGCTQTTHGDCGLPLYDSSGKIVAIHT
RHDV2-MF421615  EGTPVCDWKKSPISTYGIKKTLSDSTKIDVLAYDGCTQTTHGDCGLPLYDSSGKIVAIHT
RHDV2-MF421617  EGTPVCDWKKSPISTYGIKKTLSDSTKIDVLAYDGCTQTTHGDCGLPLYDSSGKIVAIHT
RHDV2-MF421618  EGTPVCDWKKSPISTYGIKKTLSDSTKIDVLAYDGCTQTTHGDCGLPLYDSSGKIVAIHT
RHDV2-MF421620  EGTPVCDWKKSPISTYGIKKTLSDSTKIDVLAYDGCTQTTHGDCGLPLYDSSGKIVAIHT
RHDV2-MF421624  EGTPVCDWKKSPISTYGIKKTLSDSTKIDVLAYDGCTQTTHGDCGLPLYDSSGKIVAIHT
RHDV2-MF421625  EGTPVCDWKKSPISTYGIKKTLSDSTKIDVLAYDGCTQTTHGDCGLPLYDSSGKIVAIHT
RHDV2-MF421627  EGTPVCDWKKSPISTYGIKKTLSDSTKIDVLAYDGCTQTTHGDCGLPLYDSSGKIVAIHT
RHDV2-MF421636  EGTPVCDWKKSPISTYGIKKTLSDSTKIDVLAYDGCTQTTHGDCGLPLYDSSGKIVAIHT
RHDV2-MF421637  EGTPVCDWKKSPISTYGIKKTLSDSTKIDVLAYDGCTQTTHGDCGLPLYDSSGKIVAIHT
RHDV2-MF421638  EGTPVCDWKKSPISTYGIKKTLSDSTKIDVLAYDGCTQTTHGDCGLPLYDSSGKIVAIHT
RHDV2-MF421639  EGTPVCDWKKSPISTYGIKKTLSDSTKIDVLAYDGCTQTTHGDCGLPLYDSSGKIVAIHT
RHDV2-MF421642  EGTPVCDWKKSPISTYGIKKTLSDSTKIDVLAYDGCTQTTHGDCGLPLYDSSGKIVAIHT
RHDV2-MF421643  EGTPVCDWKKSPISTYGIKKTLSDSTKIDVLAYDGCTQTTHGDCGLPLYDSSGKIVAIHT
RHDV2-MF421645  EGTPVCDWKKSPISTYGIKKTLSDSTKIDVLAYDGCTQTTHGDCGLPLYDSSGKIVAIHT
RHDV2-MF421646  EGTPVCDWKKSPISTYGIKKTLSDSTKIDVLAYDGCTQTTHGDCGLPLYDSSGKIVAIHT
RHDV2-MF421647  EGTPVCDWKKSPISTYGIKKTLSDSTKIDVLAYDGCTQTTHGDCGLPLYDSSGKIVAIHT
RHDV2-MF421648  EGTPVCDWKKSPISTYGIKKTLSDSTKIDVLAYDGCTQTTHGDCGLPLYDSSGKIVAIHT
RHDV2-MF421649  EGTPVCDWKKSPISTYGIKKTLSDSTKIDVLAYDGCTQTTHGDCGLPLYDSSGKIVAIHT
RHDV2-MF421650  EGTPVCDWKKSPISTYGIKKTLSDSTKIDVLAYDGCTQTTHGDCGLPLYDSSGKIVAIHT
RHDV2-MF421651  EGTPVCDWKKSPISTYGIKKTLSDSTKIDVLAYDGCTQTTHGDCGLPLYDSSGKIVAIHT
RHDV2-MF421652  EGTPVCDWKKSPISTYGIKKTLSDSTKIDVLAYDGCTQTTHGDCGLPLYDSSGKIVAIHT
RHDV2-MF421654  EGTPVCDWKKSPISTYGIKKTLSDSTKIDVLAYDGCTQTTHGDCGLPLYDSSGKIVAIHT
RHDV2-MF421655  EGTPVCDWKKSPISTYGIKKTLSDSTKIDVLAYDGCTQTTHGDCGLPLYDSSGKIVAIHT
RHDV2-MF421656  EGTPVCDWKKSPISTYGIKKTLSDSTKIDVLAYDGCTQTTHGDCGLPLYDSSGKIVAIHT
RHDV2-MF421658  EGTPVCDWKKSPISTYGIKKTLSDSTKIDVLAYDGCTQTTHGDCGLPLYDSSGKIVAIHT
RHDV2-MF421659  EGTPVCDWKKSPISTYGIKKTLSDSTKIDVLAYDGCTQTTHGDCGLPLYDSSGKIVAIHT
RHDV2-MF421660  EGTPVCDWKKSPISTYGIKKTLSDSTKIDVLAYDGCTQTTHGDCGLPLYDSSGKIVAIHT
RHDV2-MF421661  EGTPVCDWKKSPISTYGIKKTLSDSTKIDVLAYDGCTQTTHGDCGLPLYDSSGKIVAIHT
RHDV2-MF421663  EGTPVCDWKKSPISTYGIKKTLSDSTKIDVLAYDGCTQTTHGDCGLPLYDSSGKIVAIHT
RHDV2-MF421664  EGTPVCDWKKSPISTYGIKKTLSDSTKIDVLAYDGCTQTTHGDCGLPLYDSSGKIVAIHT
RHDV2-MF421665  EGTPVCDWKKSPISTYGIKKTLSDSTKIDVLAYDGCTQTTHGDCGLPLYDSSGKIVAIHT
RHDV2-MF421666  EGTPVCDWKKSPISTYGIKKTLSDSTKIDVLAYDGCTQTTHGDCGLPLYDSSGKIVAIHT
RHDV2-MF421667  EGTPVCDWKKSPISTYGIKKTLSDSTKIDVLAYDGCTQTTHGDCGLPLYDSSGKIVAIHT
RHDV2-MF421668  EGTPVCDWKKSPISTYGIKKTLSDSTKIDVLAYDGCTQTTHGDCGLPLYDSSGKIVAIHT
RHDV2-MF421669  EGTPVCDWKKSPISTYGIKKTLSDSTKIDVLAYDGCTQTTHGDCGLPLYDSSGKIVAIHT
RHDV2-MF421670  EGTPVCDWKKSPISTYGIKKTLSDSTKIDVLAYDGCTQTTHGDCGLPLYDSSGKIVAIHT
RHDV2-MF421673  EGTPVCDWKKSPISTYGIKKTLSDSTKIDVLAYDGCTQTTHGDCGLPLYDSSGKIVAIHT
RHDV2-MF421674  EGTPVCDWKKSPISTYGIKKTLSDSTKIDVLAYDGCTQTTHGDCGLPLYDSSGKIVAIHT
RHDV2-MF421675  EGTPVCDWKKSPISTYGIKKTLSDSTKIDVLAYDGCTQTTHGDCGLPLYDSSGKIVAIHT
```

RHDV2-MF421676	EGTPVCDWKKSPISTYGIKKTLSDSTKIDVLAYDGCTQTTHGDCGLPLYDSSGKIVAIHT
RHDV2-MF421678	EGTPVCDWKKSPISTYGIKKTLSDSTKIDVLAYDGCTQTTHGDCGLPLYDSSGKIVAIHT
RHDV2-MF421680	EGTPVCDWKKSPISTYGIKKTLSDSTKIDVLAYDGCTQTTHGDCGLPLYDSSGKIVAIHT
RHDV2-MF421681	EGTPVCDWKKSPISTYGIKKTLSDSTKIDVLAYDGCTQTTHGDCGLPLYDSSGKIVAIHT
RHDV2-MF421682	EGTPVCDWKKSPISTYGIKKTLSDSTKIDVLAYDGCTQTTHGDCGLPLYDSSGKIVAIHT
RHDV2-MF421683	EGTPVCDWKKSPISTYGIKKTLSDSTKIDVLAYDGCTQTTHGDCGLPLYDSSGKIVAIHT
RHDV2-MF421684	EGTPVCDWKKSPISTYGIKKTLSDSTKIDVLAYDGCTQTTHGDCGLPLYDSSGKIVAIHT
RHDV2-MF421685	EGTPVCDWKKSPISTYGIKKTLSDSTKIDVLAYDGCTQTTHGDCGLPLYDSSGKIVAIHT
RHDV2-MF421689	EGTPVCDWKKSPISTYGIKKTLSDSTKIDVLAYDGCTQTTHGDCGLPLYDSSGKIVAIHT
RHDV2-MF421691	EGTPVCDWKKSPISTYGIKKTLSDSTKIDVLAYDGCTQTTHGDCGLPLYDSSGKIVAIHT
RHDV2-MF421694	EGTPVCDWKKSPISTYGIKKTLSDSTKIDVLAYDGCTQTTHGDCGLPLYDSSGKIVAIHT
RHDV2-MF421695	EGTPVCDWKKSPISTYGIKKTLSDSTKIDVLAYDGCTQTTHGDCGLPLYDSSGKIVAIHT
RHDV2-MF421696	EGTPVCDWKKSPISTYGIKKTLSDSTKIDVLAYDGCTQTTHGDCGLPLYDSSGKIVAIHT
RHDV2-MG763936	EGTPVCDWKKSPISTYGIKKTLSDSTKIDVLAYDGCTQTTHGDCGLPLYDSSGKIVAIHT
RHDV2-MG763947	EGTPVCDWKKSPISTYGIKKTLSDSTKIDVLAYDGCTQTTHGDCGLPLYDSSGKIVAIHT
RHDV2-MW460124	EGTPVCDWKKSPISTYGIKKTLSDSTKIDVLAYDGCTQTTHGDCGLPLYDSSGKIVAIHT
RHDV2-MW460125	EGTPVCDWKKSPISTYGIKKTLSDSTKIDVLAYDGCTQTTHGDCGLPLYDSSGKIVAIHT
RHDV2-MW460129	EGTPVCDWKKSPISTYGIKKTLSDSTKIDVLAYDGCTQTTHGDCGLPLYDSSGKIVAIHT
RHDV2-MW460147	EGTPVCDWKKSPISTYGIKKTLSDSTKIDVLAYDGCTQTTHGDCGLPLYDSSGKIVAIHT
RHDV2-MW460148	EGTPVCDWKKSPISTYGIKKTLSDSTKIDVLAYDGCTQTTHGDCGLPLYDSSGKIVAIHT
RHDV2-MW460231	EGTPVCDWKKSPISTYGIKKTLSDSTKIDVLAYDGCTQTTHGDCGLPLYDSSGKIVAIHT
RHDV2-MW460234	EGTPVCDWKKSPISTYGIKKTLSDSTKIDVLAYDGCTQTTHGDCGLPLYDSSGKIVAIHT
RHDV2-MW460237	EGTPVCDWKKSPISTYGIKKTLSDSTKIDVLAYDGCTQTTHGDCGLPLYDSSGKIVAIHT
RHDV2-MW460240	EGTPVCDWKKSPISTYGIKKTLSDSTKIDVLAYDGCTQTTHGDCGLPLYDSSGKIVAIHT
RHDV2-MF421628	EGTPVCDWKKSPISTYGIKKTLSDSTKIDVLAYDGCTQTTHGDCGLPLYDSSGKIVAIHT
RHDV2-MF421629	EGTPVCDWKKSPISTYGIKKTLSDSTKIDVLAYDGCTQTTHGDCGLPLYDSSGKIVAIHT
RHDV2-MF421630	EGTPVCDWKKSPISTYGIKKTLSDSTKIDVLAYDGCTQTTHGDCGLPLYDSSGKIVAIHT
RHDV2-MF421631	EGTPVCDWKKSPISTYGIKKTLSDSTKIDVLAYDGCTQTTHGDCGLPLYDSSGKIVAIHT
RHDV2-MF421633	EGTPVCDWKKSPISTYGIKKTLSDSTKIDVLAYDGCTQTTHGDCGLPLYDSSGKIVAIHT
RHDV2-MF421634	EGTPVCDWKKSPISTYGIKKTLSDSTKIDVLAYDGCTQTTHGDCGLPLYDSSGKIVAIHT
RHDV2-MF421635	EGTPVCDWKKSPISTYGIKKTLSDSTKIDVLAYDGCTQTTHGDCGLPLYDSSGKIVAIHT
RHDV2-MF421697	EGTPVCDWKKSPISTYGIKKTLSDSTKIDVLAYDGCTQTTHGDCGLPLYDSSGKIVAIHT
RHDV2-MF421698	EGTPVCDWKKSPISTYGIKKTLSDSTKIDVLAYDGCTQTTHGDCGLPLYDSSGKIVAIHT
RHDV2-MF421699	EGTPVCDWKKSPISTYGIKKTLSDSTKIDVLAYDGCTQTTHGDCGLPLYDSSGKIVAIHT
RHDV2-MF421605	EGTPVCDWKKSPISTYGIKKTLSDSTKIDVLAYDGCTQTTHGDCGLPLYDSSGKIVAIHT
RHDV2-MN737112	EGTPVCDWKKSPISTYGIKKTLSDSTKIDVLAYDGCTQTTHGDCGLPLYDSSGKIVAIHT
RHDV2-MF421614	EGTPVCDWKKSPISTYGIKKTLSDSTKIDVLAYDGCTQTTHGDCGLPLYDSSGKIVAIHT
RHDV2-MF421657	EGTPVCDWKKSPISTYGIKKTLSDSTKIDVLAYDGCTQTTHGDCGLPLYDSSGKIVAIHT
RHDV2-MF421671	EGTPVCDWKKSPISTYGIKKTLSDSTKIDVLAYDGCTQTTHGDCGLPLYDSSGKIVAIHT
RHDV2-MW460108	EGTPVCDWKKSPISTYGIKKTLSDSTKIDVLAYDGCTQTTHGDCGLPLYDSSGKIVAIHT
RHDV2-MW460109	EGTPVCDWKKSPISTYGIKKTLSDSTKIDVLAYDGCTQTTHGDCGLPLYDSSGKIVAIHT
RHDV2-MW460201	EGTPVCDWKKSPISTYGIKKTLSDSTKIDVLAYDGCTQTTHGDCGLPLYDSSGKIVAIHT
RHDV2-MF421575	EGTPVCDWKKSPISTYGIKKTLSDSTKIDVLAYDGCTQTTHGDCGLPLYDSSGKIVAIHT
RHDV2-MF421580	EGTPVCDWKKSPISTYGIKKTLSDSTKIDVLAYDGCTQTTHGDCGLPLYDSSGKIVAIHT

```
RHDV2-MF421581  EGTPVCDWKKSPISTYGIKKTLSDSTKIDVLAYDGCTQTTHGDCGLPLYDSSGKIVAIHT
RHDV2-MF421582  EGTPVCDWKKSPISTYGIKKTLSDSTKIDVLAYDGCTQTTHGDCGLPLYDSSGKIVAIHT
RHDV2-MF421672  EGTPVCDWKKSPISTYGIKKTLSDSTKIDVLAYDGCTQTTHGDCGLPLYDSSGKIVAIHT
RHDV2-MW460164  EGTPVCDWKKSPISTYGIKKTLSDSTKIDVLAYDGCTQTTHGDCGLPLYDSSGKIVAIHT
RHDV2-MW460174  EGTPVCDWKKSPISTYGIKKTLSDSTKIDVLAYDGCTQTTHGDCGLPLYDSSGKIVAIHT
RHDV2-MF421619  EGTPVCDWKKSPISTYGIKKTLSDSTKIDVLAYDGCTQTTHGDCGLPLYDSSGKIVAIHT
RHDV2-MW460168  EGTPVCDWKKSPISTYGVKKTLSDSTKIDVLAYDGCTQTTHGDCGLPLYDSSGKIVAIHT
RHDV2-MW460171  EGTPVCDWKKSPISTYGVKKTLSDSTKIDVLAYDGCTQTTHGDCGLPLYDSSGKIVAIHT
RHDV2-MW460229  EGTPVCDWKKSPISTYGIKKTLSDSTKIDVLAYDGCTQTTHGDCGLPLYDSSGKIVAIHT
RHDV2-MF421616  EGTPVCDWKKSPISTYGIKKTLSDSTKIDVLAYDGCTQTTHGDCGLPLYDSSGKIVAIHT
RHDV2-MF421621  EGTPVCDWKKSPISTYGIKKTLSDSTKIDVLAYDGCTQTTHGDCGLPLYDSSGKIVAIHT
RHDV2-MF421623  EGTPVCDWKKSPISTYGIKKTLSDSTKIDVLAYDGCTQTTHGDCGLPLYDSSGKIVAIHT
RHDV2-MF421686  EGTPVCDWKKSPISTYGIKKTLSDSTKIDVLAYDGCTQTTHGDCGLPLYDSSGKIVAIHT
RHDV2-MF421687  EGTPVCDWKKSPISTYGIKKTLSDSTKIDVLAYDGCTQTTHGDCGLPLYDSSGKIVAIHT
RHDV2-MF421688  EGTPVCDWKKSPISTYGIKKTLSDSTKIDVLAYDGCTQTTHGDCGLPLYDSSGKIVAIHT
RHDV2-MF421588  EGTPVCDWRKSPISTYGIKKTLSDSTKIDVLAYDGCTQTTHGDCGLPLYDSSGKIVAIHT
RHDV2-MF421692  EGTPVCDWKKSPISTYGIKRTLSDSTKIDVLAYDGCTQTTHGDCGLPLYDSSGKIVAIHT
RHDV2-MF421640  EGTPVCDWKKSPISTYGIKKTLSDSTKIDVLAYDGCTQTTHGDCGLPLYDSSGKIVAIHT
RHDV2-MF421641  EGTPVCDWKKSPISTYGIKKTLSDSTKIDVLAYDGCTQTTHGDCGLPLYDSSGKIVAIHT
RHDV2-MF421653  EGTPVCDWKKSPISTYGIKKTLSDSTKIDVLAYDGCTQTTHGDCGLPLYDSSGKIVAIHT
RHDV2-MF421677  EGTPVCDWKKSPISTYGIKKTLSDSTKIDVLAYDGCTQTTHGDCGLPLYDSSGKIVAIHT
RHDV2-MF421679  EGTPVCDWKKSPISTYGIKKTLSDSTKIDVLAYDGCTQTTHGDCGLPLYDSSGKIVAIHT
RHDV2-MF421693  EGTPVCDWKKSPISTYGIKKTLSDSTKIDVLAYDGCTQTTHGDCGLPLYDSSGKIVAIHT
RHDV2-MF421644  EGTPVCDWKKSPISTYGIKKTLSDSTKIDVLSYDGCTQTTHGDCGLPLYDSSGKIVAIHT
RHDV2-MF421632  EGTPVCDWKKSPISTYGIKKTLSDSTRIDVLAYDGCTQTTHGDCGLPLYDSSGKIVAIHT
RHDV2-MF421700  EGTPVCDWKKSPISTYGIKKTLSDSTRIDVLAYDGCTQTTHGDCGLPLYDSSGKIVAIHT
RHDV2-MW460132  EGTPVCDWKKSPISTYGIKKTLSDSTKIEVLAYDGCTQTTHGDCGLPLYDSSGKIVAIHT
RHDV2-MW460133  EGTPVCDWKKSPISTYGIKKTLSDSTKIEVLAYDGCTQTTHGDCGLPLYDSSGKIVAIHT
RHDV2-MW460134  EGTPVCDWKKSPISTYGIKKTLSDSTKIEVLAYDGCTQTTHGDCGLPLYDSSGKIVAIHT
RHDV2-MW460143  EGTPVCDWKKSPISTYGIKKTLSDSTKIEVLAYDGCTQTTHGDCGLPLYDSSGKIVAIHT
RHDV2-MF407651  EGTPVCDWKKSPISTYGIKKTLSDSTKIDVLAYDGCTQTTHGDCGLPLYDSSGKIVAIHT
RHDV2-MF407652  EGTPVCDWKKSPISTYGIKKTLSDSTKIDVLAYDGCTQTTHGDCGLPLYDSSGKIVAIHT
RHDV2-MF421567  EGTPVCDWKKSPISTYGIKKTLSDSTKIDVLAYDGCTQTTHGDCGLPLYDSSGKIVAIHT
RHDV2-MF421568  EGTPVCDWKKSPISTYGIKKTLSDSTKIDVLAYDGCTQTTHGDCGLPLYDSSGKIVAIHT
RHDV2-MF421571  EGTPVCDWKKSPISTYGIKKTLSDSTKIDVLAYDGCTQTTHGDCGLPLYDSSGKIVAIHT
RHDV2-MF421572  EGTPVCDWKKSPISTYGIKKTLSDSTKIDVLAYDGCTQTTHGDCGLPLYDSSGKIVAIHT
RHDV2-MF421573  EGTPVCDWKKSPISTYGIKKTLSDSTKIDVLAYDGCTQTTHGDCGLPLYDSSGKIVAIHT
RHDV-MH190418   EGTPVCDWKKSPISTYGIKKTLSDSTKIDVLAYDGCTQTTHGDCGLPLYDSSGKIVAIHT
RHDV-EU003579   EGTPVCDWKKSPISTYGIKKTLSDSTKIDVLAYDGCTQTTHGDCGLPLYDSSGKIVAIHT
RHDV-EU003580   EGTPVCDWKKSPISTYGIKKTLSDSTKIDVLAYDGCTQTTHGDCGLPLYDSSGKIVAIHT
RHDV-KP144789   EGTPVCDWKKSPISTYGIKKTLSDSTKIDVLAYDGCTQTTHGDCGLPLYDSSGKIVAIHT
RHDV-KU882093   EGTPVCDWKKSPISTYGIKKTLSDSTKIDVLAYDGCTQTTHGDCGLPLYDSSGKIVAIHT
RHDV-KU882092   EGTPVCDWKKSPISTYGIKKTLSDSTKIDVLAYDGCTQTTHGDCGLPLYDSSGKIVAIHT
RHDV-X87607     EGTPVCDWKKSPISTYGIKKTLSDSTKIDVLAYDGCTQTTHGDCGLPLYDSSGKIVAIHT
```

RHDV-Z29514	EGTPVCDWKKSPISTYGIKKTLSDSTKIDVLAYDGCTQTTHGDCGLPLYDSSGKIVAIHT
RHDV-M67473	EGTPVCDWKKSPISTYGIKKTLSDSTKIDVLAYDGCTQTTHGDCGLPLYDSSGKIVAIHT
RHDV-MG602005	EGTPVCDWKKSPITTYGIKKTLSDSTKIDVLAYDGCTQTTHGDCGLPLYDSSGKIVAIHT
RHDV-KY679902	EGTPVCDWKKSPISTYGIKKTLSDSTKIDVLAYDGCTQTTHGDCGLPLYDSSGKIVAIHT
RHDV-KY319031	EGTPVCDWKKSPISTYGIKKTLSDSTKIDVLAYDGCTQTTHGDCGLPLYDSSGKIVAIHT
RHDV-KY319032	EGTPVCDWKKSPISTYGIKKTLSDSTKIDVLAYDGCTQTTHGDCGLPLYDSSGKIVAIHT
RHDV-KY319034	EGTPVCDWKKSPISTYGIKKTLSDSTKIDVLAYDGCTQTTHGDCGLPLYDSSGKIVAIHT
RHDV-KY319035	EGTPVCDWKKSPISTYGIKKTLSDSTKIDVLAYDGCTQTTHGDCGLPLYDSSGKIVAIHT
RHDV-KF677011	EGTPVCDWKKSPISTYGIKKTLSDSTKIDVLAYDGCTQTTHGDCGLPLYDSSGKIVAIHT
RHDV-EU003578	EGTPVCDWKKSPISTYGIKKTLSDSTKIDVLAYDGCTQTTHGDCGLPLYDSSGKIVAIHT
RHDV-EU003582	EGTPVCDWKKSPISTYGIKKTLSDSTKIDVLAYDGCTQTTHGDCGLPLYDSSGKIVAIHT
RHDV-MW460041	EGTPVCDWKKSPISTYGIKKTLSDSTKIDVLAYDGCTQTTHGDCGLPLYDSSGKIVAIHT
RHDV-MW460063	EGTPVCDWKKSPISTYGIKKTLSDSTKIDVLAYDGCTQTTHGDCGLPLYDSSGKIVAIHT
RHDV-MW460075	EGTPVCDWKKSPISTYGIKKTLSDSTKIDVLAYDGCTQTTHGDCGLPLYDSSGKIVAIHT
RHDV-MW460094	EGTPVCDWKKSPISTYGIKKTLSDSTKIDVLAYDGCTQTTHGDCGLPLYDSSGKIVAIHT
RHDV-MW460104	EGTPVCDWKKSPISTYGIKKTLSDSTKIDVLAYDGCTQTTHGDCGLPLYDSSGKIVAIHT
RHDV-MW460139	EGTPVCDWKKSPISTYGIKKTLSDSTKIDVLAYDGCTQTTHGDCGLPLYDSSGKIVAIHT
RHDV-MW460154	EGTPVCDWKKSPISTYGIKKTLSDSTKIDVLAYDGCTQTTHGDCGLPLYDSSGKIVAIHT
RHDV-MW460184	EGTPVCDWKKSPISTYGIKKTLSDSTKIDVLAYDGCTQTTHGDCGLPLYDSSGKIVAIHT
RHDV-MW460196	EGTPVCDWKKSPISTYGIKKTLSDSTKIDVLAYDGCTQTTHGDCGLPLYDSSGKIVAIHT
RHDV-MW460209	EGTPVCDWKKSPISTYGIKKTLSDSTKIDVLAYDGCTQTTHGDCGLPLYDSSGKIVAIHT
RHDV-MW460216	EGTPVCDWKKSPISTYGIKKTLSDSTKIDVLAYDGCTQTTHGDCGLPLYDSSGKIVAIHT
RHDV-MW460235	EGTPVCDWKKSPISTYGIKKTLSDSTKIDVLAYDGCTQTTHGDCGLPLYDSSGKIVAIHT
RHDV-MW460239	EGTPVCDWKKSPISTYGIKKTLSDSTKIDVLAYDGCTQTTHGDCGLPLYDSSGKIVAIHT
RHDV-MW460241	EGTPVCDWKKSPISTYGIKKTLSDSTKIDVLAYDGCTQTTHGDCGLPLYDSSGKIVAIHT
RHDV-MW460242	EGTPVCDWKKSPISTYGIKKTLSDSTKIDVLAYDGCTQTTHGDCGLPLYDSSGKIVAIHT
RHDV-MN853658	EGTPVCDWKKSPISTYGIKKTLSDSTKIDVLAYDGCTQTTHGDCGLPLYDSSGKIVAIHT
RHDV-MF598301	EGTPVCDWKKSPISTYGIKKTLSDSTKIDVLAYDGCTQTTHGDCGLPLYDSSGKIVAIHT
RHDV-KY679905	EGTPVCDWKKSPISTYGIKKTLSDSTKIDVLAYDGCTQTTHGDCGLPLYDSSGKIVAIHT
RHDV-KY679904	EGTPVCDWKKSPISTYGIKKTLSDSTKIDVLAYDGCTQTTHGDCGLPLYDSSGKIVAIHT
RHDV-KY679903	EGTPVCDWKKSPISTYGIKKTLSDSTKIDVLAYDGCTQTTHGDCGLPLYDSSGKIVAIHT
RHDV-KY319033	EGTPVCDWKKSPISTYGIKKTLSDSTKIDVLAYDGCTQTTHGDCGLPLYDSSGKVVAIHT
RHDV-EU003581	EGTPVCDWKKSPISTYGIKKTLSDSTKIDVLAYDGCTQTTHGDCGLPLYDSSGKVVAIHT
RHDV-MT628289	EGTPVCDWKKSPISTYGIKKTLSDSTKIDVLAYDGCTQTTHGDCGLPLYDSSGKIVAIHT
RHDV2-MW460166	EGTPVCDWKKSPISTYGIKKTLSDSTKINVLAYDGCTQTTHGDCGLPLYDSSGKIVAIHT
RHDV2-MF421565	EGTPVCDWKKSPISTYGIKKTLSDSTKINVLAYDGCTQTTHGDCGLPLYDSSGKIVAIHT
RHDV2-MF421566	EGTPVCDWKKSPISTYGIKKTLSDSTKINVLAYDGCTQTTHGDCGLPLYDSSGKIVAIHT
RHDV2-MF421569	EGTPVCDWKKSPISTYGIKKTLSDSTKINVLAYDGCTQTTHGDCGLPLYDSSGKIVAIHT
RHDV2-MF421570	EGIPVCDWKKSPISTYGIKKTLSDSTKINVLAYDGCTQTTHGDCGLPLYDSSGKIVAIHT
RHDV2-KY628307	EGIPVCDWKKSPISTYGIKKTLSDSTKINVLAYDGCTQTTHGDCGLPLYDSSGKIVAIHT
RHDV2-MF421690	EGTPVCDWKKSPISTYGIKKTLSDSNKIDVLAYDGCTQTTHGDCGLPLYDSSGKIVAIHT
RHDV2-MF421563	EGTPVCDWKKSPISTYGIKKTLSDSTKIDVLAYDGCTQTTHGDCGLPLYDSSGKIVAIHT
RHDV2-MF421564	EGTPVCDWKKSPISTYGIKKTLSDSTKIDVLAYDGCTQTTHGDCGLPLYDSSGKIVAIHT
RHDV2-KT280058	EGTPVCDWKKSPISTYGIKKTLSDSTKIDVLAYDGCTQTTHGDCGLPLYDSSGKIVAIHT

```
RHDV2-KT280059  EGTPVCDWKKSPISTYGIKKTLSDSTKIDVLAYDGCTQTTHGDCGLPLYDSSGKIVAIHT
RHDV2-KY628318  EGTPVCDWKKSPISTYGIKKTLSDSTKIDVLAYDGCTQTTHGDCGLPLYDSSGKIVAIHT
RHDV2-KY628319  EGTPVCDWKKSPISTYGIKKTLSDSTKIDVLAYDGCTQTTHGDCGLPLYDSSGKIVAIHT
RHDV2-MF421578  EGTPVCDWKKSPISTYGIKKTLSDSTKIDVLAYDGCTQTTHGDCGLPLYDSSGKIVAIHT
RHDV2-MF421622  EGTPVCDWKKSPISTYGIKKTLSDSTKIDVLAYDGCTQTTHGDCGLPLYDSSGKIVAIHT
RHDV2-MF421626  EGTPVCDWKKSPISTYGIKKTLSDSTKIDVLAYDGCTQTTHGDCGLPLYDSSGKIVAIHT
RHDV2-MW460167  EGTPVCDWKKSPISTYGIKKTLSDSTKIDVLAYDGCTQTTHGDCGLPLYDSSGKIVAIHT
RHDV2-MF421599  EGTPVYDWKKSPISTYGIKKTLSDSTKIDVLAYDGCTQTTHGDCGLPLYDSSGKIVAIHT
RHDV2-MF421662  EGTPVYDWKKSPISTYGIKKTLSDSTKIDVLAYDGCTQTTHGDCGLPLYDSSGKIVAIHT
RHDV-U54983     EGTPVCDWKKSPISTYGIKKTLSDSTKIDVLAYDGCTQTTHGDCGLPLYDSSGKIVAIHT
RHDV2-MG763941  EGTPVCDWKKSPISTYGMKKTLSDSTKIDVLAYDGTTQTTHGDCGLPLYDECGKIVAIHT
RHDV2-MG763944  EGTPVCDWKKSPISTYGMKKTLSDSTKIDVLAYDGTTQTTHGDCGLPLYDECGKIVAIHT
RHDV2-MG763946  EGTPVCDWKKSPISTYGMKKTLSDSTKIDVLAYDGTTQTTHGDCGLPLYDECGKIVAIHT
RHDV2-MG763952  EGTPVCDWKKSPISTYGMKKTLSDSTKIDVLAYDGTTQTTHGDCGLPLYDECGKIVAIHT
RHDV2-MG763954  EGTPVCDWKKSPISTYGMKKTLSDSTKIDVLAYDGTTQTTHGDCGLPLYDECGKIVAIHT
RHDV2-MW460019  EGTPVCDWKKSPITTYGIKKTLSDSTKIDVLAYDGITQTTHGDCGLPLYDECGKIVAIHT
RHDV2-MW460192  EGTPVCDWKKSPITTYGIKKTLSDSTKIDVLAYDGITQTTHGDCGLPLYDECGKIVAIHT
RHDV2-MF598302  EGTPVCDWKKSPITTYGIKKTLSDSTKIDVLAYDGITQTTHGDCGLPLYDECGKIVAIHT
RHDV-KY628306   EGTPVCDWKKSPITTYGIKKTLSDSTKIDVLAYDGITQTTHGDCGLPLYDECGKIVAIHT
RHDV-KY628308   EGTPVCDWKKSPITTYGIKKTLSDSTKIDVLAYDGITQTTHGDCGLPLYDECGKIVAIHT
RHDV-KY628309   EGTPVCDWKKSPITTYGIKKTLSDSTKIDVLAYDGITQTTHGDCGLPLYDECGKIVAIHT
RHDV-KY628310   EGTPVCDWKKSPITTYGIKKTLSDSTKIDVLAYDGITQTTHGDCGLPLYDECGKIVAIHT
RHDV-KY628311   EGTPVCDWKKSPITTYGIKKTLSDSTKIDVLAYDGITQTTHGDCGLPLYDECGKIVAIHT
RHDV-KY628312   EGTPVCDWKKSPITTYGIKKTLSDSTKIDVLAYDGITQTTHGDCGLPLYDECGKIVAIHT
RHDV-KY628313   EGTPVCDWKKSPITTYGIKKTLSDSTKIDVLAYDGITQTTHGDCGLPLYDECGKIVAIHT
RHDV-KY628314   EGTPVCDWKKSPITTYGIKKTLSDSTKIDVLAYDGITQTTHGDCGLPLYDECGKIVAIHT
RHDV-KY628315   EGTPVCDWKKSPITTYGIKKTLSDSTKIDVLAYDGITQTTHGDCGLPLYDECGKIVAIHT
RHDV-KY628316   EGTPVCDWKKSPITTYGIKKTLSDSTKIDVLAYDGITQTTHGDCGLPLYDECGKIVAIHT
RHDV-KY628317   EGTPVCDWKKSPITTYGIKKTLSDSTKIDVLAYDGITQTTHGDCGLPLYDECGKIVAIHT
RHDV-KY628320   EGTPVCDWKKSPITTYGIKKTLSDSTKIDVLAYDGITQTTHGDCGLPLYDECGKIVAIHT
RHDV-MW460097   EGTPVCDWKKSPITTYGIKKTLSDSTKIDVLAYDGITQTTHGDCGLPLYDECGKIVAIHT
RHDV2-MW460118  EGTPVCDWKKSPITTYGIKKTLSDSTKIDVLAYDGVTQTTHGDCGLPLYDECGKIVAIHT
RHDV2-MW460020  EGTPVCDWKKSPITTYGIKKTLSDSTKIDVLAYDGMTQTTHGDCGLPLYDECGKIVAIHT
RHDV2-MW460228  EGTPVCDWKKSPITTYGIKKTLSDSTKIDVLAYDGTTQTTHGDCGLPLYDECGKIVAIHT
RHDV2-MF421701  EGTPVCDWKKSPITTYGIKKTLSDSTKIDVLAYDGATQTTHGDCGLPLYDECGKIVAIHT
RHDV2-MW460107  EGTPVCDWKKSPISTYGIKKTLSDSTKIDVLAYDGTTQTTHGDCGLPLYDECGKIVAIHT
RHDV2-MW460120  EGTPVCDWKKSPISTYGIKKTLSDSTKIDVLAYDGTTQTTHGDCGLPLYDECGKIVAIHT
RHDV2-MW460122  EGTPVCDWKKSPISTYGIKKTLSDSTKIDVLAYDGTTQTTHGDCGLPLYDECGKIVAIHT
RHDV2-MW460123  EGTPVCDWKKSPISTYGIKKTLSDSTKIDVLAYDGTTQTTHGDCGLPLYDECGKIVAIHT
RHDV2-MW460127  EGTPVCDWKKSPISTYGIKKTLSDSTKIDVLAYDGTTQTTHGDCGLPLYDECGKIVAIHT
RHDV2-MW460128  EGTPVCDWKKSPISTYGIKKTLSDSTKIDVLAYDGTTQTTHGDCGLPLYDECGKIVAIHT
RHDV2-MW460130  EGTPVCDWKKSPISTYGIKKTLSDSTKIDVLAYDGTTQTTHGDCGLPLYDECGKIVAIHT
RHDV2-MW460131  EGTPVCDWKKSPISTYGIKKTLSDSTKIDVLAYDGTTQTTHGDCGLPLYDECGKIVAIHT
RHDV2-MW460135  EGTPVCDWKKSPISTYGIKKTLSDSTKIDVLAYDGTTQTTHGDCGLPLYDECGKIVAIHT
```

```
RHDV2-MW460137  EGTPVCDWKKSPISTYGIKKTLSDSTKIDVLAYDGTTQTTHGDCGLPLYDECGKIVAIHT
RHDV2-MW460140  EGTPVCDWKKSPISTYGIKKTLSDSTKIDVLAYDGTTQTTHGDCGLPLYDECGKIVAIHT
RHDV2-MW460141  EGTPVCDWKKSPISTYGIKKTLSDSTKIDVLAYDGTTQTTHGDCGLPLYDECGKIVAIHT
RHDV2-MW460142  EGTPVCDWKKSPISTYGIKKTLSDSTKIDVLAYDGTTQTTHGDCGLPLYDECGKIVAIHT
RHDV2-MW460144  EGTPVCDWKKSPISTYGIKKTLSDSTKIDVLAYDGTTQTTHGDCGLPLYDECGKIVAIHT
RHDV2-MW460149  EGTPVCDWKKSPISTYGIKKTLSDSTKIDVLAYDGTTQTTHGDCGLPLYDECGKIVAIHT
RHDV2-MW460163  EGTPVCDWKKSPISTYGIKKTLSDSTKIDVLAYDGTTQTTHGDCGLPLYDECGKIVAIHT
RHDV2-MW460169  EGTPVCDWKKSPISTYGIKKTLSDSTKIDVLAYDGTTQTTHGDCGLPLYDECGKIVAIHT
RHDV2-MW460170  EGTPVCDWKKSPISTYGIKKTLSDSTKIDVLAYDGTTQTTHGDCGLPLYDECGKIVAIHT
RHDV2-MW460181  EGTPVCDWKKSPISTYGIKKTLSDSTKIDVLAYDGTTQTTHGDCGLPLYDECGKIVAIHT
RHDV2-MW460230  EGTPVCDWKKSPISTYGIKKTLSDSTKIDVLAYDGTTQTTHGDCGLPLYDECGKIVAIHT
RHDV2-MW460236  EGTPVCDWKKSPISTYGIKKTLSDSTKIDVLAYDGTTQTTHGDCGLPLYDECGKIVAIHT
RHDV2-MW460156  EGTPVCDWKKSPISTYGIKKTLSDSTKIDVLAYDGTTQTTHGDCGLPLYDECGKIVAIHT
RHDV2-MW460110  EGTPVCDWKKSPISTYGIKKTLSDSTKIDVLAYDGTTQTTHGDCGLPLYDECGKIVAIHT
RHDV2-MW460111  EGTPVCDWKKSPISTYGIKKTLSDSTKIDVLAYDGTTQTTHGDCGLPLYDECGKIVAIHT
RHDV2-MW460117  EGTPVCDWKKSPISTYGIKKTLSDSTKIDVLAYDGTTQTTHGDCGLPLYDECGKIVAIHT
RHDV2-MW460119  EGTPVCDWKKSPISTYGIKKTLSDSTKIDVLAYDGTTQTTHGDCGLPLYDECGKIVAIHT
RHDV2-MW460121  EGTPVCDWKKSPISTYGIKKTLSDSTKIDVLAYDGTTQTTHGDCGLPLYDECGKIVAIHT
RHDV2-MW460146  EGTPVCDWKKSPISTYGIKKTLSDSTKIDVLAYDGTTQTTHGDCGLPLYDECGKIVAIHT
RHDV2-MW460151  EGTPVCDWKKSPISTYGIKKTLSDSTKIDVLAYDGTTQTTHGDCGLPLYDECGKIVAIHT
RHDV2-MW460152  EGTPVCDWKKSPISTYGIKKTLSDSTKIDVLAYDGTTQTTHGDCGLPLYDECGKIVAIHT
RHDV2-MW460157  EGTPVCDWKKSPISTYGIKKTLSDSTKIDVLAYDGTTQTTHGDCGLPLYDECGKIVAIHT
RHDV2-MW460158  EGTPVCDWKKSPISTYGIKKTLSDSTKIDVLAYDGTTQTTHGDCGLPLYDECGKIVAIHT
RHDV2-MW460159  EGTPVCDWKKSPISTYGIKKTLSDSTKIDVLAYDGTTQTTHGDCGLPLYDECGKIVAIHT
RHDV2-MW460161  EGTPVCDWKKSPISTYGIKKTLSDSTKIDVLAYDGTTQTTHGDCGLPLYDECGKIVAIHT
RHDV2-MW460165  EGTPVCDWKKSPISTYGIKKTLSDSTKIDVLAYDGTTQTTHGDCGLPLYDECGKIVAIHT
RHDV2-MW460173  EGTPVCDWKKSPISTYGIKKTLSDSTKIDVLAYDGTTQTTHGDCGLPLYDECGKIVAIHT
RHDV2-MW460175  EGTPVCDWKKSPISTYGIKKTLSDSTKIDVLAYDGTTQTTHGDCGLPLYDECGKIVAIHT
RHDV2-MW460177  EGTPVCDWKKSPISTYGIKKTLSDSTKIDVLAYDGTTQTTHGDCGLPLYDECGKIVAIHT
RHDV2-MW460179  EGTPVCDWKKSPISTYGIKKTLSDSTKIDVLAYDGTTQTTHGDCGLPLYDECGKIVAIHT
RHDV2-MW460180  EGTPVCDWKKSPISTYGIKKTLSDSTKIDVLAYDGTTQTTHGDCGLPLYDECGKIVAIHT
RHDV2-MW460183  EGTPVCDWKKSPISTYGIKKTLSDSTKIDVLAYDGTTQTTHGDCGLPLYDECGKIVAIHT
RHDV2-MW460186  EGTPVCDWKKSPISTYGIKKTLSDSTKIDVLAYDGTTQTTHGDCGLPLYDECGKIVAIHT
RHDV2-MW460187  EGTPVCDWKKSPISTYGIKKTLSDSTKIDVLAYDGTTQTTHGDCGLPLYDECGKIVAIHT
RHDV2-MW460190  EGTPVCDWKKSPISTYGIKKTLSDSTKIDVLAYDGTTQTTHGDCGLPLYDECGKIVAIHT
RHDV2-MW460193  EGTPVCDWKKSPISTYGIKKTLSDSTKIDVLAYDGTTQTTHGDCGLPLYDECGKIVAIHT
RHDV2-MW460194  EGTPVCDWKKSPISTYGIKKTLSDSTKIDVLAYDGTTQTTHGDCGLPLYDECGKIVAIHT
RHDV2-MW460195  EGTPVCDWKKSPISTYGIKKTLSDSTKIDVLAYDGTTQTTHGDCGLPLYDECGKIVAIHT
RHDV2-MW460197  EGTPVCDWKKSPISTYGIKKTLSDSTKIDVLAYDGTTQTTHGDCGLPLYDECGKIVAIHT
RHDV2-MW460198  EGTPVCDWKKSPISTYGIKKTLSDSTKIDVLAYDGTTQTTHGDCGLPLYDECGKIVAIHT
RHDV2-MW460202  EGTPVCDWKKSPISTYGIKKTLSDSTKIDVLAYDGTTQTTHGDCGLPLYDECGKIVAIHT
RHDV2-MW460204  EGTPVCDWKKSPISTYGIKKTLSDSTKIDVLAYDGTTQTTHGDCGLPLYDECGKIVAIHT
RHDV2-MW460208  EGTPVCDWKKSPISTYGIKKTLSDSTKIDVLAYDGTTQTTHGDCGLPLYDECGKIVAIHT
RHDV2-MW460210  EGTPVCDWKKSPISTYGIKKTLSDSTKIDVLAYDGTTQTTHGDCGLPLYDECGKIVAIHT
```

```
RHDV2-MW460212  EGTPVCDWKKSPISTYGIKKTLSDSTKIDVLAYDGTTQTTHGDCGLPLYDECGKIVAIHT
RHDV2-MW460213  EGTPVCDWKKSPISTYGIKKTLSDSTKIDVLAYDGTTQTTHGDCGLPLYDECGKIVAIHT
RHDV2-MW460214  EGTPVCDWKKSPISTYGIKKTLSDSTKIDVLAYDGTTQTTHGDCGLPLYDECGKIVAIHT
RHDV2-MW460217  EGTPVCDWKKSPISTYGIKKTLSDSTKIDVLAYDGTTQTTHGDCGLPLYDECGKIVAIHT
RHDV2-MW460220  EGTPVCDWKKSPISTYGIKKTLSDSTKIDVLAYDGTTQTTHGDCGLPLYDECGKIVAIHT
RHDV2-MW460223  EGTPVCDWKKSPISTYGIKKTLSDSTKIDVLAYDGTTQTTHGDCGLPLYDECGKIVAIHT
RHDV2-MW460224  EGTPVCDWKKSPISTYGIKKTLSDSTKIDVLAYDGTTQTTHGDCGLPLYDECGKIVAIHT
RHDV2-MW460226  EGTPVCDWKKSPISTYGIKKTLSDSTKIDVLAYDGTTQTTHGDCGLPLYDECGKIVAIHT
RHDV2-MW460227  EGTPVCDWKKSPISTYGIKKTLSDSTKIDVLAYDGTTQTTHGDCGLPLYDECGKIVAIHT
RHDV2-MW460232  EGTPVCDWKKSPISTYGIKKTLSDSTKIDVLAYDGTTQTTHGDCGLPLYDECGKIVAIHT
RHDV2-MW460233  EGTPVCDWKKSPISTYGIKKTLSDSTKIDVLAYDGTTQTTHGDCGLPLYDECGKIVAIHT
RCV-KX357707    EGTPVCDWKKSPISTYGIKKTLSDSTKIDVLAYDGTTQTTHGDCGLPLYDECGKIVAIHT
RCV-KX357655    EGTPVCDWKKSPISTYGIKKTLSDSTKIDVLAYDGTTQTTHGDCGLPLYDECGKIVAIHT
RCV-KX357696    EGTPVCDWKKSPISTYGIKKTLSDSTKIDVLAYDGTTQTTHGDCGLPLYDECGKIVAIHT
RCV-KX357658    EGTPVCDWKKSPISTYGIKKTLSDSTKIDVLAYDGTTQTTHGDCGLPLYDECGKIVAIHT
RCV-KX357663    EGTPVCDWKKSPISTYGIKKTLSDSTKIDVLAYDGTTQTTHGDCGLPLYDECGKIVAIHT
RCV-KX357703    EGTPVCDWKKSPISTYGIKKTLSDSTKIDVLAYDGTTQTTHGDCGLPLYDECGKIVAIHT
RCV-KX357705    EGTPVCDWKKSPISTYGIKKTLSDSTKIDVLAYDGTTQTTHGDCGLPLYDECGKIVAIHT
RCV-KX357697    EGTPVCDWKKSPISTYGIKKTLSDSTKIDVLAYDGTTQTTHGDCGLPLYDECGKIVAIHT
RCV-KX357700    EGTPVCDWKKSPISTYGIKKTLSDSTKIDVLAYDGTTQTTHGDCGLPLYDECGKIVAIHT
RHDV2-MW460153  EGTPVCDWKKSPISTYGIKKTLSDSTKIDVLAYDGTTQTTHGDCGLPLYDECGKVVAIHT
RHDV2-MW460155  EGTPVCDWKKSPISTYGIKKTLSDSTKIDVLAYDGTTQTTHGDCGLPLYDECGKIVAIHT
RHDV2-MW460178  EGTPVCDWKKSPISTYGIKKTLSDSTKIDVLAYDGTTQTTHGDCGLPLYDECGKIVAIHT
RHDV2-MW460185  EGTPVCDWKKSPISTYGIKKTLSDSTKIDVLAYDGTTQTTHGDCGLPLYDECGKIVAIHT
RHDV2-MW460211  EGTPVCDWKKSPISTYGIKKTLSDSTKIDVLAYDGTTQTTHGDCGLPLYDECGKIVAIHT
RCV-KX357689    EGTPVCDWKKSPISTYGIKKTLSDSTKIDVLAYDGTTQTTHGDCGLPLYDECGKIVAIHT
RCV-KX357682    EGTPVCDWKKSPISTYGIKKTLSDSTKIDVLAYDGTTQTTHGDCGLPLYDECGKIVAIHT
RCV-KX357691    EGTPVCDWKKSPISTYGIKKTLSDSTKIDVLAYDGTTQTTHGDCGLPLYDECGKIVAIHT
RCV-KX357683    EGTPVCDWKKSPISTYGIKKTLSDSTKIDVLAYDGTTQTTHGDCGLPLYDECGKIVAIHT
RCV-KX357686    EGTPVCDWKKSPISTYGIKKTLSDSTKIDVLAYDGTTQTTHGDCGLPLYDECGKIVAIHT
RCV-KX357685    EGTPVCDWKKSPISTYGIKKTLSDSTKIDVLAYDGTTQTTHGDCGLPLYDECGKIVAIHT
RCV-KX357692    EGTPVCDWKKSPISTYGIKKTLSDSTKIDVLAYDGTTQTTHGDCGLPLYDECGKIVAIHT
RCV-KX357684    EGTPVCDWKKSPISTYGIKKTLSDSTKIDVLAYDGTTQTTHGDCGLPLYDECGKIVAIHT
RCV-KX357690    EGTPVCDWKKSPISTYGIKKTLSDSTKIDVLAYDGTTQTTHGDCGLPLYDECGKIVAIHT
RCV-KX357687    EGTPVCDWKKSPISTYGIKKTLSDSTKIDVLAYDGTTQTTHGDCGLPLYDECGKIVAIHT
RCV-KX357701    EGTPVCDWKKSPISTYGIKKTLSDSTKIDVLAYDGTTQTTHGDCGLPLYDECGKIVAIHT
RHDV2-MW460219  EGTPVCDWKKSPISTYGIKKTLSDSTKIDVLAYDGTTQTTHGDCGLPLYDECGKIVAIHT
RCV-KX357693    EGTPVCDWKKSPISTYGIKKTLSDSTKIDVLAYDGTTQTTHGDCGLPLYDECGKIVAIHT
RCV-EU871528    EGTPVCDWKKSPISTYGIKKTLSDSTKIDVLAYDGTTQTTHGDCGLPLYDECGKIVAIHT
RCV-KX357673    EGTPVCDWKKSPISTYGIKKTLSDSTKIDVLAYDGTTQTTHGDCGLPLYDECGKIVAIHT
RCV-KX357675    EGTPVCDWKKSPISTYGIKKTLSDSTKIDVLAYDGTTQTTHGDCGLPLYDECGKIVAIHT
RCV-KX357694    EGTPVCDWKKSPISTYGIKKTLSDSTKIDVLAYDGTTQTTHGDCGLPLYDECGKIVAIHT
RHDV2-MW460199  EGTPVCDWKKSPISTYGIKKTLSDSTKIDVLAYDGTTQTTHGDCGLPLYDECGKIVAIHT
RHDV2-MW460200  EGTPVCDWKKSPISTYGIKKTLSDSTKIDVLAYDGTTQTTHGDCGLPLYDECGKIVAIHT
```

```
RCV-KX357665     EGTPVCDWKKSPISTYGIKKTLSDSTKIDVLAYDGTTQTTHGDCGLPLYDECGKIVAIHT
RCV-KX357681     EGTPVCDWKKSPISTYGIKKTLSDSTKIDVLAYDGTTQTTHGDCGLPLYDECGKIVAIHT
RCV-KX357662     EGTPVCDWKKSPISTYGIKKTLSDSTKIDVLAYDGTTQTTHGDCGLPLYDECGKIVAIHT
RCV-KX357661     EGTPVCDWKKSPISTYGIKKTLSDSTKIDVLAYDGTTQTTHGDCGLPLYDECGKIVAIHT
RCV-KX357680     EGTPVCDWKKSPISTYGIKKTLSDSTKIDVLAYDGTTQTTHGDCGLPLYDECGKIVAIHT
RCV-KX357679     EGTPVCDWKKSPISTYGIKKTLSDSTKIDVLAYDGTTQTTHGDCGLPLYDECGKIVAIHT
RCV-KX357664     EGTPVCDWKKSPISTYGIKKTLSDSTKIDVLAYDGTTQTTHGDCGLPLYDECGKIVAIHT
RCV-KX357674     EGTPVCDWKKSPISTYGIKKTLSDSTKIDVLAYDGTTQTTHGDCGLPLYDECGKIVAIHT
RCV-KX357669     EGTPVCDWKKSPISTYGIKKTLSDSTKIDVLAYDGTTQTTHGDCGLPLYDECGKIVAIHT
RCV-KX357666     EGTPVCDWKKSPISTYGIKKTLSDSTKIDVLAYDGTTQTTHGDCGLPLYDECGKIVAIHT
RCV-KX357659     EGTPVCDWKKSPISTYGIKKTLSDSTKIDVLAYDGTTQTTHGDCGLPLYDECGKIVAIHT
RCV-KX357676     EGTPVCDWKKSPISTYGIKKTLSDSTKIDVLAYDGTTQTTHGDCGLPLYDECGKIVAIHT
RCV-KX357660     EGTPVCDWKKSPISTYGIKKTLSDSTKIDVLAYDGTTQTTHGDCGLPLYDECGKIVAIHT
RCV-KX357656     EGTPVCDWKKSPISTYGIKKTLSDSTKIDVLAYDGITQTTHGDCGLPLYDECGKIVAIHT
RHDV2-MW460150   EGTPVCDWKKSPISTYGIKKTLSDSTKIDVLAYDGTTQTTHGDCGLPLYDECGKIVAIHT
HaCV-MK138383    EGVPVTDWKRATISTYGLKKTFSDSTKIDVLAYDGPTQTTHGDCGLPLFDETGKIVAIHT
HaCV-MH204883    EGTPVRDWKKAAITTYGLKKTFSDSTKIDVLAYDGPTQTTHGDCGLPLFDETGKVVAIHT
EBHSV-MK440616   EGTPVRDWKKASITTYGLKKTFSDSTKIDVLAYDGPTQTTHGDCGLPLFDEAGKVVAIHT
EBHSV-MK440613   EGTPVRDWKKASITTYGLKKTFSDSTKIDVLAYDGPTQTTHGDCGLPLFDEAGKVVAIHT
EBHSV-LR899171   EGTPVRDWKKASITTYGLKKTFSDSTKIDVLAYDGPTQTTHGDCGLPLFDEAGKVVAIHT
EBHSV-LR899152   EGTPVRDWKKASITTYGLKKTFSDSTKIDVLAYDGPTQTTHGDCGLPLFDEAGKVVAIHT
EBHSV-LR899140   EGTPVRDWKKASITTYGLKKTFSDSTKIDVLAYDGPTQTTHGDCGLPLFDEAGKVVAIHT
EBHSV-LR899188   EGTPVRDWKKASITTYGLKKTFSDSTKIDVLAYDGPTQTTHGDCGLPLFDEAGKVVAIHT
EBHSV-LR899185   EGTPVRDWKKASITTYGLKKTFSDSTKIDVLAYDGPTQTTHGDCGLPLFDEAGKVVAIHT
EBHSV-LR899182   EGTPVRDWKKASITTYGLKKTFSDSTKIDVLAYDGPTQTTHGDCGLPLFDEAGKVVAIHT
EBHSV-MK440614   EGTPVRDWKKASITTYGLKKTFSDSTKIDVLAYDGPTQTTHGDCGLPLFDEAGKVVAIHT
EBHSV-MK440615   EGTPVRDWKKASITTYGLKKTFSDSTKIDVLAYDGPTQTTHGDCGLPLFDEAGKVVAIHT
EBHSV-KC832838   EGTPVRDWKKASITTYGLKKTFSDSTKIDVLAYDGPTQTTHGDCGLPLFDEAGKVVAIHT
EBHSV-KC832839   EGTPVRDWKKASITTYGLKKTFSDSTKIDVLAYDGPTQTTHGDCGLPLXDEAGKVVAIHT
EBHSV-MK440617   EGTPVRDWKRASITTYGLKKTFSDSTKIDVLAYDGPTQTTHGDCGLPLFDEAGKVVAIHT
EBHSV-Z69620     EGTPVRDWKRASITTYGLKKTFSDSTKIDVLAYDGPTQTTHGDCGLPLFDEAGKVVAIHT
HaCV-KR230102    EGTPVRDWKKAAITTYGIKKTFSDSTKIDVLAYDGPTQTTHGDCGLPLFDESGKVVAIHT
HaCV-MK138384    EGNPVSDWKKAHVSTYGMKKTFSDSTKIDVLAYDGPTQTTHGDCGLPLYDETGKIVAIHT
HaCV-MK138385    EGTPVVDWKKAHVSTYGMKKTFSDSTKIDVLAYDGSTQTTHGDCGLPLFDETGKVVAIHT
                 ** ** **::: ::***:*:*:***.:::**:*** ******.***** *  **:*****

RHDV2-MN901451   GKLLGFSKMCTLIDLTVTKGVYE
RHDV2-MN061492   GKLLGFSKMCTLIDLTVTKGVYE
RHDV2-MN738377   GKLLGFSKMCTLIDLTVTKGVYE
RHDV2-MN786321   GKLLGFSKMCTLIDLTVTKGVYE
RHDV2-MT506233   GKLLGFSKMCTLIDLTVTKGVYE
RHDV2-MT506234   GKLLGFSKMCTLIDLTVTKGVYE
RHDV2-MT506235   GKLLGFSKMCTLIDLTVTKGVYE
RHDV2-MT506236   GKLLGFSKMCTLIDLTVTKGVYE
```

```
RHDV2-MW926372  GKLLGFSKMCTLIDLTVTKGVYE
RHDV2-MW926373  GKLLGFSKMCTLIDLTVTKGVYE
RHDV2-MW926374  GKLLGFSKMCTLIDLTVTKGVYE
RHDV2-MW926375  GKLLGFSKMCTLIDLTVTKGVYE
RHDV2-MW926376  GKLLGFSKMCTLIDLTVTKGVYE
RHDV2-MW926377  GKLLGFSKMCTLIDLTVTKGVYE
RHDV2-MW926379  GKLLGFSKMCTLIDLTVTKGVYE
RHDV2-MW926380  GKLLGFSKMCTLIDLTVTKGVYE
RHDV2-MW926382  GKLLGFSKMCTLIDLTVTKGVYE
RHDV2-MW926383  GKLLGFSKMCTLIDLTVTKGVYE
RHDV2-MW926384  GKLLGFSKMCTLIDLTVTKGVYE
RHDV2-MW926371  GKLLGFSKMCTLIDLTVTKGVYE
RHDV2-MN853660  GKLLGFSKMCTLIDLTVTKGVYE
RHDV2-MN853661  GKLLGFSKMCTLIDLTVTKGVYE
RHDV2-MW926381  GKLLGFSKMCTLIDLTVTKGVYE
RHDV2-MN853659  GKLLGFSKMCTLIDLTVTKGVYE
RHDV2-MW926378  GKLLGFSKMCTLIDLTVTRGVYE
RHDV2-MG763942  GKLLGFSKMCTLIDLTVTKGVYE
RHDV2-MG763943  GKLLGFSKMCTLIDLTVTKGVYE
RHDV2-MG763945  GKLLGFSKMCTLIDLTVTKGVYE
RHDV2-MG763949  GKLLGFSKMCTLIDLTVTKGVYE
RHDV2-MG763950  GKLLGFSKMCTLIDLTVTKGVYE
RHDV2-MG763951  GKLLGFSKMCTLIDLTVTKGVYE
RHDV2-MF407653  GKLLGFSKMCTLIDLTVTKGVYE
RHDV2-MN737113  GKLLGFSKMCTLIDLTVTKGVYE
RHDV-MT628288   GKLLGFSKMCTLIDLTVTKGVYE
RHDV-MT628291   GKLLGFSKMCTLIDLTVTKGVYE
RCV-MN746289    GKLLGFSKMCTLIDLTVTKGVYE
RCV-MN746288    GKLLGFSKMCTLIDLTVTKGVYE
RCV-MN737117    GKLLGFSKMCTLIDLTVTKGVYE
RHDV2-MN737114  GKLLGFSKMCTLIDLTVTKGVYE
RHDV2-MW974834  GKLLGFSKMCTLIDLTVTKGVYE
RHDV2-MT586027  GKLLGFSKMCTLIDLTVTKGVYE
RHDV-MT628290   GKLLGFSKMCTLIDLTVTKGVYE
RCV-MN737115    GKLLGFSKMCTLIDLTVTKGVYE
RCV-MN737116    GKLLGFSKMCTLIDLTVTKGVYE
RHDV2-MK521927  GKLLGFSKMCTLIDLTITKGVYE
RHDV2-MG602006  GKLLGFSKMCTLIDLTITKGVYE
RHDV2-MG602007  GKLLGFSKMCTLIDLTITKGVYE
RHDV2-MG763937  GKLLGFSKMCTLIDLTITKGVYE
RHDV2-MG763938  GKLLGFSKMCTLIDLTITKGVYE
RHDV2-MG763939  GKLLGFSKMCTLIDLTITKGVYE
RHDV2-MG763940  GKLLGFSKMCTLIDLTITKGVYE
RHDV2-MG763948  GKLLGFSKMCTLIDLTITKGVYE
```

```
RHDV2-MG763953  GKLLGFSKMCTLIDLTITKGVYE
RHDV2-MF407654  GKLLGFSKMCTLIDLTITKGVYE
RHDV2-MF407656  GKLLGFSKMCTLIDLTITKGVYE
RHDV2-MW460036  GKLLGFSKMCTLIDLTITKGVYE
RHDV2-MW460101  GKLLGFSKMCTLIDLTITKGVYE
RHDV2-MW460102  GKLLGFSKMCTLIDLTITKGVYE
RHDV2-MW460103  GKLLGFSKMCTLIDLTITKGVYE
RHDV2-MW460105  GKLLGFSKMCTLIDLTITKGVYE
RHDV2-MW460106  GKLLGFSKMCTLIDLTITKGVYE
RHDV2-MW460112  GKLLGFSKMCTLIDLTITKGVYE
RHDV2-MW460113  GKLLGFSKMCTLIDLTITKGVYE
RHDV2-MW460114  GKLLGFSKMCTLIDLTITKGVYE
RHDV2-MW460115  GKLLGFSKMCTLIDLTITKGVYE
RHDV2-MW460116  GKLLGFSKMCTLIDLTITKGVYE
RHDV2-MW460126  GKLLGFSKMCTLIDLTITKGVYE
RHDV2-MW460138  GKLLGFSKMCTLIDLTITKGVYE
RHDV2-MW460145  GKLLGFSKMCTLIDLTITKGVYE
RHDV2-MW460162  GKLLGFSKMCTLIDLTITKGVYE
RHDV2-MW460172  GKLLGFSKMCTLIDLTITKGVYE
RHDV2-MW460176  GKLLGFSKMCTLIDLTITKGVYE
RHDV2-MW460182  GKLLGFSKMCTLIDLTITKGVYE
RHDV2-MW460188  GKLLGFSKMCTLIDLTITKGVYE
RHDV2-MW460189  GKLLGFSKMCTLIDLTITKGVYE
RHDV2-MW460191  GKLLGFSKMCTLIDLTITKGVYE
RHDV2-MW460203  GKLLGFSKMCTLIDLTITKGVYE
RHDV2-MW460205  GKLLGFSKMCTLIDLTITKGVYE
RHDV2-MW460206  GKLLGFSKMCTLIDLTITKGVYE
RHDV2-MW460207  GKLLGFSKMCTLIDLTITKGVYE
RHDV2-MW460215  GKLLGFSKMCTLIDLTITKGVYE
RHDV2-MW460221  GKLLGFSKMCTLIDLTITKGVYE
RHDV2-MW460222  GKLLGFSKMCTLIDLTITKGVYE
RHDV2-MW460225  GKLLGFSKMCTLIDLTITKGVYE
RHDV2-MW467791  GKLLGFSKMCTLIDLTITKGVYE
RHDV2-MF421574  GKLLGFSKMCTLIDLTITKGVYE
RHDV2-MF421576  GKLLGFSKMCTLIDLTITKGVYE
RHDV2-MF421577  GKLLGFSKMCTLIDLTITKGVYE
RHDV2-MF421579  GKLLGFSKMCTLIDLTITKGVYE
RHDV2-MF421583  GKLLGFSKMCTLIDLTITKGVYE
RHDV2-MF421584  GKLLGFSKMCTLIDLTITKGVYE
RHDV2-MF421585  GKLLGFSKMCTLIDLTITKGVYE
RHDV2-MF421586  GKLLGFSKMCTLIDLTITKGVYE
RHDV2-MF421587  GKLLGFSKMCTLIDLTITKGVYE
RHDV2-MF421589  GKLLGFSKMCTLIDLTITKGVYE
RHDV2-MF421590  GKLLGFSKMCTLIDLTITKGVYE
```

```
RHDV2-MF421591  GKLLGFSKMCTLIDLTITKGVYE
RHDV2-MF421592  GKLLGFSKMCTLIDLTITKGVYE
RHDV2-MF421593  GKLLGFSKMCTLIDLTITKGVYE
RHDV2-MF421594  GKLLGFSKMCTLIDLTITKGVYE
RHDV2-MF421595  GKLLGFSKMCTLIDLTITKGVYE
RHDV2-MF421596  GKLLGFSKMCTLIDLTITKGVYE
RHDV2-MF421597  GKLLGFSKMCTLIDLTITKGVYE
RHDV2-MF421598  GKLLGFSKMCTLIDLTITKGVYE
RHDV2-MF421600  GKLLGFSKMCTLIDLTITKGVYE
RHDV2-MF421601  GKLLGFSKMCTLIDLTITKGVYE
RHDV2-MF421602  GKLLGFSKMCTLIDLTITKGVYE
RHDV2-MF421603  GKLLGFSKMCTLIDLTITKGVYE
RHDV2-MF421604  GKLLGFSKMCTLIDLTITKGVYE
RHDV2-MF421606  GKLLGFSKMCTLIDLTITKGVYE
RHDV2-MF421607  GKLLGFSKMCTLIDLTITKGVYE
RHDV2-MF421608  GKLLGFSKMCTLIDLTITKGVYE
RHDV2-MF421609  GKLLGFSKMCTLIDLTITKGVYE
RHDV2-MF421610  GKLLGFSKMCTLIDLTITKGVYE
RHDV2-MF421611  GKLLGFSKMCTLIDLTITKGVYE
RHDV2-MF421612  GKLLGFSKMCTLIDLTITKGVYE
RHDV2-MF421613  GKLLGFSKMCTLIDLTITKGVYE
RHDV2-MF421615  GKLLGFSKMCTLIDLTITKGVYE
RHDV2-MF421617  GKLLGFSKMCTLIDLTITKGVYE
RHDV2-MF421618  GKLLGFSKMCTLIDLTITKGVYE
RHDV2-MF421620  GKLLGFSKMCTLIDLTITKGVYE
RHDV2-MF421624  GKLLGFSKMCTLIDLTITKGVYE
RHDV2-MF421625  GKLLGFSKMCTLIDLTITKGVYE
RHDV2-MF421627  GKLLGFSKMCTLIDLTITKGVYE
RHDV2-MF421636  GKLLGFSKMCTLIDLTITKGVYE
RHDV2-MF421637  GKLLGFSKMCTLIDLTITKGVYE
RHDV2-MF421638  GKLLGFSKMCTLIDLTITKGVYE
RHDV2-MF421639  GKLLGFSKMCTLIDLTITKGVYE
RHDV2-MF421642  GKLLGFSKMCTLIDLTITKGVYE
RHDV2-MF421643  GKLLGFSKMCTLIDLTITKGVYE
RHDV2-MF421645  GKLLGFSKMCTLIDLTITKGVYE
RHDV2-MF421646  GKLLGFSKMCTLIDLTITKGVYE
RHDV2-MF421647  GKLLGFSKMCTLIDLTITKGVYE
RHDV2-MF421648  GKLLGFSKMCTLIDLTITKGVYE
RHDV2-MF421649  GKLLGFSKMCTLIDLTITKGVYE
RHDV2-MF421650  GKLLGFSKMCTLIDLTITKGVYE
RHDV2-MF421651  GKLLGFSKMCTLIDLTITKGVYE
RHDV2-MF421652  GKLLGFSKMCTLIDLTITKGVYE
RHDV2-MF421654  GKLLGFSKMCTLIDLTITKGVYE
RHDV2-MF421655  GKLLGFSKMCTLIDLTITKGVYE
```

```
RHDV2-MF421656  GKLLGFSKMCTLIDLTITKGVYE
RHDV2-MF421658  GKLLGFSKMCTLIDLTITKGVYE
RHDV2-MF421659  GKLLGFSKMCTLIDLTITKGVYE
RHDV2-MF421660  GKLLGFSKMCTLIDLTITKGVYE
RHDV2-MF421661  GKLLGFSKMCTLIDLTITKGVYE
RHDV2-MF421663  GKLLGFSKMCTLIDLTITKGVYE
RHDV2-MF421664  GKLLGFSKMCTLIDLTITKGVYE
RHDV2-MF421665  GKLLGFSKMCTLIDLTITKGVYE
RHDV2-MF421666  GKLLGFSKMCTLIDLTITKGVYE
RHDV2-MF421667  GKLLGFSKMCTLIDLTITKGVYE
RHDV2-MF421668  GKLLGFSKMCTLIDLTITKGVYE
RHDV2-MF421669  GKLLGFSKMCTLIDLTITKGVYE
RHDV2-MF421670  GKLLGFSKMCTLIDLTITKGVYE
RHDV2-MF421673  GKLLGFSKMCTLIDLTITKGVYE
RHDV2-MF421674  GKLLGFSKMCTLIDLTITKGVYE
RHDV2-MF421675  GKLLGFSKMCTLIDLTITKGVYE
RHDV2-MF421676  GKLLGFSKMCTLIDLTITKGVYE
RHDV2-MF421678  GKLLGFSKMCTLIDLTITKGVYE
RHDV2-MF421680  GKLLGFSKMCTLIDLTITKGVYE
RHDV2-MF421681  GKLLGFSKMCTLIDLTITKGVYE
RHDV2-MF421682  GKLLGFSKMCTLIDLTITKGVYE
RHDV2-MF421683  GKLLGFSKMCTLIDLTITKGVYE
RHDV2-MF421684  GKLLGFSKMCTLIDLTITKGVYE
RHDV2-MF421685  GKLLGFSKMCTLIDLTITKGVYE
RHDV2-MF421689  GKLLGFSKMCTLIDLTITKGVYE
RHDV2-MF421691  GKLLGFSKMCTLIDLTITKGVYE
RHDV2-MF421694  GKLLGFSKMCTLIDLTITKGVYE
RHDV2-MF421695  GKLLGFSKMCTLIDLTITKGVYE
RHDV2-MF421696  GKLLGFSKMCTLIDLTITKGVYE
RHDV2-MG763936  GKLLGFSKMCTLIDLTITKGVYE
RHDV2-MG763947  GKLLGFSKMCTLIDLTITKGVYE
RHDV2-MW460124  GKLLGFSKMCTLIDLTITKGVYE
RHDV2-MW460125  GKLLGFSKMCTLIDLTITKGVYE
RHDV2-MW460129  GKLLGFSKMCTLIDLTITKGVYE
RHDV2-MW460147  GKLLGFSKMCTLIDLTITKGVYE
RHDV2-MW460148  GKLLGFSKMCTLIDLTITKGVYE
RHDV2-MW460231  GKLLGFSKMCTLIDLTITKGVYE
RHDV2-MW460234  GKLLGFSKMCTLIDLTITKGVYE
RHDV2-MW460237  GKLLGFSKMCTLIDLTITKGVYE
RHDV2-MW460240  GKLLGFSKMCTLIDLTITKGVYE
RHDV2-MF421628  GKLLGFSKMCTLIDLTITKGVYE
RHDV2-MF421629  GKLLGFSKMCTLIDLTITKGVYE
RHDV2-MF421630  GKLLGFSKMCTLIDLTITKGVYE
RHDV2-MF421631  GKLLGFSKMCTLIDLTITKGVYE
```

```
RHDV2-MF421633  GKLLGFSKMCTLIDLTITKGVYE
RHDV2-MF421634  GKLLGFSKMCTLIDLTITKGVYE
RHDV2-MF421635  GKLLGFSKMCTLIDLTITKGVYE
RHDV2-MF421697  GKLLGFSKMCTLIDLTITKGVYE
RHDV2-MF421698  GKLLGFSKMCTLIDLTITKGVYE
RHDV2-MF421699  GKLLGFSKMCTLIDLTITKGVYE
RHDV2-MF421605  GKLLGFSKMCTLIDLTITKGVYE
RHDV2-MN737112  GKLLGFSKMCTLVDLTITKGVYE
RHDV2-MF421614  GKLLGFSKMCTLVDLTITKGVYE
RHDV2-MF421657  GKLLGFSKMCTLVDLTITKGVYE
RHDV2-MF421671  GKLLGFSKMCTLVDLTITKGVYE
RHDV2-MW460108  GKLLGFSKMCTLIDLTITKGVYE
RHDV2-MW460109  GKLLGFSKMCTLIDLTITKGVYE
RHDV2-MW460201  GKLLGFSKMCTLIDLTITKGVYE
RHDV2-MF421575  GKLLGFSKMCTLIDLTITKGVYE
RHDV2-MF421580  GKLLGFSKMCTLIDLTITKGVYE
RHDV2-MF421581  GKLLGFSKMCTLIDLTITKGVYE
RHDV2-MF421582  GKLLGFSKMCTLIDLTITKGVYE
RHDV2-MF421672  GKLLGFSKMCTLIDLTITKGVYE
RHDV2-MW460164  GKLLGFSKMCTLIDLTVTKGVYE
RHDV2-MW460174  GKLLGFSKMCTLIDLTVTKGVYE
RHDV2-MF421619  GKLLGFSKMCTLIDLTVTKGVYE
RHDV2-MW460168  GKLLGFSKMCTLIDLTITKGVYE
RHDV2-MW460171  GKLLGFSKMCTLIDLTITKGVYE
RHDV2-MW460229  GKLLGFSKMCTLIDLTITKGVYE
RHDV2-MF421616  GKLLGFSKMCTLIDLTITKGVYE
RHDV2-MF421621  GKLLGFSKMCTLIDLTITKGVYE
RHDV2-MF421623  GKLLGFSKMCTLIDLTITKGVYE
RHDV2-MF421686  GKLLGFSKMCTLIDLTITKGVYE
RHDV2-MF421687  GKLLGFSKMCTLIDLTITKGVYE
RHDV2-MF421688  GKLLGFSKMCTLIDLTITKGVYE
RHDV2-MF421588  GKLLGFSKMCTLIDLTITKGVYE
RHDV2-MF421692  GKLLGFSKMCTLIDLTITKGVYE
RHDV2-MF421640  GKLLGFSKMCTLIDLTITKGVYE
RHDV2-MF421641  GKLLGFSKMCTLIDLTITKGVYE
RHDV2-MF421653  GKLLGFSKMCTLIDLTITKGVYE
RHDV2-MF421677  GKLLGFSKMCTLIDLTITKGVYE
RHDV2-MF421679  GKLLGFSKMCTLIDLTITKGVYE
RHDV2-MF421693  GKLLGFSKMCTLIDLTITKGVYE
RHDV2-MF421644  GKLLGFSKMCTLIDLTITKGVYE
RHDV2-MF421632  GKLLGFSKMCTLIDLTVTKGVYE
RHDV2-MF421700  GKLLGFSKMCTLIDLTITKGVYE
RHDV2-MW460132  GKLLGFSKMCTLIDLTITKGVYE
RHDV2-MW460133  GKLLGFSKMCTLIDLTITKGVYE
```

```
RHDV2-MW460134  GKLLGFSKMCTLIDLTITKGVYE
RHDV2-MW460143  GKLLGFSKMCTLIDLTITKGVYE
RHDV2-MF407651  GKLLGFSKMCTLIDLTITKGVYE
RHDV2-MF407652  GKLLGFSKMCTLIDLTITKGVYE
RHDV2-MF421567  GKLLGFSKMCTLIDLTITKGVYE
RHDV2-MF421568  GKLLGFSKMCTLIDLTITKGVYE
RHDV2-MF421571  GKLLGFSKMCTLIDLTITKGVYE
RHDV2-MF421572  GKLLGFSKMCTLIDLTITKGVYE
RHDV2-MF421573  GKLLGFSKMCTLIDLTITKGVYE
RHDV-MH190418   GKLLGFSKMCTLIDLTITKGVYE
RHDV-EU003579   GKLLGFSKMCTLIDLTITKGVYE
RHDV-EU003580   GKLLGFSKMCTLIDLTITKGVYE
RHDV-KP144789   GKLLGFSKMCTLIDLTITKGVYE
RHDV-KU882093   GKLLGFSKMCTLIDLTITKGVYE
RHDV-KU882092   GKLLGFSKMCTLIDLTITKGVYE
RHDV-X87607     GKLLGFSKMCTLIDLTITKGVYE
RHDV-Z29514     GKLLGFSKMCTLIDLTITKGVYE
RHDV-M67473     GKLLGFSKMCTLIDLTITKGVYE
RHDV-MG602005   GKLLGFSKMCTLIDLTITKGVYE
RHDV-KY679902   GKLLGFSKMCTLVDLTITKGVYE
RHDV-KY319031   GKLLGFSKMCTLVDLTITKGVYE
RHDV-KY319032   GKLLGFSKMCTLVDLTITKGVYE
RHDV-KY319034   GKLLGFSKMCTLVDLTITKGVYE
RHDV-KY319035   GKLLGFSKMCTLVDLTITKGVYE
RHDV-KF677011   GKLLGFSKMCTLVDLTITKGVYE
RHDV-EU003578   GKLLGFSKMCTLVDLTITKGVYE
RHDV-EU003582   GKLLGFSKMCTLVDLTITKGVYE
RHDV-MW460041   GKLLGFSKMCTLVDLTITKGVYE
RHDV-MW460063   GKLLGFSKMCTLVDLTITKGVYE
RHDV-MW460075   GKLLGFSKMCTLVDLTITKGVYE
RHDV-MW460094   GKLLGFSKMCTLVDLTITKGVYE
RHDV-MW460104   GKLLGFSKMCTLVDLTITKGVYE
RHDV-MW460139   GKLLGFSKMCTLVDLTITKGVYE
RHDV-MW460154   GKLLGFSKMCTLVDLTITKGVYE
RHDV-MW460184   GKLLGFSKMCTLVDLTITKGVYE
RHDV-MW460196   GKLLGFSKMCTLVDLTITKGVYE
RHDV-MW460209   GKLLGFSKMCTLVDLTITKGVYE
RHDV-MW460216   GKLLGFSKMCTLVDLTITKGVYE
RHDV-MW460235   GKLLGFSKMCTLVDLTITKGVYE
RHDV-MW460239   GKLLGFSKMCTLVDLTITKGVYE
RHDV-MW460241   GKLLGFSKMCTLVDLTITKGVYE
RHDV-MW460242   GKLLGFSKMCTLVDLTITKGVYE
RHDV-MN853658   GKLLGFSKMCTLVDLTITKGVYE
RHDV-MF598301   GKLLGFSKMCTLVDLTITKGVYE
```

```
RHDV-KY679905   GKLLGFSKMCTLVDLTITKGVYE
RHDV-KY679904   GKLLGFSKMCTLVDLTITKGVYE
RHDV-KY679903   GKLLGFSKMCTLVDLTITKGVYE
RHDV-KY319033   GKLLGFSKMCTLVDLTITKGVYE
RHDV-EU003581   GKLLGFSKMCTLVDLTITKGVYE
RHDV-MT628289   GKLLGFSKMCTLIDLTVTKGVYE
RHDV2-MW460166  GKLLGFSKMCTLIDLTITKGVYE
RHDV2-MF421565  GKLLGFSKMCTLIDLTITKGVYE
RHDV2-MF421566  GKLLGFSKMCTLIDLTITKGVYE
RHDV2-MF421569  GKLLGFSKMCTLIDLTITKGVYE
RHDV2-MF421570  GKLLGFSKMCTLIDLTITKGVYE
RHDV2-KY628307  GKLLGFSKMCTLIDLTITKGVYE
RHDV2-MF421690  GKLLGFSKMCTLIDLTITKGVYE
RHDV2-MF421563  GKLLGFSKMCTLIDLTVTKGVYE
RHDV2-MF421564  GKLLGFSKMCTLIDLTVTKGVYE
RHDV2-KT280058  GKLLGFSKMCTLIDLTVTKGVYE
RHDV2-KT280059  GKLLGFSKMCTLIDLTVTKGVYE
RHDV2-KY628318  GKLLGFSKMCTLIDLTVTKGVYE
RHDV2-KY628319  GKLLGFSKMCTLIDLTVTKGVYE
RHDV2-MF421578  GKLLGFSKMCTLIDLTITKGVYE
RHDV2-MF421622  GKLLGFSKMCTLIDLTITKGVYE
RHDV2-MF421626  GKLLGFSKMCTLIDLTITKGVYE
RHDV2-MW460167  GKLLGFSKMCTLIDLTITKGVYE
RHDV2-MF421599  GKLLGFSKMCTLIDLTITKGVYE
RHDV2-MF421662  GKLLGFSKMCTLIDLTITKGVYE
RHDV-U54983     GKLLGFSKMCTLIDLTITKGVYE
RHDV2-MG763941  GKLLGFSKMCTLIDLTVTKGVYE
RHDV2-MG763944  GKLLGFSKMCTLIDLTVTKGVYE
RHDV2-MG763946  GKLLGFSKMCTLIDLTVTKGVYE
RHDV2-MG763952  GKLLGFSKMCTLIDLTVTKGVYE
RHDV2-MG763954  GKLLGFSKMCTLIDLTVTKGVYE
RHDV2-MW460019  GKLLGFSKMCTLIDLTVTKGVYE
RHDV2-MW460192  GKLLGFSKMCTLIDLTVTKGVYE
RHDV2-MF598302  GKLLGFSKMCTLIDLTVTKGVYE
RHDV-KY628306   GKLLGFSKMCTLIDLTVTKGVYE
RHDV-KY628308   GKLLGFSKMCTLIDLTVTKGVYE
RHDV-KY628309   GKLLGFSKMCTLIDLTVTKGVYE
RHDV-KY628310   GKLLGFSKMCTLIDLTVTKGVYE
RHDV-KY628311   GKLLGFSKMCTLIDLTVTKGVYE
RHDV-KY628312   GKLLGFSKMCTLIDLTVTKGVYE
RHDV-KY628313   GKLLGFSKMCTLIDLTVTKGVYE
RHDV-KY628314   GKLLGFSKMCTLIDLTVTKGVYE
RHDV-KY628315   GKLLGFSKMCTLIDLTVTKGVYE
RHDV-KY628316   GKLLGFSKMCTLIDLTVTKGVYE
```

```
RHDV-KY628317   GKLLGFSKMCTLIDLTVTKGVYE
RHDV-KY628320   GKLLGFSKMCTLIDLTVTKGVYE
RHDV-MW460097   GKLLGFSKMCTLIDLTVTKGVYE
RHDV2-MW460118  GKLLGFSKMCTLIDLTVTKGVYE
RHDV2-MW460020  GKLLGFSKMCTLIDLTVTKGVYE
RHDV2-MW460228  GKLLGFSKMCTLIDLTVTKGVYE
RHDV2-MF421701  GKLLGFSKMCTLIDLTVTKGVYE
RHDV2-MW460107  GKLLGFSKMCTLIDLTVTKGVYE
RHDV2-MW460120  GKLLGFSKMCTLIDLTVTKGVYE
RHDV2-MW460122  GKLLGFSKMCTLIDLTVTKGVYE
RHDV2-MW460123  GKLLGFSKMCTLIDLTVTKGVYE
RHDV2-MW460127  GKLLGFSKMCTLIDLTVTKGVYE
RHDV2-MW460128  GKLLGFSKMCTLIDLTVTKGVYE
RHDV2-MW460130  GKLLGFSKMCTLIDLTVTKGVYE
RHDV2-MW460131  GKLLGFSKMCTLIDLTVTKGVYE
RHDV2-MW460135  GKLLGFSKMCTLIDLTVTKGVYE
RHDV2-MW460137  GKLLGFSKMCTLIDLTVTKGVYE
RHDV2-MW460140  GKLLGFSKMCTLIDLTVTKGVYE
RHDV2-MW460141  GKLLGFSKMCTLIDLTVTKGVYE
RHDV2-MW460142  GKLLGFSKMCTLIDLTVTKGVYE
RHDV2-MW460144  GKLLGFSKMCTLIDLTVTKGVYE
RHDV2-MW460149  GKLLGFSKMCTLIDLTVTKGVYE
RHDV2-MW460163  GKLLGFSKMCTLIDLTVTKGVYE
RHDV2-MW460169  GKLLGFSKMCTLIDLTVTKGVYE
RHDV2-MW460170  GKLLGFSKMCTLIDLTVTKGVYE
RHDV2-MW460181  GKLLGFSKMCTLIDLTVTKGVYE
RHDV2-MW460230  GKLLGFSKMCTLIDLTVTKGVYE
RHDV2-MW460236  GKLLGFSKMCTLIDLTVTKGVYE
RHDV2-MW460156  GKLLGFSKMCTLIDLTVTKGVYE
RHDV2-MW460110  GKLLGFSKMCTLIDLTVTKGVYE
RHDV2-MW460111  GKLLGFSKMCTLIDLTVTKGVYE
RHDV2-MW460117  GKLLGFSKMCTLIDLTVTKGVYE
RHDV2-MW460119  GKLLGFSKMCTLIDLTVTKGVYE
RHDV2-MW460121  GKLLGFSKMCTLIDLTVTKGVYE
RHDV2-MW460146  GKLLGFSKMCTLIDLTVTKGVYE
RHDV2-MW460151  GKLLGFSKMCTLIDLTVTKGVYE
RHDV2-MW460152  GKLLGFSKMCTLIDLTVTKGVYE
RHDV2-MW460157  GKLLGFSKMCTLIDLTVTKGVYE
RHDV2-MW460158  GKLLGFSKMCTLIDLTVTKGVYE
RHDV2-MW460159  GKLLGFSKMCTLIDLTVTKGVYE
RHDV2-MW460161  GKLLGFSKMCTLIDLTVTKGVYE
RHDV2-MW460165  GKLLGFSKMCTLIDLTVTKGVYE
RHDV2-MW460173  GKLLGFSKMCTLIDLTVTKGVYE
RHDV2-MW460175  GKLLGFSKMCTLIDLTVTKGVYE
```

```
RHDV2-MW460177  GKLLGFSKMCTLIDLTVTKGVYE
RHDV2-MW460179  GKLLGFSKMCTLIDLTVTKGVYE
RHDV2-MW460180  GKLLGFSKMCTLIDLTVTKGVYE
RHDV2-MW460183  GKLLGFSKMCTLIDLTVTKGVYE
RHDV2-MW460186  GKLLGFSKMCTLIDLTVTKGVYE
RHDV2-MW460187  GKLLGFSKMCTLIDLTVTKGVYE
RHDV2-MW460190  GKLLGFSKMCTLIDLTVTKGVYE
RHDV2-MW460193  GKLLGFSKMCTLIDLTVTKGVYE
RHDV2-MW460194  GKLLGFSKMCTLIDLTVTKGVYE
RHDV2-MW460195  GKLLGFSKMCTLIDLTVTKGVYE
RHDV2-MW460197  GKLLGFSKMCTLIDLTVTKGVYE
RHDV2-MW460198  GKLLGFSKMCTLIDLTVTKGVYE
RHDV2-MW460202  GKLLGFSKMCTLIDLTVTKGVYE
RHDV2-MW460204  GKLLGFSKMCTLIDLTVTKGVYE
RHDV2-MW460208  GKLLGFSKMCTLIDLTVTKGVYE
RHDV2-MW460210  GKLLGFSKMCTLIDLTVTKGVYE
RHDV2-MW460212  GKLLGFSKMCTLIDLTVTKGVYE
RHDV2-MW460213  GKLLGFSKMCTLIDLTVTKGVYE
RHDV2-MW460214  GKLLGFSKMCTLIDLTVTKGVYE
RHDV2-MW460217  GKLLGFSKMCTLIDLTVTKGVYE
RHDV2-MW460220  GKLLGFSKMCTLIDLTVTKGVYE
RHDV2-MW460223  GKLLGFSKMCTLIDLTVTKGVYE
RHDV2-MW460224  GKLLGFSKMCTLIDLTVTKGVYE
RHDV2-MW460226  GKLLGFSKMCTLIDLTVTKGVYE
RHDV2-MW460227  GKLLGFSKMCTLIDLTVTKGVYE
RHDV2-MW460232  GKLLGFSKMCTLIDLTVTKGVYE
RHDV2-MW460233  GKLLGFSKMCTLIDLTVTKGVYE
RCV-KX357707    GKLLGFSKMCTLIDLTVTKGVYE
RCV-KX357655    GKLLGFSKMCTLIDLTVTKGVYE
RCV-KX357696    GKLLGFSKMCTLIDLTVTKGVYE
RCV-KX357658    GKLLGFSKMCTLIDLTVTKGVYE
RCV-KX357663    GKLLGFSKMCTLIDLTVTKGVYE
RCV-KX357703    GKLLGFSKMCTLIDLTVTKGVYE
RCV-KX357705    GKLLGFSKMCTLIDLTVTKGVYE
RCV-KX357697    GKLLGFSKMCTLIDLTVTKGVYE
RCV-KX357700    GKLLGFSKMCTLIDLTVTKGVYE
RHDV2-MW460153  GKLLGFSKMCTLIDLTVTKGVYE
RHDV2-MW460155  GKLLGFSKMCTLIDLTVTKGVYE
RHDV2-MW460178  GKLLGFSKMCTLVDLTVTKGVYE
RHDV2-MW460185  GKLLGFSKMCTLVDLTVTKGVYE
RHDV2-MW460211  GKLLGFSKMCTLVDLTVTKGVYE
RCV-KX357689    GKLLGFSKMCTLIDLTVTKGIYE
RCV-KX357682    GKLLGFSKMCTLIDLTVTKGIYE
RCV-KX357691    GKLLGFSKMCTLIDLTVTKGIYE
```

```
RCV-KX357683    GKLLGFSKMCTLIDLTVTKGIYE
RCV-KX357686    GKLLGFSKMCTLIDLTVTKGIYE
RCV-KX357685    GKLLGFSKMCTLIDLTVTKGIYE
RCV-KX357692    GKLLGFSKMCTLIDLTVTKGIYE
RCV-KX357684    GKLLGFSKMCTLIDLTVTKGIYE
RCV-KX357690    GKLLGFSKMCTLIDLTVTKGIYE
RCV-KX357687    GKLLGFSKMCTLIDLTVTKGIYE
RCV-KX357701    GKLLGFSKMCTLIDLTVTKGVYE
RHDV2-MW460219  GKLLGFSKMCTLIDLTVTKGVYE
RCV-KX357693    GKLLGFSKMCTLIDLTVTKGIYE
RCV-EU871528    GKLLGFSKMCTLIDLTVTKGVYE
RCV-KX357673    GKLLGFSKMCTLIDLTVTKGVYE
RCV-KX357675    GKLLGFSKMCTLIDLTVTKGVYE
RCV-KX357694    GKLLGFSKMCTLIDLTVTKGVYE
RHDV2-MW460199  GKLLGFSKMCTLIDLTVTKGVYE
RHDV2-MW460200  GKLLGFSKMCTLIDLTVTKGVYE
RCV-KX357665    GKLLGFSKMCTLIDLTITKGVYE
RCV-KX357681    GKLLGFSKMCTLIDLTITKGVYE
RCV-KX357662    GKLLGFSKMCTLIDLTITKGVYE
RCV-KX357661    GKLLGFSKMCTLIDLTITKGVYE
RCV-KX357680    GKLLGFSKMCTLIDLTITKGVYE
RCV-KX357679    GKLLGFSKMCTLIDLTITKGVYE
RCV-KX357664    GKLLGFSKMCTLIDLTVTKGVYE
RCV-KX357674    GKLLGFSKMCTLIDLTVTKGVYE
RCV-KX357669    GKLLGFSKMCTLIDLTVTKGVYE
RCV-KX357666    GKLLGFSKMCTLIDLTVTKGVYE
RCV-KX357659    GKLLGFSKMCTLIDLTVTKGVYE
RCV-KX357676    GKLLGFSKMCTLIDLTITKGVYE
RCV-KX357660    GKLLGFSKMCTLIDLTVTKGVYE
RCV-KX357656    GKLLGFSKMCTLIDLTVTKGVYE
RHDV2-MW460150  GKLLGFSKMCTLIDLTVTKGVYE
HaCV-MK138383   GKLLGFSKMCTLIDLTLTKGVYE
HaCV-MH204883   GKLLGFSKMCTLIDCTITKGVYE
EBHSV-MK440616  GKLLGFSKMCTLIDCTITKGVYE
EBHSV-MK440613  GKLLGFSKMCTLIDCTITKGVYE
EBHSV-LR899171  GKLLGFSKMCTLIDCTITKGVYE
EBHSV-LR899152  GKLLGFSKMCTLIDCTITKGVYE
EBHSV-LR899140  GKLLGFSKMCTLIDCTITKGVYE
EBHSV-LR899188  GKLLGFSKMCTLIDCTITKGVYE
EBHSV-LR899185  GKLLGFSKMCTLIDCTITKGVYE
EBHSV-LR899182  GKLLGFSKMCTLIDCTITKGVYE
EBHSV-MK440614  GKLLGFSKMCTLVDCTITKGVYE
EBHSV-MK440615  GKLLGFSKMCTLVDCTITKGVYE
EBHSV-KC832838  GKLLGFSKMCTLIDCTITKGVYE
```

```
EBHSV-KC832839  GKLLGFSKMCTLIDCTITKGVYE
EBHSV-MK440617  GKLLGFSKMCTLIDCTITKGVYE
EBHSV-Z69620    GKLLGFSKMCTLIDCTITKGVYE
HaCV-KR230102   GKLLGFSKMCTLVDCTITKGVYE
HaCV-MK138384   GKLLGFSKMCTLIDPNITKGVYE
HaCV-MK138385   GKLLGFSKMCTLIDPSITKGVYE
                ************:* .:*:*:**
```

Tree scale: 0.1

Supplemental file S2. A high-resolution tree file

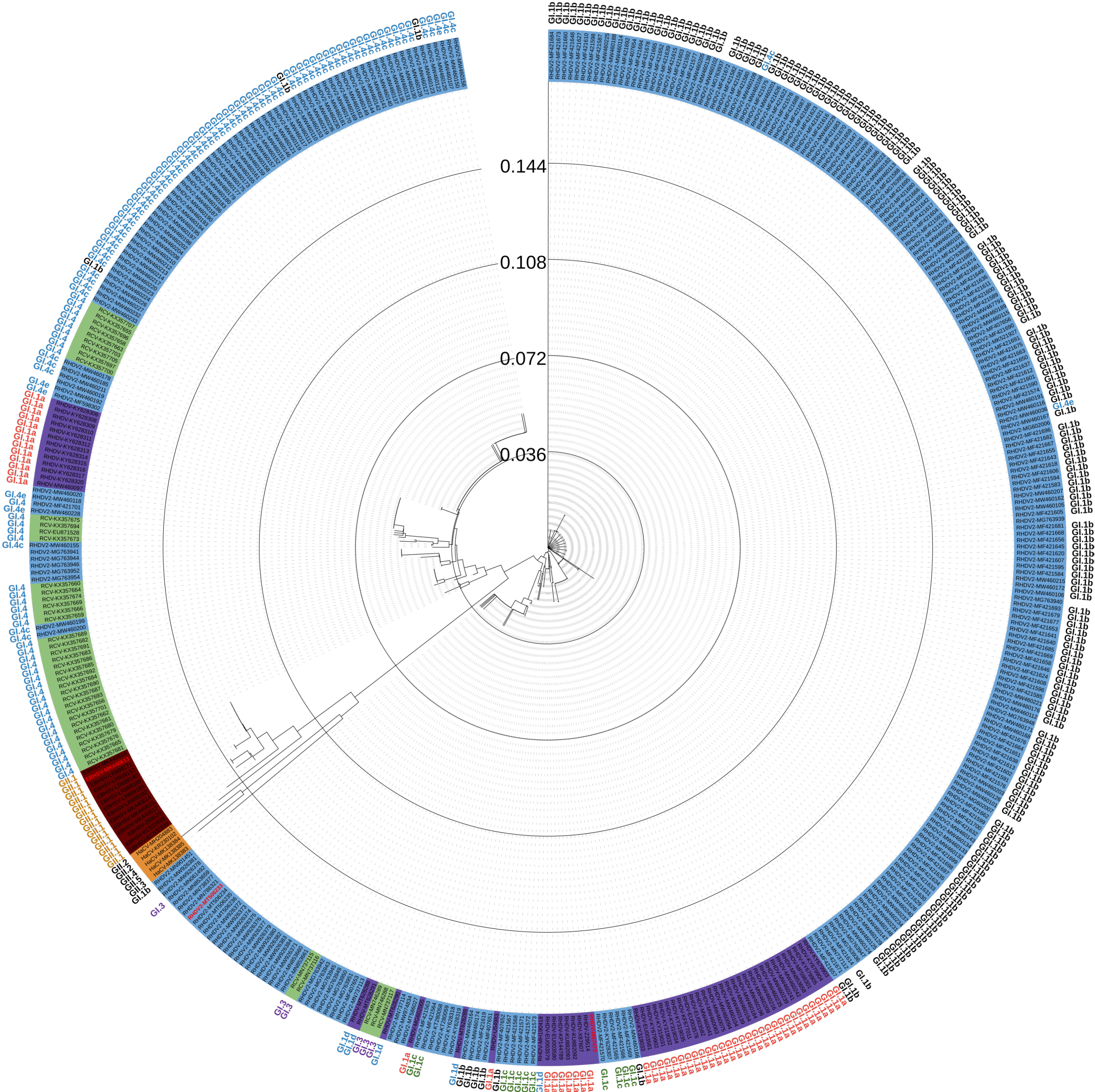

Supplement: Supplemental file 1 — Supplemental material. Download spectrum.00142-22-s0001.pdf, PDF file, 3.9 MB [file spectrum.00142-22-s0001.pdf]
